# Intravenous iron therapy results in rapid and sustained rise in myocardial iron content through a novel pathway

**DOI:** 10.1093/eurheartj/ehae359

**Published:** 2024-06-25

**Authors:** Mayra Vera-Aviles, Syeeda Nashitha Kabir, Akshay Shah, Paolo Polzella, Dillon Yee Lim, Poppy Buckley, Charlotte Ball, Dorine Swinkels, Hanke Matlung, Colin Blans, Philip Holdship, Jeremy Nugent, Edward Anderson, Michael Desborough, Stefan Piechnik, Vanessa Ferreira, Samira Lakhal-Littleton

**Affiliations:** Department of Physiology, Anatomy & Genetics, University of Oxford, Sherrington Building, Parks Road, Oxford OX1 3PT, United Kingdom; Department of Physiology, Anatomy & Genetics, University of Oxford, Sherrington Building, Parks Road, Oxford OX1 3PT, United Kingdom; Nuffield Department of Clinical Neurosciences, University of Oxford, Oxford, United Kingdom; Department of Clinical Haematology, Oxford University Hospitals NHS Foundation Trust, Oxford, United Kingdom; Department of Physiology, Anatomy & Genetics, University of Oxford, Sherrington Building, Parks Road, Oxford OX1 3PT, United Kingdom; Department of Physiology, Anatomy & Genetics, University of Oxford, Sherrington Building, Parks Road, Oxford OX1 3PT, United Kingdom; Department of Physiology, Anatomy & Genetics, University of Oxford, Sherrington Building, Parks Road, Oxford OX1 3PT, United Kingdom; Department of Laboratory Medicine, Iron Expertise Centre, Radboud University Medical Centre, Nijmegen, The Netherlands; Iron Expertise Centre, Sanquin Blood Bank, Amsterdam, The Netherlands; Sanquin Diagnostic Services, Amsterdam, The Netherlands; Department of Molecular Hematology, Sanquin Research and Landsteiner Laboratory, Amsterdam, The Netherlands; Sanquin Diagnostic Services, Amsterdam, The Netherlands; Department of Molecular Hematology, Sanquin Research and Landsteiner Laboratory, Amsterdam, The Netherlands; Department of Earth Sciences, University of Oxford, Oxford, United Kingdom; Department of Chemistry, Chemistry Research Laboratory, University of Oxford, Oxford, United Kingdom; Department of Chemistry, Chemistry Research Laboratory, University of Oxford, Oxford, United Kingdom; Department of Clinical Haematology, Oxford University Hospitals NHS Foundation Trust, Oxford, United Kingdom; Oxford Centre for Clinical Magnetic Resonance Research (OCMR), University of Oxford, Oxford, United Kingdom; Oxford Centre for Clinical Magnetic Resonance Research (OCMR), University of Oxford, Oxford, United Kingdom; Department of Physiology, Anatomy & Genetics, University of Oxford, Sherrington Building, Parks Road, Oxford OX1 3PT, United Kingdom

**Keywords:** Intravenous iron therapy, Myocardium, Labile iron, Non-transferrin-bound iron, Ferric carboxymaltose, Magnetic resonance

## Abstract

**Background and Aims:**

Intravenous iron therapies contain iron–carbohydrate complexes, designed to ensure iron becomes bioavailable via the intermediary of spleen and liver reticuloendothelial macrophages. How other tissues obtain and handle this iron remains unknown. This study addresses this question in the context of the heart.

**Methods:**

A prospective observational study was conducted in 12 patients receiving ferric carboxymaltose (FCM) for iron deficiency. Myocardial, spleen, and liver magnetic resonance relaxation times and plasma iron markers were collected longitudinally. To examine the handling of iron taken up by the myocardium, intracellular labile iron pool (LIP) was imaged in FCM-treated mice and cells.

**Results:**

In patients, myocardial relaxation time T1 dropped maximally 3 h post-FCM, remaining low 42 days later, while splenic T1 dropped maximally at 14 days, recovering by 42 days. In plasma, non-transferrin-bound iron (NTBI) peaked at 3 h, while ferritin peaked at 14 days. Changes in liver T1 diverged among patients. In mice, myocardial LIP rose 1 h and remained elevated 42 days after FCM. In cardiomyocytes, FCM exposure raised LIP rapidly. This was prevented by inhibitors of NTBI transporters T-type and L-type calcium channels and divalent metal transporter 1.

**Conclusions:**

Intravenous iron therapy with FCM delivers iron to the myocardium rapidly through NTBI transporters, independently of reticuloendothelial macrophages. This iron remains labile for weeks, reflecting the myocardium’s limited iron storage capacity. These findings challenge current notions of how the heart obtains iron from these therapies and highlight the potential for long-term dosing to cause cumulative iron build-up in the heart.

## Structured Graphical Abstract

**Structured Graphical Abstract:**
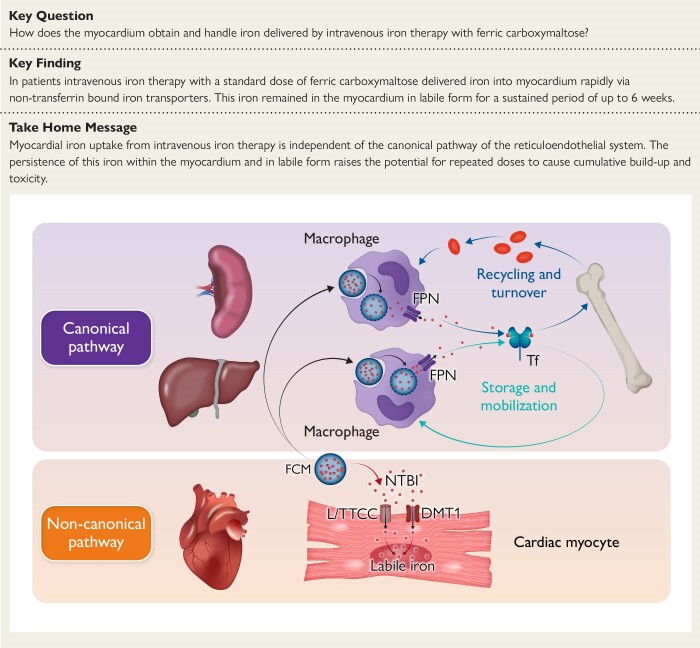
In the canonical pathway of intravenous iron metabolism, ferric carboxymaltose (FCM) is taken up by reticuloendothelial macrophages of the spleen and liver. Here, FCM is metabolized and iron liberated for export into the circulation by the iron exporter ferroportin (FPN). Upon export, this iron is loaded onto its plasma chaperone transferrin (Tf). This transferrin-bound iron is then consumed, primarily by the bone marrow for the production of red blood cells (RBCs). Once senescent, RBCs are subjected to breakdown by spleen reticuloendothelial macrophages, allowing this iron to be recycled. Iron may also be stored in hepatocytes, to be mobilized at times of increased iron demand. The heart acquires iron from FCM through a non-canonical pathway that is independent of reticuloendothelial macrophages. This is because FCM releases some of its iron directly into the circulation resulting in the appearance of non-transferrin-bound iron (NTBI). Owing to abundant expression of NTBI transporters L-type and T-type calcium channels (L/TTCC) and divalent metal transporter (DMT1), cardiac myocytes rapidly take up FCM-derived NTBI from the circulation. The iron that enters the cardiac myocytes in this manner joins the intracellular labile iron pool (LIP), which is the pool of reactive iron responsible for generating oxidative stress.

**See the editorial comment for this article ‘Is myocardial accumulation of non-transferrin-bound iron clinically relevant?’, by S. von Haehling** ***et al*** **., https://doi.org/10.1093/eurheartj/ehae560.**

## Introduction

Iron deficiency (ID) is the most common nutritional deficiency worldwide and is associated with adverse outcomes in a broad range of settings.^1–12^ Thus, tackling ID efficiently and safely holds the potential to deliver cross-cutting benefits to a large number of patients. ID has been traditionally treated with oral iron supplements, which replenish iron incrementally over weeks by delivering ∼100 mg doses into the gut lumen, of which typically 10%–22% is absorbed into the circulation.^13^ Oral iron’s gastrointestinal side effects, poor compliance, and limited absorption in inflammatory settings have fuelled a gradual shift towards intravenous (IV) iron therapies.^14–18^ These can deliver up to 2000 mg of iron into the circulation at once. To minimize the otherwise toxic effects of free iron reactivity, the iron in IV iron therapies is contained within carbohydrate complexes.^18^ These are designed for targeted uptake by macrophages of the reticuloendothelial system in the spleen and to a lesser extent in the liver.^18–20^ According to the consensus canonical pathway of IV iron metabolism, the iron–carbohydrate complex is degraded within the lysosome of macrophages, iron is freed, exported into the circulation, and loaded onto the plasma iron chaperone transferrin.^18–20^ The iron made bioavailable through this pathway is considered safe because transferrin-bound iron is non-reactive and is only taken up by tissues according to their needs. Indeed, surface expression of transferrin receptor (TfR) is coupled to cellular iron need, because its levels are controlled by the iron homeostatic proteins [iron regulatory proteins (IRPs)].^21^ Inside cells, labile iron is quickly made non-reactive through storage into ferritin, the translation of which is also controlled by IRPs.^21^ Because plasma ferritin derives primarily from macrophages, uptake of iron–carbohydrate complexes by reticuloendothelial macrophages is closely followed by a rise in plasma ferritin.^22,23^

Pharmacokinetic studies have reported a rapid rise in plasma non-transferrin-bound iron (NTBI) following treatment with IV iron, indicating that some iron is released from the carbohydrate complex directly into the circulation.^24^ What remains unknown is whether this NTBI is taken up by tissues, and the extent to which such uptake contributes to the ultimate rise in tissue iron content resulting from IV iron therapy. This is an important question because, unlike transferrin-bound iron, NTBI enters cells via non-canonical pathways that are not coupled to cellular iron needs.^25^ Because NTBI transporters are not regulated by IRPs, they continue to take up iron despite excess cellular labile iron.^25^ The heart is particularly prone to NTBI uptake due to abundant expression of certain NTBI transporters including L-type and T-type calcium channels (LTCCs and TTCCs) and divalent metal transporter 1 (DMT1).^26,27^ This, together with the heart’s relatively limited capacity to store labile iron into ferritin explains why iron-overload cardiomyopathy is a frequent and fatal complication in the iron-overload disorders of hemochromatosis and β-thalassaemia.^28–30^

Tissue iron uptake can be assessed non-invasively by magnetic resonance (MR) because the ferromagnetic properties of iron influence certain MR tissue characteristics, in particular relaxation times T1, T2, and T2*.^31^ Furthermore, the handling of iron taken up into tissues can be assessed through reporter systems that rely on labile iron reactivity.^32^ Using these approaches, the objective of the current study is to investigate myocardial uptake and handling of iron following IV iron therapy with the most widely used formulation ferric carboxymaltose (FCM).

## Methods

This report was prepared according to STROBE and ARRIVE guidelines (see Supplementary data online, *Files S1 and S2* respectively).

### Clinical study design

The Study of Tissue Iron Uptake in iron-deficient patients following IV iron therapy (STUDY) is an investigator-initiated, prospective, observational study conducted at one UK site, sponsored by the University of Oxford. The full study protocol is available in Supplementary data online, *File S3*. The trial protocol and amendments were approved by a NHS Ethics Committee in the UK (North West—Liverpool Central Research Ethics Committee Ref: 22/NW/0172), and the Health Research Authority. The study was registered prospectively on the ISRCTN registry (ISRCTN15770553) and ClinicalTrials.gov (NCT05609318). The sample size of 12 participants was not arrived at statistically, due to lack of previous information on the magnitude of acute effects of FCM on T1/T2/T2* values. Instead, the sample size of 12 was selected based on a previous exploratory study of MR imaging following IV infusion with ultrasmall superparamagnetic particles of iron oxide, which used sample sizes of 5–12 participants per group.^33^

### Patients

Patients were recruited through the ID Management Service, part of the Oxford University Hospitals NHS Foundation Trust. Patients, aged 18 years or above, who were scheduled to receive IV iron therapy as per standard clinical care for the correction of ID [ferritin < 100 µg/L and/or transferrin saturation (Tsat) < 20%] with or without anaemia (haemoglobin < 120 g/L for women and <130 g/L for men) were invited to participate. All eligible patients identified during the screening period were contacted. Following written informed consent, patients were screened for exclusion criteria, which were any of the following: any MR imaging incompatible implants, pregnant or lactating participants, acute decompensated heart failure, unstable clinical status, and any other medical conditions that would influence the reliability of the study results as determined by the investigators, any other contraindication to MR imaging. The full list of inclusion and exclusion criteria is provided within the study protocol in Supplementary data online, *File S3*.

### Study procedures

A flow chart outlining study procedures is shown in Appendix A part of study protocol provided in Supplementary data online, *File S3*.

The full cardiac MR protocol (including the standard Siemens printout) and mapping methods^34,35^ are provided in Supplementary data online, *File S4*.

Ferric carboxymaltose solution (Ferinject^®^, Vifor Pharma, Glattbrugg, Switzerland) was given as a 20 mL IV infusion (equivalent to 1000 mg of iron) diluted in a sterile saline solution (.9% *w*/*v* NaCl) and administered over 15 min. Participants were closely monitored for signs of hypersensitivity during the infusion and for at least 30 min after the treatment, as per standard clinical care.

### Study outcomes

The primary outcome was changes from baseline in multi-organ MR relaxation times T1, T2, and T2* for each participant. The secondary outcome was changes from baseline in plasma iron indices: iron, ferritin, Tsat, NTBI, and serum levels of lipid peroxidation marker malondialdehyde (MDA).

### Mice

Description of methods for mouse-based^36–38^ work is provided in Supplementary data online, *File S5*.

### Cardiac myocytes

Description of methods for cell-based work is provided in Supplementary data online, *File S5*.

### Statistical analysis

Data in figures are shown as mean ± standard error of the mean. In the text, values are reported as mean ± standard deviation.

Normality and equal variance were tested using the Shapiro–Wilk test and Levene test, respectively. Pairwise comparisons were drawn using two-tailed, unpaired *t*-test (for sample size ≥5), or Mann–Whitney test (for sample size <5). Multiple group comparisons were drawn using a one-way or two-way ANOVA followed by Dunnett’s *post hoc* test. Longitudinal repeated measure data in patients were analysed using mixed effects analysis to account for missing data, with Dunnett’s multiple comparison test. A *P*-value of <.05 was considered statistically significant. Analysis was carried out using GraphPad PRISM.10.

## Results

### Ferric carboxymaltose treatment in patients results in rapid and sustained rise in myocardial iron

**Figure 1:**
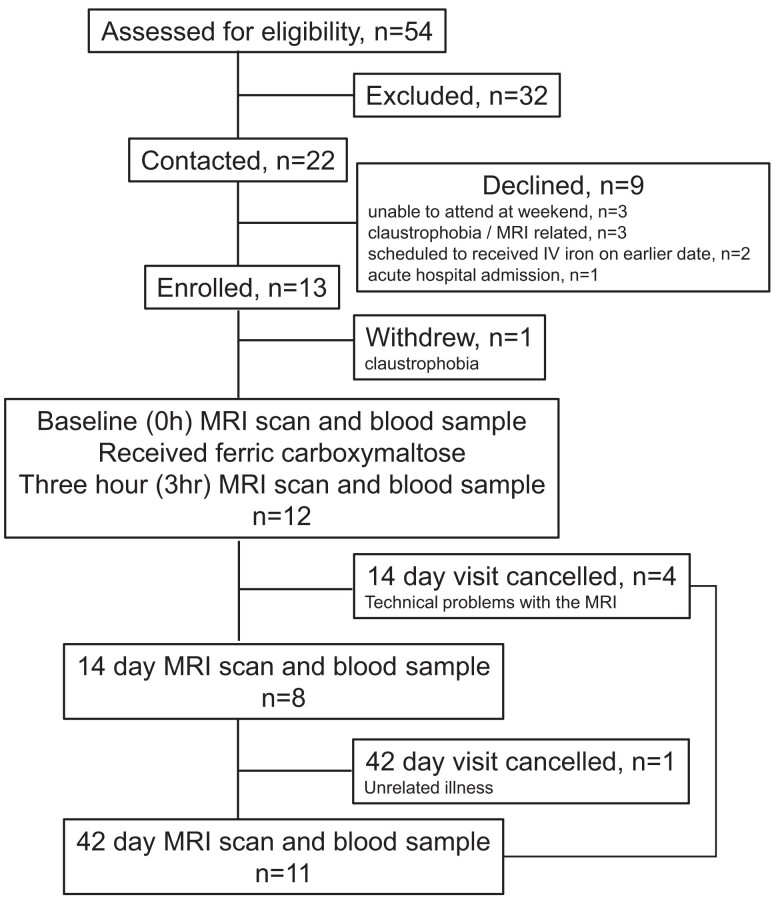
Trial profile

Between 18 October 2022 and 31 July 2023, 54 patients were screened for eligibility, of whom 13 were recruited and 12 completed the study (*Figure 1*). Patient demographics and baseline characteristics are shown in *Table 1*.

**Table 1:** Patient demographics and baseline characteristics (*n* = 12)

Characteristic	Value
**Age (years), median (IQR), [Range]**	44 (55–77) [27–69]
**Sex, *n* (%)**	
Female/Male	11 (91)/1 (9)
**Weight (kg), median (IQR)**	73.5 (56.5–77.0)
**BMI (kg/m^2^), median (IQR)**	27.2 (22.2–28.9)
**Ethnicity, *n* (%)**	
White	6 (50)
South Asian	4 (33)
Black	1 (17)
Mixed	0 (0)
Other	1 (17)
**Reason for referral, *n* (%)**	
Heavy menstrual bleeding	8 (66.8%)
Abnormal uterine bleeding (e.g. fibroids)	1 (8.3%)
Rectal bleeding	1 (8.3%)
IDA of unclear aetiology	2 (16.6%)
**Previous IV iron infusions, *n* (%)**	
None	9 (75)
One prior infusion	3 (25)
**Relevant comorbidities, *n* (%)**	
Ischaemic heart disease	0 (0)
Chronic respiratory disease (asthma and COPD)	0 (0)
**Laboratory iron parameters at referral (normal range), median (IQR)**	
Haemoglobin (120–170), g/L	105.5 (98.0–110.5)
Ferritin (10–300), mcg/L	7.4 (4.8–14.9)
Tsat (16–50), %	7 (5–8)

BMI, body mass index; COPD, chronic obstructive pulmonary disease; IDA, iron deficiency anaemia; IQR, interquartile range; IV, intravenous; SD, standard deviation; Tsat, transferrin saturation.

**Figure 2:**
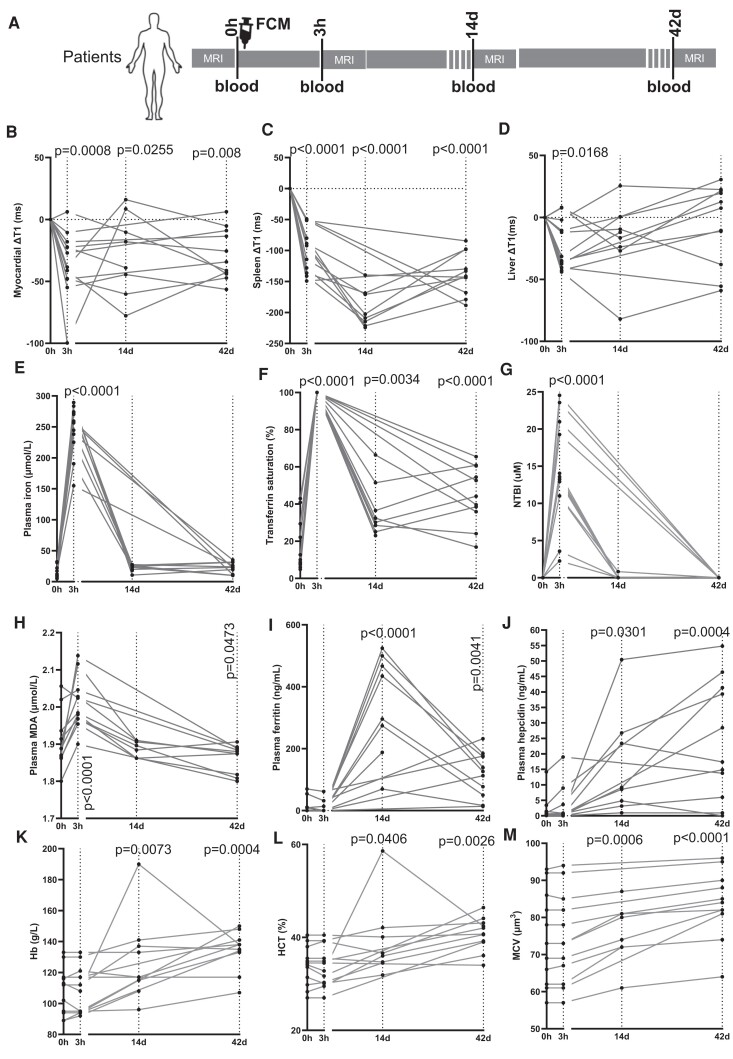
Ferric carboxymaltose treatment in patients results in rapid and sustained rise in myocardial iron. (*A*) Schematic of study design. (*B*) Longitudinal changes from baseline (0 h) in myocardial T1 (ΔT1) for each participant. (*C*) Longitudinal changes from baseline in splenic T1 (ΔT1) for each participant, (*D*) Longitudinal changes from baseline in liver T1 (ΔT1) for each participant. Per-participant longitudinal assessments of plasma iron (*E*), transferrin saturation (*F*), plasma non-transferrin-bound iron (NTBI) (*G*), plasma lipid peroxidation marker malondialdehyde (MDA) (*H*), plasma ferritin (*I*), plasma hepcidin (*J*), haemoglobin Hb (*K*), haematocrit (*L*) and mean corpuscular volume (MCV) (*M*)

To assess the short-term and long-term impacts of FCM on myocardial iron, participants were scanned immediately prior to infusion (0 h), then 3 h, 14 days, and 42 days post-infusion (*Figure 2A*). At 3 h post-infusion, myocardial T1 dropped in 11/12 participants, with a mean change (ΔT1) of −35.35 ± 26.88 ms (*P* = .0008). At 14 and 42 days post-infusion, myocardial T1 was still lower than 0 h values, with a change of −26.76 ± 32.93 ms (*P* = .0255) and −28.45 ± 20.33 ms (*P* = .008), respectively. Importantly, ΔT1 values at days 14 and 42 were not significantly different from those at 3 h post-infusion (*P* = .709 and .775, respectively) (*Figure 2B*). Thus, a single standard dose of FCM in patients with ID raises myocardial iron rapidly and maximally within 3 h, and this rise is sustained for at least 42 days post-infusion.

To understand the pathways underlying the rise in myocardial iron, we also assessed changes in spleen and liver iron, where reticuloendothelial macrophages reside. Splenic T1 dropped in all participants at 3 h post-infusion, with a mean change (ΔT1) of −39.7 ± 36.2 ms (*P* < .0001). However maximal change of −193.9 ± 30.59 ms was only seen at 14 days (*P* < .0001). By 42 days post-infusion, the mean change ΔT1 was −137 ± 33.68 ms (*P* < .0001). Between days 14 and 42, there was a statistically significant recovery in ΔT1 values (*P* = .0004) (*Figure 2C*). Thus, after a single standard dose of FCM in patients with ID, spleen iron begins to rise within 3 h, but rises maximally at 14 days, and is declining again at 42 days.

Next, we assessed T1 changes in the liver. At 3 h post-infusion, liver T1 dropped in 11/12 participants. The mean change ΔT1 was −23 ± 19.9 ms (*P* = .0168). By 14 and 42 days post-infusion, liver ΔT1 values had diverged markedly among participants, and were no longer significantly different from 0 h values (*P* = .093 and .829, respectively) (*Figure 2D*).

In terms of circulating iron, plasma iron concentration rose sharply in all participants from a mean concentration of 14.05 ± 2.52 µM at 0 h to 246.64 ± 10.60 µM at 3 h (*P* < .0001), then declined to 22.01 ± 1.14 µM at 14 days and to 22.23 ± 2.31 µM at 42 days such that plasma iron levels at these timepoints were no longer significantly higher than what they were at 0 h (*P* = .78 and .722, respectively) (*Figure 2E*).

Transferrin saturation rose sharply in all participants from 16.74 ± 3.65% at 0 h to full saturation at 3 h (*P* < .0001), then declined to 36.68 ± 3.78% at 14 days and 44.82 ± 4.12% at 42 days, though it remained significantly higher than it was at 0 h (*P* = .0034 and *P* < .0001, respectively) (*Figure 2F*).

Plasma NTBI levels rose sharply in all participants from being below detection [assay lower limit of detection (LLOD) is .607 µM] at 0 h to 14.16 ± 1.87 µM at 3 h post-FCM infusion (*P* < .0001), then declined at day 14 to below the LLOD in all participants but one, such that the mean concentration at this timepoint was .102 ± .073μM (*P* = .999 compared to 0 h). At 42 days post-infusion, NTBI levels were below the detection limit for all participants (>.999 compared to 0 h) (*Figure 2G*).

The presence of labile iron entities such as NTBI generates peroxides which in turn react with susceptible molecules, including certain lipids. Plasma MDA, a biomarker of lipid peroxidation rose in all participants from a mean of 1.908 ± .0185 µM at 0 h to 2.007 ± .0180 µM at 3 h (*P* < .0001). They declined to 1.886 ± .005 µM at day 14 such that they were no longer different from 0 h (*P* = .7816), and then declined further to 1.858 ± .010 µM at day 42 (*P* = .0473 relative to 0 h) (*Figure 2H*).

In terms of plasma ferritin, mean levels were 12.77 ± 6.27 µg/mL at 0 h and were not significantly different at 3 h post-infusion (8.925 ± 5.08 µg/mL, *P* = .9983). However, they rose to 344 ± 41.59 µg/mL at 14 days (*P* < .0001 relative 0 h) and 65.8 ± 18.99 µg/mL at 42 days (*P* = .0041 relative to 0 h). The decline in ferritin levels from day 14 to 42 was significant (*P* = .0029) (*Figure 2I*).

We also assessed the levels of the iron homeostatic hormone hepcidin, which were at 2.12 ± 1.139 ng/mL at 0 h, remaining unchanged at 3 h (3.16 ± 1.55 ng/mL, *P* = .9923) but rising significantly to 15.9 ± 4.27 ng/mL at 14 days (*P* = .0301) and further still to 23.93 ± 5.05 ng/mL at 42 days (*P* = .0004) (*Figure 2J*).

Haematological parameters, including haemoglobin, haematocrit, and mean corpuscular volume were all significantly improved at days 14 and 42 compared to 0 h (*Figure 2K–M*).

These data demonstrate that following infusion of a standard dose of FCM in iron-deficient patients, the myocardium takes up iron quickly, within 3 h, coinciding with maximal rises in serum iron availability, both transferrin and non-transferrin-bound. The iron taken up within the first 3 h is retained in the myocardium for at least 42 days, despite declining serum iron availability. This is in contrast to the spleen, where iron uptake is maximal at 14 days, subsequently declining by 42 days. Additionally, changes in plasma ferritin levels followed the pattern of changes in spleen iron.

### Ferric carboxymaltose treatment in mice results in rapid and sustained rise in myocardial labile iron pool

**Figure 3:**
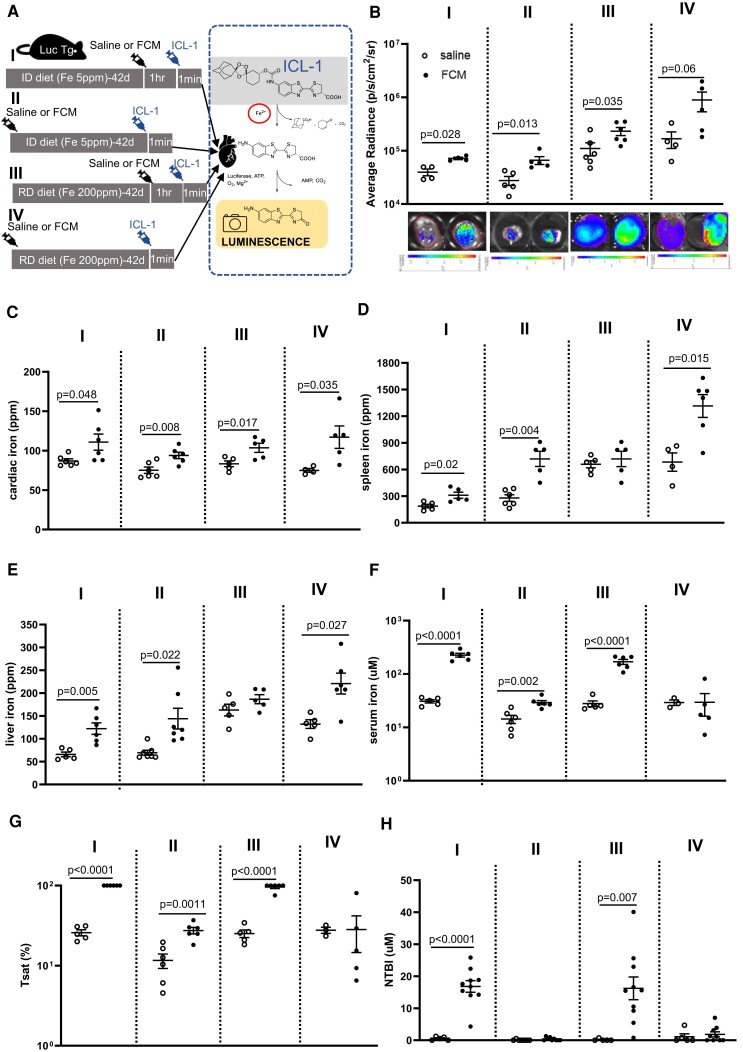
Ferric carboxymaltose treatment in mice results in rapid and sustained rise in myocardial labile iron poo (LIP)l. (*A*) Schematic of study design. ID = iron-deficient diet containing 5 parts per million (ppm) iron. RD = replete diet containing 200 ppm iron. ICL-1 = iron-caged luciferin-1. Luc Tg = mice transgenic for the luciferase reporter transgene. (*B*) Average LIP radiance in hearts of iron-deficient or iron-replete mice 1 h or 42 days after intravenous infusion of saline or ferric carboxymaltose (15 mg/kg iron). Representative luminescence images of hearts from each experimental group are also shown. (*C*) Total elemental iron concentration in the heart. (*D*) Total elemental iron concentration in the spleen. (*E*) Total elemental iron concentration in the liver. (*F*) Concentrations of iron in serum, (*G*) Transferrin saturation in serum, (*H*) Concentrations of non-transferrin-bound iron (NTBI) in serum

Having observed an increase in myocardial iron following FCM infusion in patients, we sought to determine how this iron is handled in the myocardium. To that effect, we used luciferase luminescence reporter mice, and injected them with iron-caged luciferin, which is only converted into the substrate of the enzyme luciferase in the presence of cytoplasmic labile iron. This approach was used to quantitatively assess the impact on myocardial labile iron pool (LIP) of a single IV infusion of FCM (15 mg/kg iron) into iron-replete or iron-deficient mice. Myocardial LIP was assessed either 1 h or 42 days after FCM infusion (*Figure 3A*). The iron-replete or deficient status of mice was confirmed (see Supplementary data online, *Figure S2A* and *B*).

In FCM-treated iron-deficient mice, myocardial LIP was raised both 1 h after infusion (*P* = .028 vs. saline) and 42 days after infusion (*P* = .013 vs. saline) (*Figure 3B*). In FCM-treated iron-replete mice, myocardial LIP was raised 1 h post-infusion (*P* = .035 vs. saline) and there was also a trend for the increase to be sustained 42 days after infusion (*P* = .06 vs. saline) (*Figure 3B*). Total elemental iron concentration in the heart was raised by FCM in iron-deficient mice, both at 1 h (*P* = .048 vs. saline) and 42 days after infusion (*P* = .008 vs. saline) (*Figure 3C*). They were also raised in iron-replete mice, both at 1 h (*P* = .017 vs. saline) and 42 days after infusion (*P* = .035 vs. saline) (*Figure 3C*). Of note, myocardial LIP was lower in iron-deficient mice than in iron-replete mice (*P* = .0047) (see Supplementary data online, *Figure S2C*).

As with patients, we sought to understand the pathways underpinning the rise in myocardial iron. FCM infusion into iron-deficient mice raised splenic iron within 1 h (*P* = .02 vs. saline), through the rise was much more pronounced 42 days post-infusion (*P* = .004 vs. saline). In iron-replete mice, FCM infusion did not raise spleen iron after 1 h, but did so after 42 days (*P* = .015 vs. saline) (*Figure 3D*). Changes in liver iron followed a similar trend as changes in spleen iron (*Figure 3E*). In terms of circulating iron availability, serum iron levels were raised by FCM infusion in comparison to saline in iron-deficient mice 1 h after infusion (*P* < .0001 vs. saline), and were still raised 42 days post-infusion (*P* = .0002 vs. saline). In iron-replete mice, serum iron levels were raised 1 h after FCM infusion (*P* < .0001 vs. saline) but not significantly so 42 days after infusion (*P* = .392 vs. saline) (*Figure 3F*). Changes in Tsat reflected changes in serum iron levels, with full or near full saturation seen 1 h after FCM infusion (*Figure 3G*). Consistent with this, NTBI was low or below the detection limit in saline-treated mice, but rose markedly 1 h after FCM infusion both in iron-deficient mice (*P* < .0001 vs. saline) and in iron-replete mice (*P* = .007 vs. saline). (*Figure 3H*).

FCM did not have an acute effect on serum ferritin in either iron-deficient of iron-replete mice, but raised serum ferritin in iron-deficient mice after 42 days (*P* = .0155 vs. saline) (see Supplementary data online, *Figure S2D*).

These pre-clinical data confirm the previous clinical observation that FCM results in a rapid and sustained increase in myocardial iron and further demonstrate that the iron taken up by the myocardium is in labile (reactive) form, and remains so for at least 42 days post-treatment.

### Exposure to ferric carboxymaltose raises cellular labile iron pool in cardiac myocytes via non-transferrin-bound iron transporters

**Figure 4:**
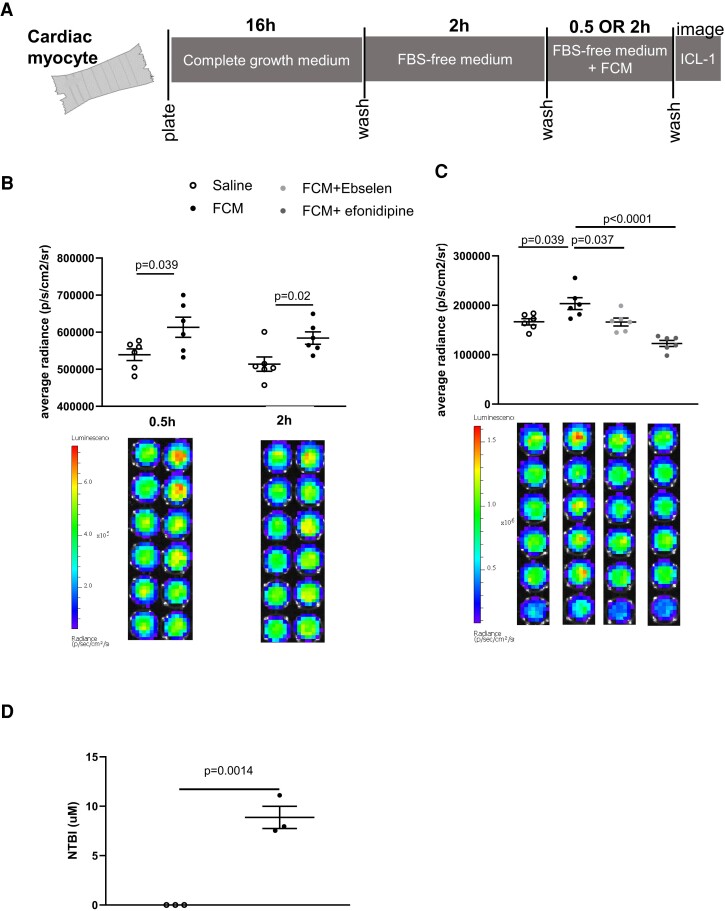
Exposure to ferric carboxymaltose raises cellular LIP in cardiac myocytes via non-transferrin-bound iron (NTBI) transporters. (*A*) Schematic of study design using rat cardiac myocytes, expressing the luciferase reporter transgene. (*B*) Average LIP radiance in cardiac myocytes after .5 h or 2 h exposure to saline or ferric carboxymaltose (.25 mg/mL iron) in growth media. Luminescence of individual wells are shown in bottom panel. (*C*) Average LIP radiance in cardiac myocytes after 1 h exposure to saline, ferric carboxymaltose (.25 mg/mL iron) alone, or in combination with ebselen (divalent metal transporter 1 inhibitor) or efonidipine (L-type and T-type calcium channel inhibitor). Luminescence of individual wells are shown in bottom panel (*D*) non-transferrin-bound iron (NTBI) concentration in supernatants of cells exposed to saline or ferric carboxymaltose (.25 mg/mL)

The rapid rise in myocardial iron following FCM infusion indicates direct myocardial uptake of iron from the circulation through a pathway that bypasses reticuloendothelial macrophages. To ascertain direct uptake and determine its route(s), we used the rat cardiac myocyte cell line H2c9, transfected via lentivirus, to express the luciferase reporter transgene (see Supplementary data online, *Figure S3*). FCM was added to the growth media at a concentration of .25 mg/mL iron to reflect plasma FCM levels following a standard dose in patients. To eliminate any confounding effects of iron from the growth media additive foetal bovine serum (FBS), cardiac myocytes were switched to FBS-free growth media 2 h before and throughout exposure to FCM or saline (*Figure 4A*).

Compared to saline treatment, FCM in growth media raised cellular LIP in cardiac myocytes at .5 h (*P* = .039 vs. saline) and 2 h (*P* = .0204 vs. saline) (*Figure 4B*). Given that this rise occurred in the absence of FBS (the source of transferrin in growth media), we postulated that it was the result of NTBI uptake. To test this hypothesis, we determined the impact of efonidipine, an LTCC and TTCC inhibitor, and ebselen, an inhibitor of the ferrous iron transporter DMT1. We found that ebselen reduced (*P* = .0372 vs. FCM alone) and efonidipine completely prevented (*P* < .0001 vs. FCM alone) the rise in cardiac myocyte LIP (*Figure 4C*).

Consistent with this, NTBI was present in the supernatants of FCM-treated cells, being below the detection limit in the supernatants of saline-treated cells (*P* = .0014) (*Figure 4D*).

These data confirm that FCM treatment raises LIP in cardiac myocytes and further demonstrate this rise is due to the uptake of extracellular NTBI derived from FCM.

## Discussion

While IV iron therapies have been suggested to raise the iron content of certain tissues, including the heart,^39^ the mechanisms underlying this rise, and the ultimate fate of the iron that is taken up remained unknown. The present study addresses these unknowns in the context of the heart, through longitudinal assessment of changes in myocardial iron relative to changes in splenic, liver, and circulating iron, and specific tracing of cellular labile iron, the immediate product of iron uptake.

The key and novel finding of the present study is that the rise in myocardial iron following a standard dose of IV iron with FCM is the result of rapid and direct uptake of FCM-derived NTBI in the circulation (*Structured Graphical Abstract*). Indeed, in iron-deficient patients and mice, myocardial iron content peaked within hours of FCM infusion, a timeframe that is too rapid to be attributed to the canonical pathway of FCM degradation by reticuloendothelial macrophages. Furthermore, splenic iron in patients, closely followed by plasma ferritin levels, only peaked at 14 days post-infusion and began to decline by 42 days post-infusion, consistent with the initial uptake of FCM into spleen macrophages and subsequent release of iron into the circulation. Acute rises in circulating iron levels, including the appearance of NTBI in patients and mice, and the presence of NTBI in the supernatants of FCM-treated cells demonstrate that FCM releases some of its iron directly into the circulation and in quantities that are sufficient to raise the iron content of the myocardium. A previous pharmacokinetic study of IV iron formulations in healthy volunteers also detected acute rises in NTBI and estimated that 10%–40% of the iron contained within FCM is released rapidly and directly into the circulation as NTBI.^24^ In cultured cardiac myocytes, exposure to a clinically relevant dose of FCM raised intracellular iron content in the absence of any macrophages, and in a manner that was primarily dependent on LTCC and/or TTCC, and to a lesser extent DMT1, recognized NTBI transporters that are abundantly expressed in the heart and known to be responsible for iron-overload cardiomyopathy in thalassaemia and hemochromatosis.^25–27^ While DMT1 is regulated by IRPs according to cellular iron needs, LTCCs and TTCCs are not regulated in this manner, continuing to take up iron into cells despite excess intracellular iron levels.

The second key and novel finding of the present study is that the iron taken up into the myocardium following FCM treatment remains in labile form for weeks. The magnitude of rise in cardiac LIP following FCM infusion was much greater than the magnitude of the corresponding rise in total cardiac iron, consistent with the notion that LIP only represents a small proportion of total cellular iron, and indicating that FCM raises cardiac LIP both in absolute terms and as a proportion of total cardiac iron. Specialized organs of iron handling, such as the spleen and liver, are characterized by high iron storage capacity and rapid iron turnover owing to abundant levels of ferritin and ferroportin, respectively.^28,29,40^ That iron taken up into the myocardium remains labile 42 days later, is consistent with the fact that the myocardium is not a specialized organ of iron handling, and has relatively limited capacity for iron storage and turnover.^28,29^ Measurements of tissue ferritin content are consistent with this notion. At 42 days post-infusion, cardiac ferritin content in iron-replete mice was not raised by FCM (see Supplementary data online, *Figure S4A*) despite a rise in total cardiac iron (*Figure 3C*), indicating this ferritin threshold represents the maximal ferritin storage capacity of the heart. Cardiac ferritin content was raised by FCM in iron-deficient mice at this timepoint, but to levels comparable to the maximal threshold observed in iron-replete mice. These data indicate that the heart can expand its ferritin storage capacity, but only up to a certain threshold, beyond which any additional iron taken up is more likely to remain within LIP. One hour post-FCM infusion, neither the heart nor the liver or the spleen raised their ferritin content, indicating that this timeframe is not sufficient to increase the cellular ferritin storage capacity (see Supplementary data online, *Figure S4A, C* and *E*). This failure to raise ferritin storage capacity likely underpins the rapid rise in cardiac LIP observed at this timepoint. Quantitatively, cardiac ferritin content is also markedly lower than that of the spleen and liver (see Supplementary data online, *Figure S4G*). The haem tissue content does not appear to be altered by FCM treatment (except in the spleen of iron-deficient mice at 42 days), indicating that iron incorporation into haem does not serve as a means of cellular iron storage.

Intriguingly, in the spleen and liver of participants, other relaxation parameters T2 and T2* echoed changes in T1, dropping significantly following FCM infusion, with maximal drops at 14 days post-infusion (see Supplementary data online, *Figure S5A–D*). In contrast, changes in myocardial T2 and T2* did not echo changes in myocardial T1 and diverged greatly between participants, with myocardial ΔT2 only trending towards a significant drop at 42 days post-infusion (*P* = .086 compared to 0 h) (see Supplementary data online, *Figure S5E and F*). The lack of concordance between myocardial relaxation parameters T1, T2, and T2* is a well-recognized phenomenon.^41–43^ T1 has been shown to have higher sensitivity than T2* for detecting milder myocardial iron loading in thalassaemia patients, and may therefore be better at resolving small increases following IV iron therapy.^41,42^ Changes in relaxation parameters reflect microscopic inhomogeneity in the magnetic field, created by the distribution of iron inside cells. Unbound labile iron has a diffuse cytoplasmic distribution, in contrast to stored iron, where up to 4500 atoms of iron are clustered inside the 80 Å diameter cavity of the ferritin molecule.^43^ Thus, it is likely that labile iron affects the magnetic field, and consequently relaxation parameters, differently from the iron stored in ferritin. Our findings are consistent with the idea that T1 better reflects labile iron entities, while T2 and T2* better reflect iron stored in ferritin. If proven, the differential effects of labile iron on T1 vs. T2 relaxation parameters could be exploited in the future to improve the non-invasive and early detection of potentially harmful cardiac iron deposition.

The findings of the current study have important clinical implications. While the increase in myocardial iron following a single dose of FCM is not of the magnitude known to cause cardiomyopathy,^44^ the lack of iron clearance from the myocardium over the period of 42 days highlights the potential for cumulative build-up with repeated doses. Based on the observed mean drop in myocardial T1 of −28.45 ms after a single IV iron dose, our calculations estimate that six doses would cumulatively lower myocardial T1 to below 850 ms, the cut-off shown to predict iron-overload cardiomyopathy^45^ (see Supplementary data online, *Table S1*). This raises pertinent questions about the potential for cardiac complications in those receiving long-term IV iron therapy. Notably, women with heavy uterine bleeding (such as those that constitute the majority of the study’s participants), represent the largest burden of chronic ID in the general population, with a higher likelihood of requiring multiple infusions over their reproductive lifetime.^46^ These implications may also extend to patients with ID that accompanies chronic disorders such as heart failure (HF) or malabsorptive disorders.^47^ Trials of long-term IV iron therapy in patients with HF (where up to nine doses were administered) have not reported an increased risk of adverse effects, through a trend for greater risk of a composite outcome of hospitalization for HF or cardiovascular death was seen in patients with Tsat ≥ 24%.^1,48–52^ Notably, myocardial iron content was not monitored in these studies, and any manifestation of cardiac iron toxicity could have been masked by the clinical signs of pre-existing heart failure. In patients on dialysis, high IV iron dosing regimens of >300 mg/month have been associated with higher mortality.^53^ In these settings, minimum and maximum ferritin cut-offs are often used, respectively, to inform the need for further IV iron doses, and to safeguard against the risk of parenchymal iron-overload. However, our findings that myocardial iron uptake following IV iron therapy is independent of reticuloendothelial macrophages indicate that they are also uncoupled from changes in plasma ferritin levels. Hence ferritin cut-offs may not be appropriate to safeguard against the risk of myocardial iron overload in patients on long-term IV iron therapy and MR-based monitoring of myocardial iron should be considered instead. Additionally, in settings where myocardial iron repletion maybe desirable, the benefits sustained by patients would be independent of changes in plasma ferritin levels.^1^

### Study strengths and limitations

The present clinical study is longitudinal, and combines data on both tissue and circulating iron levels, providing an opportunity to map the fluxes of the iron derived from FCM over time. However, these findings are derived from a relatively small number of participants. Most of these were women with ID due to heavy menstrual bleeding, and it remains to be determined if the study’s findings can be generalized to patient groups with different aetiologies of ID, including heart failure. Though not specific to this study, the use of MR relaxometry to estimate tissue iron content has its limitations. For instance, relaxometry results depend on the choice of sequence and inferences on tissue iron content hinge on a gross simplification that the relaxivity coefficients for all/any iron species in blood and tissue are similar across all study visits.^53^ Additionally, cardiac MR does not provide information as to the nature of the iron entities (labile vs. stored) or as to whether the extra iron is within cardiac myocytes or elsewhere. However, biopsies of iron-overload patients show that myocardial iron deposition is primarily sarcoplasmic within cardiac myocytes and that cardiac iron concentrations correlate well with MR relaxometry measures.^44,54^

The pre-clinical findings of the present study rely on luminescence-based imaging of labile iron. This approach has strengths and limitations. Strengths include: (i) use of an iron-caged form of luciferin that can only be converted to luciferin by reactive iron ensures the output measure (luminescence) is specifically proportional to the amount of reactive iron rather than total, ferritin or haem iron, (ii) the ability to image luminescence in whole hearts provides *in situ* assessment of myocardial LIP, overcoming any artefactual effects of tissue lysis and digestion, and (iii) reliance on luciferase transgenic mice and myocardial cells ensures reproducibility and eliminates any variations resulting from transient/unstable luciferase expression, and (iv) reliance on measuring the activity of a cytosolic enzyme eliminates any confounding effects of extracellular labile iron on LIP quantitation. Limitations include (i) this approach only assesses relative changes in LIP and does not provide absolute quantitation of it and (ii) the need to excise the hearts prior to imaging (to safeguard against interferences from surrounding tissues), has the potential to impact on luciferase enzyme activity and total luminescence signal, though this risk would have been present in all experimental groups. Other techniques used here also have intrinsic limitations, e.g. the inability of elemental iron measurements by inductively coupled plasma mass spectometry (ICP-MS) to resolve the cellular distribution of iron, the inability to quantify the iron content of ferritin peptides, and the potential interference of other plasma components, and growth medium additives with NTBI measurements.

## Supplementary Material

ehae359_Supplementary_Data

## Declarations

### Data Availability

Research data will be made available upon reasonable request to the corresponding author.

### Pre-registered Clinical Trial Number

The pre-registered clinical trial number is NCT05609318.
