# Supplementary material for: Intravenous iron therapy results in rapid and sustained rise in myocardial iron content through a novel pathway
Source: Eur Heart J. 2024 Jun 25;45(42):4497–508. doi: 10.1093/eurheartj/ehae359 (PMC11544312; doi:10.1093/eurheartj/ehae359)
Supplement: ehae359_Supplementary_Data [file ehae359_supplementary_data.zip › Supplemental File 4- CMR imaging protocol and standard Siemens printout (1).pdf]

MRI imaging protocol

CMR imaging was performed using a 1.5-Tesla MRI scanner (AvantoFit, software version VE11C, Siemens Healthcare, Erlangen, Germany) using multichannel-channel phased-array coils. The protocol included localisers followed by HASTE anatomic imaging of the torso. Cardiac planning was followed by cardiac cine for cardiac volumes and function. Quantitative imaging was performed to include the mid-ventricular short axis oblique view of the heart, and also views of liver and spleen. The full MRI protocol included ShMOLLI T1-mapping (prototype version WIP1048B), and the Siemens Myomaps product T2- and T2*-mapping sequences, organised in blocks for each view[44, 45]. Quantitative tissue characterisation imaging blocks were repeated between 4-7 (typically 6) times, for close monitoring of any changes in MR relaxometries over time, with the total scan time lasting up to 1 hour. For each subsequent visit, the prior planning was used to assure the closest practically possible planning similarity and tissue coverage between visits.

Images were analysed by manually placing regions of interest (ROIs) in T1 maps for the first set in each view and each visit. Heart was assessed as the whole myocardial ring, and separate septal and lateral wall ROIs. Spleen and liver ROIs were assessed using a single ROIs avoiding the major blood vessels. Representative T1, T2 and T2* maps with ROIs for the cardiac and liver/spleen planes are shown in supplemental figure 1. All the ROIs were automatically propagated first to other tissue characterisation sequences, and then to all repeats within each sequence and view for any one visit. All ROIs were manually checked; and any major registration errors or proximity to major artefacts were manually corrected. To protect from inevitable inclusion of partial volume within and between neighbouring tissues, we used the median value for all pixels within any ROI, and median of any ROIs for one type of tissue within a single image. Tissue estimates were averaged between repeats, resulting in a single per-patient-per-visit value.

The standard Siemens printout is provided below

Table of contents

\\USER

Cardiac Research Protocols

IV IRON

IV IRON4

trufi_loc_multi_iPAT@c FOV500
trufi_loc_multi_iPAT@c FOV500
Trans HASTE- 10mm no gap non bh
Cor HASTE- 10mm no gap non bh
trufi_2-chamber_iPAT
trufi_4-chamber_iPAT
trufi_shortaxis_iPAT
HLA tf2d15_retro_iPAT3
VLA tf2d15_retro_iPAT3
LVOT tf2d15_retro_iPAT3
SA STACK tf2d15_retro_iPAT3
VLS_ShMOLLI_192i_d11_nFilt
KLS_ShMOLLI_192i_d11_nFilt_FOV460
VLST2Map_TrueFISP
VLSShMOLLI_C2P_m
VLST2StarMap_8echo_heart
VLSFB_MOCO_gt_T2star_DB_8e_128
FS_HiFE
VLSFB_MOCO_gt_T2star_DB_8e_160
FS_LowNFE
VLSnonBH_T2StarMap_12echo_liver
KLST2Map_TrueFISP
KLSShMOLLI_C2P_m
KLST2StarMap_8echo_heart
KLSFB_MOCO_gt_T2star_DB_8e_128
FS_HiFE
KLSFB_MOCO_gt_T2star_DB_8e_160
FS_LowNFE
KLSnonBH_T2StarMap_12echo_liver

\\USER\Cardiac Research Protocols\IV IRON\IV IRON4\trufi_loc_multi_iPAT@c FOV500

TA: 7.2 s PM: ISO Voxel size: 2.0×2.0×8.0 mmPAT: 2 Rel. SNR: 1.00 : tfi

Properties

Prio recon	Off
Load images to viewer	On
Inline movie	Off
Auto store images	On
Load images to stamp segments	On
Load images to graphic segments	On
Auto open inline display	Off
Auto close inline display	Off
Start measurement without further preparation	On
Wait for user to start	Off
Start measurements	Single measurement

Routine

Slice group	1
Slices	3
Dist. factor	300 %
Position	L0.0 P0.0 H52.3 mm
Orientation	Transversal
Phase enc. dir.	A >> P
Slice group	2
Slices	3
Dist. factor	300 %
Position	L30.0 P30.0 H52.3 mm
Orientation	Sagittal
Phase enc. dir.	A >> P
Slice group	3
Slices	3
Dist. factor	300 %
Position	L0.0 P40.0 H52.3 mm
Orientation	Coronal
Phase enc. dir.	R >> L
AutoAlign	---
Phase oversampling	0 %
FoV read	500 mm
FoV phase	100.0 %
Slice thickness	8.0 mm
TR	288.36 ms
TE	1.06 ms
Averages	1
Concatenations	9
Filter	Distortion Corr.(2D), Prescan Normalize
Coil elements	BO1-3;SP1-3

Contrast - Common

TR	288.36 ms
TE	1.06 ms
TD	0 ms
Magn. preparation	None
Flip angle	80 deg
Fat suppr.	None
Wrap-up Magn.	Restore

Contrast - Dynamic

Averages	1
Averaging mode	Short term
Reconstruction	Magnitude
Measurements	1
Multiple series	Each measurement

Resolution - Common

FoV read	500 mm
FoV phase	100.0 %
Slice thickness	8.0 mm
Base resolution	256
Phase resolution	66 %
Phase partial Fourier	Off
Trajectory	Cartesian
Interpolation	Off

Resolution - iPAT

PAT mode	GRAPPA
Accel. factor PE	2
Ref. lines PE	24
Reference scan mode	Integrated

Resolution - Filter Image

Image Filter	Off
Distortion Corr.	On
Mode	2D
Unfiltered images	Off
Prescan Normalize	On
Unfiltered images	Off
Normalize	Off
B1 filter	Off

Resolution - Filter Rawdata

Raw filter	Off
Elliptical filter	Off
POCS	Off

Geometry - Common

Slice group	1
Slices	3
Dist. factor	300 %
Position	L0.0 P0.0 H52.3 mm
Orientation	Transversal
Phase enc. dir.	A >> P
Slice group	2
Slices	3
Dist. factor	300 %
Position	L30.0 P30.0 H52.3 mm
Orientation	Sagittal
Phase enc. dir.	A >> P
Slice group	3
Slices	3
Dist. factor	300 %
Position	L0.0 P40.0 H52.3 mm
Orientation	Coronal
Phase enc. dir.	R >> L
FoV read	500 mm
FoV phase	100.0 %
Slice thickness	8.0 mm
TR	288.36 ms
Multi-slice mode	Sequential
Series	Descending
Concatenations	9

Geometry - AutoAlign

Slice group	1
Position	L0.0 P0.0 H52.3 mm

Geometry - AutoAlign

Orientation	Transversal
Phase enc. dir.	A >> P
Slice group	2
Position	L30.0 P30.0 H52.3 mm
Orientation	Sagittal
Phase enc. dir.	A >> P
Slice group	3
Position	L0.0 P40.0 H52.3 mm
Orientation	Coronal
Phase enc. dir.	R >> L
AutoAlign	---
Initial Position	L0.0 P0.0 H52.3
Phase	0.0 mm
Read	0.0 mm
Shift	52.3 mm
Initial Rotation	0.00 deg
Initial Orientation	Transversal

Geometry - Saturation

Fat suppr.	None
Wrap-up Magn.	Restore
Special sat.	None

Geometry - Navigator

System - Miscellaneous

Positioning mode	ISO
Table position	H
Table position	52 mm
MSMA	S - C - T
Sagittal	R >> L
Coronal	A >> P
Transversal	F >> H
Coil Combine Mode	Adaptive Combine
Save uncombined	Off
Matrix Optimization	Off
Coil Focus	Flat
AutoAlign	---
Coil Select Mode	Default

System - Adjustments

B0 Shim mode	Tune up
Adjust with body coil	Off
Confirm freq. adjustment	Off
Assume Dominant Fat	Off
Assume Silicone	Off
Adjustment Tolerance	Auto

System - Adjust Volume

Position	Isocenter
Orientation	Transversal
Rotation	0.00 deg
A >> P	263 mm
R >> L	350 mm
F >> H	350 mm
Reset	Off

System - Tx/Rx

Frequency 1H	63.678323 MHz
Correction factor	1
Gain	High
Img. Scale Cor.	1.000
Reset	Off
? Ref. amplitude 1H	0.000 V

Physio - Signal1

1st Signal/Mode	ECG/Trigger
Average cycle	No Signal ms
Average cycle	No Signal ms
Captured cycle	-not set-
Acquisition window	800 ms
Trigger pulse	1
Trigger delay	400 ms
TR	288.36 ms
Concatenations	9
Segments	96
Phases	1
Adaptive Triggering	Off

Physio - Cardiac

Tagging	None
Magn. preparation	None
Fat suppr.	None
Dark blood	Off
FoV read	500 mm
FoV phase	100.0 %
Phase resolution	66 %
Cine	Off
Trajectory	Cartesian
Dummy heartbeats	0

Physio - PACE

Resp. control	Off
Concatenations	9

Inline - Common

Subtract	Off
Measurements	1
StdDev	Off
Save original images	On

Inline - Cardiac

Inline Evaluation	Off
Magn. preparation	None
Contrasts	1
TE	1.06 ms
TR	288.36 ms
Save original images	On

Inline - MIP

MIP-Sag	Off
MIP-Cor	Off
MIP-Tra	Off
MIP-Time	Off
Save original images	On

Inline - Composing

Inline Composing	Off
Distortion Corr.	On
Mode	2D
Unfiltered images	Off

Sequence - Part 1

Introduction	Off
Dimension	2D
Reordering	Linear
Asymmetric echo	Weak
Contrasts	1
Optimization	Min. TE

Sequence - Part 1

Multi-slice mode	Sequential
Echo spacing	2.5 ms
Sequence type	Trufi
Bandwidth	1149 Hz/Px

Sequence - Part 2

Define	Shots
Shots per slice	1
Segments	96
Trufi delta freq.	0 Hz
RF pulse type	Fast
Gradient mode	Fast
Excitation	Slice-sel.
Flip angle mode	Constant
Cine	Off

Sequence - Assistant

Mode	Min flip angle
Min flip angle	50 deg
Allowed delay	0 s

\\USER\Cardiac Research Protocols\IV IRON\IV IRON4\trufi_loc_multi_iPAT@c FOV500
TA: 7.2 s PM: ISO Voxel size: 2.0×2.0×8.0 mmPAT: 2 Rel. SNR: 1.00 : tfi

Properties

Prio recon	Off
Load images to viewer	On
Inline movie	Off
Auto store images	On
Load images to stamp segments	On
Load images to graphic segments	On
Auto open inline display	Off
Auto close inline display	Off
Start measurement without further preparation	Off
Wait for user to start	Off
Start measurements	Single measurement

Routine

Slice group	1
Slices	3
Dist. factor	300 %
Position	L0.0 A30.0 H0.0 mm
Orientation	Transversal
Phase enc. dir.	A >> P
Slice group	2
Slices	3
Dist. factor	300 %
Position	L30.0 P0.0 H0.0 mm
Orientation	Sagittal
Phase enc. dir.	A >> P
Slice group	3
Slices	3
Dist. factor	300 %
Position	L0.0 P10.0 H0.0 mm
Orientation	Coronal
Phase enc. dir.	R >> L
AutoAlign	---
Phase oversampling	0 %
FoV read	500 mm
FoV phase	100.0 %
Slice thickness	8.0 mm
TR	288.36 ms
TE	1.06 ms
Averages	1
Concatenations	9
Filter	Distortion Corr.(2D), Prescan Normalize
Coil elements	BO1-3;SP1-3

Contrast - Common

TR	288.36 ms
TE	1.06 ms
TD	0 ms
Magn. preparation	None
Flip angle	80 deg
Fat suppr.	None
Wrap-up Magn.	Restore

Contrast - Dynamic

Averages	1
Averaging mode	Short term
Reconstruction	Magnitude
Measurements	1
Multiple series	Each measurement

Resolution - Common

FoV read	500 mm
FoV phase	100.0 %
Slice thickness	8.0 mm
Base resolution	256
Phase resolution	66 %
Phase partial Fourier	Off
Trajectory	Cartesian
Interpolation	Off

Resolution - iPAT

PAT mode	GRAPPA
Accel. factor PE	2
Ref. lines PE	24
Reference scan mode	Integrated

Resolution - Filter Image

Image Filter	Off
Distortion Corr.	On
Mode	2D
Unfiltered images	Off
Prescan Normalize	On
Unfiltered images	Off
Normalize	Off
B1 filter	Off

Resolution - Filter Rawdata

Raw filter	Off
Elliptical filter	Off
POCS	Off

Geometry - Common

Slice group	1
Slices	3
Dist. factor	300 %
Position	L0.0 A30.0 H0.0 mm
Orientation	Transversal
Phase enc. dir.	A >> P
Slice group	2
Slices	3
Dist. factor	300 %
Position	L30.0 P0.0 H0.0 mm
Orientation	Sagittal
Phase enc. dir.	A >> P
Slice group	3
Slices	3
Dist. factor	300 %
Position	L0.0 P10.0 H0.0 mm
Orientation	Coronal
Phase enc. dir.	R >> L
FoV read	500 mm
FoV phase	100.0 %
Slice thickness	8.0 mm
TR	288.36 ms
Multi-slice mode	Sequential
Series	Descending
Concatenations	9

Geometry - AutoAlign

Slice group	1
Position	L0.0 A30.0 H0.0 mm

Geometry - AutoAlign

Orientation	Transversal
Phase enc. dir.	A >> P
Slice group	2
Position	L30.0 P0.0 H0.0 mm
Orientation	Sagittal
Phase enc. dir.	A >> P
Slice group	3
Position	L0.0 P10.0 H0.0 mm
Orientation	Coronal
Phase enc. dir.	R >> L
AutoAlign	---
Initial Position	L0.0 A30.0 H0.0
Phase	-30.0 mm
Read	0.0 mm
Shift	0.0 mm
Initial Rotation	0.00 deg
Initial Orientation	Transversal

Geometry - Saturation

Fat suppr.	None
Wrap-up Magn.	Restore
Special sat.	None

Geometry - Navigator

System - Miscellaneous

Positioning mode	ISO
Table position	H
Table position	0 mm
MSMA	S - C - T
Sagittal	R >> L
Coronal	A >> P
Transversal	F >> H
Coil Combine Mode	Adaptive Combine
Save uncombined	Off
Matrix Optimization	Off
Coil Focus	Flat
AutoAlign	---
Coil Select Mode	Default

System - Adjustments

B0 Shim mode	Tune up
Adjust with body coil	Off
Confirm freq. adjustment	Off
Assume Dominant Fat	Off
Assume Silicone	Off
Adjustment Tolerance	Auto

System - Adjust Volume

Position	Isocenter
Orientation	Transversal
Rotation	0.00 deg
A >> P	263 mm
R >> L	350 mm
F >> H	350 mm
Reset	Off

System - Tx/Rx

Frequency 1H	63.678323 MHz
Correction factor	1
Gain	High
Img. Scale Cor.	1.000
Reset	Off
? Ref. amplitude 1H	0.000 V

Physio - Signal1

1st Signal/Mode	ECG/Trigger
Average cycle	No Signal ms
Average cycle	No Signal ms
Captured cycle	-not set-
Acquisition window	800 ms
Trigger pulse	1
Trigger delay	400 ms
TR	288.36 ms
Concatenations	9
Segments	96
Phases	1
Adaptive Triggering	Off

Physio - Cardiac

Tagging	None
Magn. preparation	None
Fat suppr.	None
Dark blood	Off
FoV read	500 mm
FoV phase	100.0 %
Phase resolution	66 %
Cine	Off
Trajectory	Cartesian
Dummy heartbeats	0

Physio - PACE

Resp. control	Off
Concatenations	9

Inline - Common

Subtract	Off
Measurements	1
StdDev	Off
Save original images	On

Inline - Cardiac

Inline Evaluation	Off
Magn. preparation	None
Contrasts	1
TE	1.06 ms
TR	288.36 ms
Save original images	On

Inline - MIP

MIP-Sag	Off
MIP-Cor	Off
MIP-Tra	Off
MIP-Time	Off
Save original images	On

Inline - Composing

Inline Composing	Off
Distortion Corr.	On
Mode	2D
Unfiltered images	Off

Sequence - Part 1

Introduction	Off
Dimension	2D
Reordering	Linear
Asymmetric echo	Weak
Contrasts	1
Optimization	Min. TE

Sequence - Part 1

Multi-slice mode	Sequential
Echo spacing	2.5 ms
Sequence type	Trufi
Bandwidth	1149 Hz/Px

Sequence - Part 2

Define	Shots
Shots per slice	1
Segments	96
Trufi delta freq.	0 Hz
RF pulse type	Fast
Gradient mode	Fast
Excitation	Slice-sel.
Flip angle mode	Constant
Cine	Off

Sequence - Assistant

Mode	Min flip angle
Min flip angle	50 deg
Allowed delay	0 s

\\USER\Cardiac Research Protocols\IV IRON\IV IRON4\Trans HASTE- 10mm no gap non bh
TA: 0:56 PM: REF Voxel size: 1.4×1.4×10.0 mmPAT: Off Rel. SNR: 1.00 : h

Properties

Prio recon	Off
Load images to viewer	On
Inline movie	Off
Auto store images	On
Load images to stamp segments	On
Load images to graphic segments	On
Auto open inline display	Off
Auto close inline display	Off
Start measurement without further preparation	Off
Wait for user to start	On
Start measurements	Single measurement

Routine

Slice group	1
Slices	35
Dist. factor	0 %
Position	L0.0 A49.4 H32.4 mm
Orientation	Transversal
Phase enc. dir.	A >> P
AutoAlign	---
Phase oversampling	0 %
FoV read	360 mm
FoV phase	78.1 %
Slice thickness	10.0 mm
TR	800.0 ms
TE	26 ms
Averages	1
Concatenations	2
Filter	Distortion Corr.(2D), Prescan Normalize, Elliptical filter, Image Filter
Coil elements	BO1-3;SP1-4

Contrast - Common

TR	800.0 ms
TE	26 ms
TD	0.0 ms
MTC	Off
Magn. preparation	None
Flip angle	160 deg
Fat suppr.	None
Water suppr.	None
Restore magn.	Off

Contrast - Dynamic

Averages	1
Averaging mode	Long term
Reconstruction	Magnitude
Measurements	1
Multiple series	Off

Resolution - Common

FoV read	360 mm
FoV phase	78.1 %
Slice thickness	10.0 mm
Base resolution	256
Phase resolution	59 %
Phase partial Fourier	5/8
Interpolation	Off

Resolution - iPAT

PAT mode	None

Resolution - Filter Image

Image Filter	On
! Intensity	Medium
Edge Enhancement	1
Smoothing	1
Unfiltered images	Off
Distortion Corr.	On
Mode	2D
Unfiltered images	Off
Prescan Normalize	On
Unfiltered images	Off
Normalize	Off
B1 filter	Off

Resolution - Filter Rawdata

Raw filter	Off
Elliptical filter	On

Geometry - Common

Slice group	1
Slices	35
Dist. factor	0 %
Position	L0.0 A49.4 H32.4 mm
Orientation	Transversal
Phase enc. dir.	A >> P
FoV read	360 mm
FoV phase	78.1 %
Slice thickness	10.0 mm
TR	800.0 ms
Multi-slice mode	Single shot
Series	Interleaved
Concatenations	2

Geometry - AutoAlign

Slice group	1
Position	L0.0 A49.4 H32.4 mm
Orientation	Transversal
Phase enc. dir.	A >> P
AutoAlign	---
Initial Position	L0.0 A49.4 H18.4
Phase	-49.4 mm
Read	0.0 mm
Shift	18.4 mm
Initial Rotation	0.00 deg
Initial Orientation	Transversal

Geometry - Saturation

Fat suppr.	None
Water suppr.	None
Restore magn.	Off
Special sat.	None

Geometry - Navigator

System - Miscellaneous

Positioning mode	REF
Table position	H
Table position	14 mm

System - Miscellaneous

MSMA	S - C - T
Sagittal	R >> L
Coronal	A >> P
Transversal	F >> H
Coil Combine Mode	Adaptive Combine
Save uncombined	Off
Matrix Optimization	Off
Coil Focus	Flat
AutoAlign	---
Coil Select Mode	Default

System - Adjustments

B0 Shim mode	Tune up
Adjust with body coil	On
Confirm freq. adjustment	Off
Assume Dominant Fat	Off
Assume Silicone	Off
Adjustment Tolerance	Auto

System - Adjust Volume

Position	Isocenter
Orientation	Transversal
Rotation	0.00 deg
A >> P	263 mm
R >> L	350 mm
F >> H	350 mm
Reset	Off

System - Tx/Rx

Frequency 1H	63.678323 MHz
Correction factor	1
Gain	High
Img. Scale Cor.	1.000
Reset	Off
? Ref. amplitude 1H	0.000 V

Physio - Signal1

1st Signal/Mode	ECG/Trigger
Average cycle	No Signal ms
Average cycle	No Signal ms
Captured cycle	-not set-
Acquisition window	800 ms
Trigger pulse	2
Trigger delay	0 ms
TR	800.0 ms
Concatenations	2
Phases	1

Physio - Cardiac

Magn. preparation	None
Fat suppr.	None
Dark blood	On
Dark blood thickness	200 %
FoV read	360 mm
FoV phase	78.1 %
Phase resolution	59 %

Physio - PACE

Resp. control	Off
Concatenations	2

Inline - Common

Subtract	Off

Inline - Common

Measurements	1
StdDev	Off
Save original images	On

Inline - MIP

MIP-Sag	Off
MIP-Cor	Off
MIP-Tra	Off
MIP-Time	Off
Save original images	On

Inline - Composing

Inline Composing	Off
Distortion Corr.	On
Mode	2D
Unfiltered images	Off

Sequence - Part 1

Introduction	Off
Dimension	2D
Contrasts	1
Flow comp.	No
Multi-slice mode	Single shot
Echo spacing	2.92 ms
Bandwidth	781 Hz/Px

Sequence - Part 2

RF pulse type	Fast
Gradient mode	Fast
Turbo factor	118

Sequence - Assistant

Mode	Off
Allowed delay	30 s

\\USER\Cardiac Research Protocols\IV IRON\IV IRON4\Cor HASTE- 10mm no gap non bh
TA: 0:27 PM: REF Voxel size: 1.8×1.8×10.0 mmPAT: Off Rel. SNR: 1.00 : h

Properties

Prio recon	Off
Load images to viewer	On
Inline movie	Off
Auto store images	On
Load images to stamp segments	On
Load images to graphic segments	On
Auto open inline display	Off
Auto close inline display	Off
Start measurement without further preparation	Off
Wait for user to start	On
Start measurements	Single measurement

Routine

Slice group	1
Slices	17
Dist. factor	0 %
Position	L4.7 P16.3 F10.4 mm
Orientation	Coronal
Phase enc. dir.	R >> L
AutoAlign	---
Phase oversampling	0 %
FoV read	460 mm
FoV phase	78.1 %
Slice thickness	10.0 mm
TR	800.0 ms
TE	26 ms
Averages	1
Concatenations	2
Filter	Distortion Corr.(2D), Prescan Normalize, Elliptical filter, Image Filter
Coil elements	B1-5;SP3-6

Contrast - Common

TR	800.0 ms
TE	26 ms
TD	0.0 ms
MTC	Off
Magn. preparation	None
Flip angle	160 deg
Fat suppr.	None
Water suppr.	None
Restore magn.	Off

Contrast - Dynamic

Averages	1
Averaging mode	Long term
Reconstruction	Magnitude
Measurements	1
Multiple series	Off

Resolution - Common

FoV read	460 mm
FoV phase	78.1 %
Slice thickness	10.0 mm
Base resolution	256
Phase resolution	59 %
Phase partial Fourier	5/8
Interpolation	Off

Resolution - iPAT

PAT mode	None

Resolution - Filter Image

Image Filter	On
! Intensity	Medium
Edge Enhancement	1
Smoothing	1
Unfiltered images	Off
Distortion Corr.	On
Mode	2D
Unfiltered images	Off
Prescan Normalize	On
Unfiltered images	Off
Normalize	Off
B1 filter	Off

Resolution - Filter Rawdata

Raw filter	Off
Elliptical filter	On

Geometry - Common

Slice group	1
Slices	17
Dist. factor	0 %
Position	L4.7 P16.3 F10.4 mm
Orientation	Coronal
Phase enc. dir.	R >> L
FoV read	460 mm
FoV phase	78.1 %
Slice thickness	10.0 mm
TR	800.0 ms
Multi-slice mode	Single shot
Series	Interleaved
Concatenations	2

Geometry - AutoAlign

Slice group	1
Position	L4.7 P16.3 F10.4 mm
Orientation	Coronal
Phase enc. dir.	R >> L
AutoAlign	---
Initial Position	L4.7 P16.3 F24.4
Phase	4.7 mm
Read	24.4 mm
Shift	16.3 mm
Initial Rotation	0.00 deg
Initial Orientation	Coronal

Geometry - Saturation

Fat suppr.	None
Water suppr.	None
Restore magn.	Off
Special sat.	None

Geometry - Navigator

System - Miscellaneous

Positioning mode	REF
Table position	H
Table position	14 mm

System - Miscellaneous

MSMA	S - C - T
Sagittal	R >> L
Coronal	A >> P
Transversal	F >> H
Coil Combine Mode	Adaptive Combine
Save uncombined	Off
Matrix Optimization	Off
Coil Focus	Flat
AutoAlign	---
Coil Select Mode	Default

System - Adjustments

B0 Shim mode	Tune up
Adjust with body coil	On
Confirm freq. adjustment	Off
Assume Dominant Fat	Off
Assume Silicone	Off
Adjustment Tolerance	Auto

System - Adjust Volume

! Position	L7.9 P14.3 F13.0 mm
! Orientation	Transversal
! Rotation	-0.63 deg
! A >> P	176 mm
! R >> L	350 mm
! F >> H	457 mm
Reset	Off

System - Tx/Rx

Frequency 1H	63.678323 MHz
Correction factor	1
Gain	High
Img. Scale Cor.	1.000
Reset	Off
? Ref. amplitude 1H	0.000 V

Physio - Signal1

1st Signal/Mode	ECG/Trigger
Average cycle	No Signal ms
Average cycle	No Signal ms
Captured cycle	-not set-
Acquisition window	800 ms
Trigger pulse	2
Trigger delay	0 ms
TR	800.0 ms
Concatenations	2
Phases	1

Physio - Cardiac

Magn. preparation	None
Fat suppr.	None
Dark blood	On
Dark blood thickness	200 %
FoV read	460 mm
FoV phase	78.1 %
Phase resolution	59 %

Physio - PACE

Resp. control	Off
Concatenations	2

Inline - Common

Subtract	Off

Inline - Common

Measurements	1
StdDev	Off
Save original images	On

Inline - MIP

MIP-Sag	Off
MIP-Cor	Off
MIP-Tra	Off
MIP-Time	Off
Save original images	On

Inline - Composing

Inline Composing	Off
Distortion Corr.	On
Mode	2D
Unfiltered images	Off

Sequence - Part 1

Introduction	Off
Dimension	2D
Contrasts	1
Flow comp.	No
Multi-slice mode	Single shot
Echo spacing	2.92 ms
Bandwidth	781 Hz/Px

Sequence - Part 2

RF pulse type	Fast
Gradient mode	Fast
Turbo factor	118

Sequence - Assistant

Mode	Off
Allowed delay	30 s

\\USER\Cardiac Research Protocols\IV IRON\IV IRON4\trufi_2-chamber_iPAT

TA: 0.8 s PM: REF Voxel size: 1.5×1.5×8.0 mmPAT: 2 Rel. SNR: 1.00 : tfi

Properties

Prio recon	Off
Load images to viewer	On
Inline movie	Off
Auto store images	On
Load images to stamp segments	Off
Load images to graphic segments	On
Auto open inline display	Off
Auto close inline display	Off
Start measurement without further preparation	Off
Wait for user to start	Off
Start measurements	Single measurement

Routine

Slice group	1
Slices	1
Dist. factor	20 %
Position	R4.2 P39.2 H108.8 mm
Orientation	T > S12.4 > C-6.2
Phase enc. dir.	A >> P
AutoAlign	---
Phase oversampling	0 %
FoV read	380 mm
FoV phase	87.5 %
Slice thickness	8.0 mm
TR	260.22 ms
TE	1.16 ms
Averages	1
Concatenations	1
Filter	Distortion Corr.(2D), Prescan Normalize
Coil elements	B1,2;SP3,4

Contrast - Common

TR	260.22 ms
TE	1.16 ms
Magn. preparation	None
Flip angle	80 deg
Fat suppr.	None
Wrap-up Magn.	Restore

Contrast - Dynamic

Averages	1
Averaging mode	Short term
Reconstruction	Magnitude
Measurements	1
Multiple series	Each measurement

Resolution - Common

FoV read	380 mm
FoV phase	87.5 %
Slice thickness	8.0 mm
Base resolution	256
Phase resolution	64 %
Phase partial Fourier	Off
Trajectory	Cartesian
Interpolation	Off

Resolution - iPAT

PAT mode	GRAPPA

Resolution - iPAT

Accel. factor PE	2
Ref. lines PE	24
Reference scan mode	Integrated

Resolution - Filter Image

Image Filter	Off
Distortion Corr.	On
Mode	2D
Unfiltered images	Off
Prescan Normalize	On
Unfiltered images	Off
Normalize	Off
B1 filter	Off

Resolution - Filter Rawdata

Raw filter	Off
Elliptical filter	Off
POCS	Off

Geometry - Common

Slice group	1
Slices	1
Dist. factor	20 %
Position	R4.2 P39.2 H108.8 mm
Orientation	T > S12.4 > C-6.2
Phase enc. dir.	A >> P
FoV read	380 mm
FoV phase	87.5 %
Slice thickness	8.0 mm
TR	260.22 ms
Multi-slice mode	Sequential
Series	Interleaved
Concatenations	1

Geometry - AutoAlign

Slice group	1
Position	R4.2 P39.2 H108.8 mm
Orientation	T > S12.4 > C-6.2
Phase enc. dir.	A >> P
AutoAlign	---
Initial Position	R4.2 P39.2 H56.8
Phase	32.9 mm
Read	-8.1 mm
Shift	60.3 mm
Initial Rotation	1.35 deg
Initial Orientation	T > S
T > S	12.4
> C	-6.2

Geometry - Saturation

Fat suppr.	None
Wrap-up Magn.	Restore
Special sat.	None

Geometry - Navigator

System - Miscellaneous

Positioning mode	REF
Table position	H
Table position	52 mm

System - Miscellaneous

MSMA	S - C - T
Sagittal	R >> L
Coronal	A >> P
Transversal	F >> H
Coil Combine Mode	Adaptive Combine
Save uncombined	Off
Matrix Optimization	Off
Coil Focus	Flat
AutoAlign	---
Coil Select Mode	Default

System - Adjustments

B0 Shim mode	Tune up
Adjust with body coil	Off
Confirm freq. adjustment	Off
Assume Dominant Fat	Off
Assume Silicone	Off
Adjustment Tolerance	Auto

System - Adjust Volume

Position	Isocenter
Orientation	Transversal
Rotation	0.00 deg
A >> P	263 mm
R >> L	350 mm
F >> H	350 mm
Reset	Off

System - Tx/Rx

Frequency 1H	63.678323 MHz
Correction factor	1
Gain	High
Img. Scale Cor.	1.000
Reset	Off
? Ref. amplitude 1H	0.000 V

Physio - Signal1

1st Signal/Mode	ECG/Trigger
Average cycle	No Signal ms
Average cycle	No Signal ms
Captured cycle	-not set-
Acquisition window	800 ms
Trigger pulse	1
Trigger delay	400 ms
TR	260.22 ms
Concatenations	1
Segments	84
Phases	1
Adaptive Triggering	Off

Physio - Cardiac

Tagging	None
Magn. preparation	None
Fat suppr.	None
Dark blood	Off
FoV read	380 mm
FoV phase	87.5 %
Phase resolution	64 %
Cine	Off
Trajectory	Cartesian
Dummy heartbeats	0

Physio - PACE

Resp. control	Off

Physio - PACE

Concatenations	1

Inline - Common

Subtract	Off
Measurements	1
StdDev	Off
Save original images	On

Inline - Cardiac

Inline Evaluation	Off
Magn. preparation	None
Contrasts	1
TE	1.16 ms
TR	260.22 ms
Save original images	On

Inline - MIP

MIP-Sag	Off
MIP-Cor	Off
MIP-Tra	Off
MIP-Time	Off
Save original images	On

Inline - Composing

Inline Composing	Off
Distortion Corr.	On
Mode	2D
Unfiltered images	Off

Sequence - Part 1

Introduction	Off
Dimension	2D
Reordering	Linear
Asymmetric echo	Weak
Contrasts	1
Optimization	Min. TE
Multi-slice mode	Sequential
Echo spacing	2.7 ms
Sequence type	Trufi
Bandwidth	1149 Hz/Px

Sequence - Part 2

Define	Shots
Shots per slice	1
Segments	84
Trufi delta freq.	0 Hz
RF pulse type	Fast
Gradient mode	Fast
Excitation	Slice-sel.
Flip angle mode	Constant
Cine	Off

Sequence - Assistant

Mode	Min flip angle
Min flip angle	50 deg
Allowed delay	0 s

\\USER\Cardiac Research Protocols\IV IRON\IV IRON4\trufi_4-chamber_iPAT

TA: 0.8 s PM: REF Voxel size: 1.5×1.5×8.0 mmPAT: 2 Rel. SNR: 1.00 : tfi

Properties

Prio recon	Off
Load images to viewer	On
Inline movie	Off
Auto store images	On
Load images to stamp segments	Off
Load images to graphic segments	On
Auto open inline display	Off
Auto close inline display	Off
Start measurement without further preparation	Off
Wait for user to start	Off
Start measurements	Single measurement

Routine

Slice group	1
Slices	1
Dist. factor	20 %
Position	R9.1 P7.9 F6.1 mm
Orientation	T > S-14.6 > C12.4
Phase enc. dir.	A >> P
AutoAlign	---
Phase oversampling	0 %
FoV read	380 mm
FoV phase	93.8 %
Slice thickness	8.0 mm
TR	275.34 ms
TE	1.16 ms
Averages	1
Concatenations	1
Filter	Distortion Corr.(2D), Prescan Normalize
Coil elements	B2-4;SP4,5

Contrast - Common

TR	275.34 ms
TE	1.16 ms
Magn. preparation	None
Flip angle	80 deg
Fat suppr.	None
Wrap-up Magn.	Restore

Contrast - Dynamic

Averages	1
Averaging mode	Short term
Reconstruction	Magnitude
Measurements	1
Multiple series	Each measurement

Resolution - Common

FoV read	380 mm
FoV phase	93.8 %
Slice thickness	8.0 mm
Base resolution	256
Phase resolution	60 %
Phase partial Fourier	Off
Trajectory	Cartesian
Interpolation	Off

Resolution - iPAT

PAT mode	GRAPPA

Resolution - iPAT

Accel. factor PE	2
Ref. lines PE	24
Reference scan mode	Integrated

Resolution - Filter Image

Image Filter	Off
Distortion Corr.	On
Mode	2D
Unfiltered images	Off
Prescan Normalize	On
Unfiltered images	Off
Normalize	Off
B1 filter	Off

Resolution - Filter Rawdata

Raw filter	Off
Elliptical filter	Off
POCS	Off

Geometry - Common

Slice group	1
Slices	1
Dist. factor	20 %
Position	R9.1 P7.9 F6.1 mm
Orientation	T > S-14.6 > C12.4
Phase enc. dir.	A >> P
FoV read	380 mm
FoV phase	93.8 %
Slice thickness	8.0 mm
TR	275.34 ms
Multi-slice mode	Sequential
Series	Interleaved
Concatenations	1

Geometry - AutoAlign

Slice group	1
Position	R9.1 P7.9 F6.1 mm
Orientation	T > S-14.6 > C12.4
Phase enc. dir.	A >> P
AutoAlign	---
Initial Position	R9.1 P7.9 F6.1
Phase	6.0 mm
Read	7.3 mm
Shift	-9.7 mm
Initial Rotation	3.20 deg
Initial Orientation	T > S
T > S	-14.6
> C	12.4

Geometry - Saturation

Fat suppr.	None
Wrap-up Magn.	Restore
Special sat.	None

Geometry - Navigator

System - Miscellaneous

Positioning mode	REF
Table position	H
Table position	0 mm

System - Miscellaneous

MSMA	S - C - T
Sagittal	R >> L
Coronal	A >> P
Transversal	F >> H
Coil Combine Mode	Adaptive Combine
Save uncombined	Off
Matrix Optimization	Off
Coil Focus	Flat
AutoAlign	---
Coil Select Mode	Default

System - Adjustments

B0 Shim mode	Tune up
Adjust with body coil	Off
Confirm freq. adjustment	Off
Assume Dominant Fat	Off
Assume Silicone	Off
Adjustment Tolerance	Auto

System - Adjust Volume

Position	Isocenter
Orientation	Transversal
Rotation	0.00 deg
A >> P	263 mm
R >> L	350 mm
F >> H	350 mm
Reset	Off

System - Tx/Rx

Frequency 1H	63.678323 MHz
Correction factor	1
Gain	High
Img. Scale Cor.	1.000
Reset	Off
? Ref. amplitude 1H	0.000 V

Physio - Signal1

1st Signal/Mode	ECG/Trigger
Average cycle	No Signal ms
Average cycle	No Signal ms
Captured cycle	-not set-
Acquisition window	800 ms
Trigger pulse	1
Trigger delay	400 ms
TR	275.34 ms
Concatenations	1
Segments	84
Phases	1
Adaptive Triggering	Off

Physio - Cardiac

Tagging	None
Magn. preparation	None
Fat suppr.	None
Dark blood	Off
FoV read	380 mm
FoV phase	93.8 %
Phase resolution	60 %
Cine	Off
Trajectory	Cartesian
Dummy heartbeats	0

Physio - PACE

Resp. control	Off

Physio - PACE

Concatenations	1

Inline - Common

Subtract	Off
Measurements	1
StdDev	Off
Save original images	On

Inline - Cardiac

Inline Evaluation	Off
Magn. preparation	None
Contrasts	1
TE	1.16 ms
TR	275.34 ms
Save original images	On

Inline - MIP

MIP-Sag	Off
MIP-Cor	Off
MIP-Tra	Off
MIP-Time	Off
Save original images	On

Inline - Composing

Inline Composing	Off
Distortion Corr.	On
Mode	2D
Unfiltered images	Off

Sequence - Part 1

Introduction	Off
Dimension	2D
Reordering	Linear
Asymmetric echo	Weak
Contrasts	1
Optimization	Min. TE
Multi-slice mode	Sequential
Echo spacing	2.7 ms
Sequence type	Trufi
Bandwidth	1149 Hz/Px

Sequence - Part 2

Define	Shots
Shots per slice	1
Segments	84
Trufi delta freq.	0 Hz
RF pulse type	Fast
Gradient mode	Fast
Excitation	Slice-sel.
Flip angle mode	Constant
Cine	Off

Sequence - Assistant

Mode	Min flip angle
Min flip angle	50 deg
Allowed delay	0 s

\\USER\Cardiac Research Protocols\IV IRON\IV IRON4\trufi_shortaxis_iPAT
TA: 5.4 s PM: REF Voxel size: 1.5×1.5×8.0 mmPAT: 2 Rel. SNR: 1.00 : tfi

Properties

Prio recon	Off
Load images to viewer	On
Inline movie	Off
Auto store images	On
Load images to stamp segments	Off
Load images to graphic segments	On
Auto open inline display	Off
Auto close inline display	Off
Start measurement without further preparation	Off
Wait for user to start	Off
Start measurements	Single measurement

Routine

Slice group	1
Slices	7
Dist. factor	100 %
Position	R10.3 P40.1 F36.0 mm
Orientation	Coronal
Phase enc. dir.	R >> L
AutoAlign	---
Phase oversampling	0 %
FoV read	380 mm
FoV phase	87.5 %
Slice thickness	8.0 mm
TR	275.34 ms
TE	1.16 ms
Averages	1
Concatenations	7
Filter	Distortion Corr.(2D), Prescan Normalize
Coil elements	B1-5;SP3-6

Contrast - Common

TR	275.34 ms
TE	1.16 ms
TD	0 ms
Magn. preparation	None
Flip angle	80 deg
Fat suppr.	None
Wrap-up Magn.	Restore

Contrast - Dynamic

Averages	1
Averaging mode	Short term
Reconstruction	Magnitude
Measurements	1
Multiple series	Each measurement

Resolution - Common

FoV read	380 mm
FoV phase	87.5 %
Slice thickness	8.0 mm
Base resolution	256
Phase resolution	64 %
Phase partial Fourier	Off
Trajectory	Cartesian
Interpolation	Off

Resolution - iPAT

PAT mode	GRAPPA
Accel. factor PE	2
Ref. lines PE	24
Reference scan mode	Integrated

Resolution - Filter Image

Image Filter	Off
Distortion Corr.	On
Mode	2D
Unfiltered images	Off
Prescan Normalize	On
Unfiltered images	Off
Normalize	Off
B1 filter	Off

Resolution - Filter Rawdata

Raw filter	Off
Elliptical filter	Off
POCS	Off

Geometry - Common

Slice group	1
Slices	7
Dist. factor	100 %
Position	R10.3 P40.1 F36.0 mm
Orientation	Coronal
Phase enc. dir.	R >> L
FoV read	380 mm
FoV phase	87.5 %
Slice thickness	8.0 mm
TR	275.34 ms
Multi-slice mode	Sequential
Series	Descending
Concatenations	7

Geometry - AutoAlign

Slice group	1
Position	R10.3 P40.1 F36.0 mm
Orientation	Coronal
Phase enc. dir.	R >> L
AutoAlign	---
Initial Position	R10.3 P40.1 F88.0
Phase	-10.3 mm
Read	88.0 mm
Shift	40.1 mm
Initial Rotation	0.00 deg
Initial Orientation	Coronal

Geometry - Saturation

Fat suppr.	None
Wrap-up Magn.	Restore
Special sat.	None

Geometry - Navigator

System - Miscellaneous

Positioning mode	REF
Table position	H
Table position	52 mm
MSMA	S - C - T

System - Miscellaneous

Sagittal	R >> L
Coronal	A >> P
Transversal	F >> H
Coil Combine Mode	Adaptive Combine
Save uncombined	Off
Matrix Optimization	Off
Coil Focus	Flat
AutoAlign	---
Coil Select Mode	Default

System - Adjustments

B0 Shim mode	Tune up
Adjust with body coil	Off
Confirm freq. adjustment	Off
Assume Dominant Fat	Off
Assume Silicone	Off
Adjustment Tolerance	Auto

System - Adjust Volume

Position	Isocenter
Orientation	Transversal
Rotation	0.00 deg
A >> P	263 mm
R >> L	350 mm
F >> H	350 mm
Reset	Off

System - Tx/Rx

Frequency 1H	63.678323 MHz
Correction factor	1
Gain	High
Img. Scale Cor.	1.000
Reset	Off
? Ref. amplitude 1H	0.000 V

Physio - Signal1

1st Signal/Mode	ECG/Trigger
Average cycle	No Signal ms
Average cycle	No Signal ms
Captured cycle	-not set-
Acquisition window	774 ms
Trigger pulse	1
Trigger delay	498 ms
TR	275.34 ms
Concatenations	7
Segments	84
Phases	1
Adaptive Triggering	Off

Physio - Cardiac

Tagging	None
Magn. preparation	None
Fat suppr.	None
Dark blood	Off
FoV read	380 mm
FoV phase	87.5 %
Phase resolution	64 %
Cine	Off
Trajectory	Cartesian
Dummy heartbeats	0

Physio - PACE

Resp. control	Off
Concatenations	7

Inline - Common

Subtract	Off
Measurements	1
StdDev	Off
Save original images	On

Inline - Cardiac

Inline Evaluation	Off
Magn. preparation	None
Contrasts	1
TE	1.16 ms
TR	275.34 ms
Save original images	On

Inline - MIP

MIP-Sag	Off
MIP-Cor	Off
MIP-Tra	Off
MIP-Time	Off
Save original images	On

Inline - Composing

Inline Composing	Off
Distortion Corr.	On
Mode	2D
Unfiltered images	Off

Sequence - Part 1

Introduction	Off
Dimension	2D
Reordering	Linear
Asymmetric echo	Weak
Contrasts	1
Optimization	Min. TE
Multi-slice mode	Sequential
Echo spacing	2.7 ms
Sequence type	Trufi
Bandwidth	1149 Hz/Px

Sequence - Part 2

Define	Shots
Shots per slice	1
Segments	84
Trufi delta freq.	0 Hz
RF pulse type	Fast
Gradient mode	Fast
Excitation	Slice-sel.
Flip angle mode	Constant
Cine	Off

Sequence - Assistant

Mode	Min flip angle
Min flip angle	50 deg
Allowed delay	0 s

\\USER\Cardiac Research Protocols\IV IRON\IV IRON4\HLA tf2d15_retro_iPAT3
TA: 5.0 s PM: REF Voxel size: 1.9×1.9×8.0 mmPAT: 3 Rel. SNR: 1.00 : tfi

Properties

Prio recon	Off
Load images to viewer	On
Inline movie	On
Auto store images	On
Load images to stamp segments	Off
Load images to graphic segments	On
Auto open inline display	Off
Auto close inline display	Off
Start measurement without further preparation	Off
Wait for user to start	Off
Start measurements	Single measurement

Routine

Slice group	1
Slices	1
Dist. factor	25 %
Position	L46.1 A28.2 H1.7 mm
Orientation	T > C28.3 > S5.5
Phase enc. dir.	A >> P
AutoAlign	---
Phase oversampling	0 %
FoV read	360 mm
FoV phase	100.0 %
Slice thickness	8.0 mm
TR	40.65 ms
TE	1.15 ms
Averages	1
Concatenations	1
Filter	Distortion Corr.(2D), Prescan Normalize, Image Filter
Coil elements	B2-4;SP4,5

Contrast - Common

TR	40.65 ms
TE	1.15 ms
Magn. preparation	None
Flip angle	53 deg
Fat suppr.	None
Wrap-up Magn.	Restore

Contrast - Dynamic

Averages	1
Averaging mode	Short term
Reconstruction	Magnitude
Measurements	1
Multiple series	Each slice

Resolution - Common

FoV read	360 mm
FoV phase	100.0 %
Slice thickness	8.0 mm
Base resolution	192
Phase resolution	100 %
Phase partial Fourier	Off
Trajectory	Cartesian
View sharing	Off
Interpolation	Off

Resolution - iPAT

PAT mode	GRAPPA
Accel. factor PE	3
Ref. lines PE	24
Reference scan mode	GRE/separate

Resolution - Filter Image

Image Filter	On
! Intensity	Medium
Edge Enhancement	2
Smoothing	2
Unfiltered images	Off
Distortion Corr.	On
Mode	2D
Unfiltered images	Off
Prescan Normalize	On
Unfiltered images	Off
Normalize	Off
B1 filter	Off

Resolution - Filter Rawdata

Raw filter	Off
Elliptical filter	Off
POCS	Off

Geometry - Common

Slice group	1
Slices	1
Dist. factor	25 %
Position	L46.1 A28.2 H1.7 mm
Orientation	T > C28.3 > S5.5
Phase enc. dir.	A >> P
FoV read	360 mm
FoV phase	100.0 %
Slice thickness	8.0 mm
TR	40.65 ms
Multi-slice mode	Sequential
Series	Base To Apex
Concatenations	1

Geometry - AutoAlign

Slice group	1
Position	L46.1 A28.2 H1.7 mm
Orientation	T > C28.3 > S5.5
Phase enc. dir.	A >> P
AutoAlign	---
Initial Position	L46.1 A28.2 H1.7
Phase	-20.4 mm
Read	-48.9 mm
Shift	10.4 mm
Initial Rotation	4.22 deg
Initial Orientation	T > C
T > C	28.3
> S	5.5

Geometry - Saturation

Fat suppr.	None
Wrap-up Magn.	Restore
Special sat.	None

Geometry - Navigator

System - Miscellaneous

Positioning mode	REF
Table position	H
Table position	0 mm
MSMA	S - C - T
Sagittal	R >> L
Coronal	A >> P
Transversal	F >> H
Coil Combine Mode	Adaptive Combine
Save uncombined	Off
Matrix Optimization	Off
Coil Focus	Flat
AutoAlign	---
Coil Select Mode	Default

System - Adjustments

B0 Shim mode	Tune up
Adjust with body coil	On
Confirm freq. adjustment	Off
Assume Dominant Fat	Off
Assume Silicone	Off
Adjustment Tolerance	Auto

System - Adjust Volume

Position	Isocenter
Orientation	Transversal
Rotation	0.00 deg
A >> P	263 mm
R >> L	350 mm
F >> H	350 mm
Reset	Off

System - Tx/Rx

Frequency 1H	63.678323 MHz
Correction factor	1
Gain	High
Img. Scale Cor.	1.000
Reset	Off
? Ref. amplitude 1H	0.000 V

Physio - Signal1

1st Signal/Mode	ECG/Retro
Average cycle	No Signal ms
Average cycle	No Signal ms
Calculated phases	25
TR	40.65 ms
Concatenations	1
Segments	15
Arrhythmia detection	None

Physio - Cardiac

Tagging	None
Magn. preparation	None
Fat suppr.	None
Dark blood	Off
FoV read	360 mm
FoV phase	100.0 %
Phase resolution	100 %
Cine	On
Trajectory	Cartesian
View sharing	Off
Dummy heartbeats	1

Physio - PACE

Resp. control	Breath-hold
Concatenations	1

Inline - Common

Subtract	Off
Measurements	1
StdDev	Off
Save original images	On

Inline - Cardiac

Inline Evaluation	Off
Magn. preparation	None
Contrasts	1
TE	1.15 ms
TR	40.65 ms
Save original images	On

Inline - MIP

MIP-Sag	Off
MIP-Cor	Off
MIP-Tra	Off
MIP-Time	Off
Save original images	On

Inline - Composing

Inline Composing	Off
Distortion Corr.	On
Mode	2D
Unfiltered images	Off

Sequence - Part 1

Introduction	Off
Dimension	2D
Reordering	Linear
Asymmetric echo	Weak
Contrasts	1
Optimization	Min. TE TR
Multi-slice mode	Sequential
Echo spacing	2.7 ms
Sequence type	Trufi
Bandwidth	930 Hz/Px

Sequence - Part 2

Define	Segments
Segments	15
Trufi delta freq.	0 Hz
RF pulse type	Fast
Gradient mode	Fast*
Excitation	Slice-sel.
Flip angle mode	Constant
Cine	On

Sequence - Assistant

Mode	Off
Allowed delay	0 s

\\USER\Cardiac Research Protocols\IV IRON\IV IRON4\VLA tf2d15_retro_iPAT3
TA: 6.0 s PM: REF Voxel size: 1.9×1.9×8.0 mmPAT: 3 Rel. SNR: 1.00 : tfi

Properties

Prio recon	Off
Load images to viewer	On
Inline movie	On
Auto store images	On
Load images to stamp segments	Off
Load images to graphic segments	On
Auto open inline display	Off
Auto close inline display	Off
Start measurement without further preparation	Off
Wait for user to start	Off
Start measurements	Single measurement

Routine

Slice group	1
Slices	1
Dist. factor	25 %
Position	L13.6 P5.0 H2.3 mm
Orientation	C > S-41.6 > T-12.5
Phase enc. dir.	R >> L
AutoAlign	---
Phase oversampling	0 %
FoV read	360 mm
FoV phase	100.0 %
Slice thickness	8.0 mm
TR	40.65 ms
TE	1.15 ms
Averages	1
Concatenations	1
Filter	Distortion Corr.(2D), Prescan Normalize, Image Filter
Coil elements	B1-5;SP3-6

Contrast - Common

TR	40.65 ms
TE	1.15 ms
Magn. preparation	None
Flip angle	53 deg
Fat suppr.	None
Wrap-up Magn.	Restore

Contrast - Dynamic

Averages	1
Averaging mode	Short term
Reconstruction	Magnitude
Measurements	1
Multiple series	Each slice

Resolution - Common

FoV read	360 mm
FoV phase	100.0 %
Slice thickness	8.0 mm
Base resolution	192
Phase resolution	100 %
Phase partial Fourier	Off
Trajectory	Cartesian
View sharing	Off
Interpolation	Off

Resolution - iPAT

PAT mode	GRAPPA
Accel. factor PE	3
Ref. lines PE	24
Reference scan mode	GRE/separate

Resolution - Filter Image

Image Filter	On
! Intensity	Medium
Edge Enhancement	2
Smoothing	2
Unfiltered images	Off
Distortion Corr.	On
Mode	2D
Unfiltered images	Off
Prescan Normalize	On
Unfiltered images	Off
Normalize	Off
B1 filter	Off

Resolution - Filter Rawdata

Raw filter	Off
Elliptical filter	Off
POCS	Off

Geometry - Common

Slice group	1
Slices	1
Dist. factor	25 %
Position	L13.6 P5.0 H2.3 mm
Orientation	C > S-41.6 > T-12.5
Phase enc. dir.	R >> L
FoV read	360 mm
FoV phase	100.0 %
Slice thickness	8.0 mm
TR	40.65 ms
Multi-slice mode	Sequential
Series	Base To Apex
Concatenations	1

Geometry - AutoAlign

Slice group	1
Position	L13.6 P5.0 H2.3 mm
Orientation	C > S-41.6 > T-12.5
Phase enc. dir.	R >> L
AutoAlign	---
Initial Position	L13.6 P5.0 H2.3
Phase	6.8 mm
Read	1.0 mm
Shift	13.0 mm
Initial Rotation	4.22 deg
Initial Orientation	C > S
C > S	-41.6
> T	-12.5

Geometry - Saturation

Fat suppr.	None
Wrap-up Magn.	Restore
Special sat.	None

Geometry - Navigator

System - Miscellaneous

Positioning mode	REF
Table position	H
Table position	0 mm
MSMA	S - C - T
Sagittal	R >> L
Coronal	A >> P
Transversal	F >> H
Coil Combine Mode	Adaptive Combine
Save uncombined	Off
Matrix Optimization	Off
Coil Focus	Flat
AutoAlign	---
Coil Select Mode	Default

System - Adjustments

B0 Shim mode	Tune up
Adjust with body coil	On
Confirm freq. adjustment	Off
Assume Dominant Fat	Off
Assume Silicone	Off
Adjustment Tolerance	Auto

System - Adjust Volume

Position	Isocenter
Orientation	Transversal
Rotation	0.00 deg
A >> P	263 mm
R >> L	350 mm
F >> H	350 mm
Reset	Off

System - Tx/Rx

Frequency 1H	63.678323 MHz
Correction factor	1
Gain	High
Img. Scale Cor.	1.000
Reset	Off
? Ref. amplitude 1H	0.000 V

Physio - Signal1

1st Signal/Mode	ECG/Retro
Average cycle	No Signal ms
Average cycle	No Signal ms
Calculated phases	25
TR	40.65 ms
Concatenations	1
Segments	15
Arrhythmia detection	None

Physio - Cardiac

Tagging	None
Magn. preparation	None
Fat suppr.	None
Dark blood	Off
FoV read	360 mm
FoV phase	100.0 %
Phase resolution	100 %
Cine	On
Trajectory	Cartesian
View sharing	Off
Dummy heartbeats	1

Physio - PACE

Resp. control	Breath-hold

Physio - PACE

Concatenations	1

Inline - Common

Subtract	Off
Measurements	1
StdDev	Off
Save original images	On

Inline - Cardiac

Inline Evaluation	Off
Magn. preparation	None
Contrasts	1
TE	1.15 ms
TR	40.65 ms
Save original images	On

Inline - MIP

MIP-Sag	Off
MIP-Cor	Off
MIP-Tra	Off
MIP-Time	Off
Save original images	On

Inline - Composing

Inline Composing	Off
Distortion Corr.	On
Mode	2D
Unfiltered images	Off

Sequence - Part 1

Introduction	Off
Dimension	2D
Reordering	Linear
Asymmetric echo	Weak
Contrasts	1
Optimization	Min. TE TR
Multi-slice mode	Sequential
Echo spacing	2.7 ms
Sequence type	Trufi
Bandwidth	930 Hz/Px

Sequence - Part 2

Define	Segments
Segments	15
Trufi delta freq.	0 Hz
RF pulse type	Fast
Gradient mode	Fast*
Excitation	Slice-sel.
Flip angle mode	Constant
Cine	On

Sequence - Assistant

Mode	Off
Allowed delay	0 s

\\USER\Cardiac Research Protocols\IV IRON\IV IRON4\LVOT tf2d15_retro_iPAT3

TA: 6.0 s PM: REF Voxel size: 1.9×1.9×8.0 mmPAT: 3 Rel. SNR: 1.00 : tfi

Properties

Prio recon	Off
Load images to viewer	On
Inline movie	On
Auto store images	On
Load images to stamp segments	Off
Load images to graphic segments	On
Auto open inline display	Off
Auto close inline display	Off
Start measurement without further preparation	Off
Wait for user to start	Off
Start measurements	Single measurement

Routine

Slice group	1
Slices	1
Dist. factor	25 %
Position	L12.6 P6.7 H15.9 mm
Orientation	T > S-17.0 > C-0.4
Phase enc. dir.	A >> P
AutoAlign	---
Phase oversampling	0 %
FoV read	360 mm
FoV phase	100.0 %
Slice thickness	8.0 mm
TR	40.65 ms
TE	1.15 ms
Averages	1
Concatenations	1
Filter	Distortion Corr.(2D), Prescan Normalize, Image Filter
Coil elements	B2,3;SP4

Contrast - Common

TR	40.65 ms
TE	1.15 ms
Magn. preparation	None
Flip angle	53 deg
Fat suppr.	None
Wrap-up Magn.	Restore

Contrast - Dynamic

Averages	1
Averaging mode	Short term
Reconstruction	Magnitude
Measurements	1
Multiple series	Each slice

Resolution - Common

FoV read	360 mm
FoV phase	100.0 %
Slice thickness	8.0 mm
Base resolution	192
Phase resolution	100 %
Phase partial Fourier	Off
Trajectory	Cartesian
View sharing	Off
Interpolation	Off

Resolution - iPAT

PAT mode	GRAPPA
Accel. factor PE	3
Ref. lines PE	24
Reference scan mode	GRE/separate

Resolution - Filter Image

Image Filter	On
! Intensity	Medium
Edge Enhancement	2
Smoothing	2
Unfiltered images	Off
Distortion Corr.	On
Mode	2D
Unfiltered images	Off
Prescan Normalize	On
Unfiltered images	Off
Normalize	Off
B1 filter	Off

Resolution - Filter Rawdata

Raw filter	Off
Elliptical filter	Off
POCS	Off

Geometry - Common

Slice group	1
Slices	1
Dist. factor	25 %
Position	L12.6 P6.7 H15.9 mm
Orientation	T > S-17.0 > C-0.4
Phase enc. dir.	A >> P
FoV read	360 mm
FoV phase	100.0 %
Slice thickness	8.0 mm
TR	40.65 ms
Multi-slice mode	Sequential
Series	Base To Apex
Concatenations	1

Geometry - AutoAlign

Slice group	1
Position	L12.6 P6.7 H15.9 mm
Orientation	T > S-17.0 > C-0.4
Phase enc. dir.	A >> P
AutoAlign	---
Initial Position	L12.6 P6.7 H15.9
Phase	7.1 mm
Read	-6.9 mm
Shift	18.9 mm
Initial Rotation	4.22 deg
Initial Orientation	T > S
T > S	-17.0
> C	-0.4

Geometry - Saturation

Fat suppr.	None
Wrap-up Magn.	Restore
Special sat.	None

Geometry - Navigator

System - Miscellaneous

Positioning mode	REF
Table position	H
Table position	0 mm
MSMA	S - C - T
Sagittal	R >> L
Coronal	A >> P
Transversal	F >> H
Coil Combine Mode	Adaptive Combine
Save uncombined	Off
Matrix Optimization	Off
Coil Focus	Flat
AutoAlign	---
Coil Select Mode	Default

System - Adjustments

B0 Shim mode	Tune up
Adjust with body coil	On
Confirm freq. adjustment	Off
Assume Dominant Fat	Off
Assume Silicone	Off
Adjustment Tolerance	Auto

System - Adjust Volume

Position	Isocenter
Orientation	Transversal
Rotation	0.00 deg
A >> P	263 mm
R >> L	350 mm
F >> H	350 mm
Reset	Off

System - Tx/Rx

Frequency 1H	63.678323 MHz
Correction factor	1
Gain	High
Img. Scale Cor.	1.000
Reset	Off
? Ref. amplitude 1H	0.000 V

Physio - Signal1

1st Signal/Mode	ECG/Retro
Average cycle	No Signal ms
Average cycle	No Signal ms
Calculated phases	25
TR	40.65 ms
Concatenations	1
Segments	15
Arrhythmia detection	None

Physio - Cardiac

Tagging	None
Magn. preparation	None
Fat suppr.	None
Dark blood	Off
FoV read	360 mm
FoV phase	100.0 %
Phase resolution	100 %
Cine	On
Trajectory	Cartesian
View sharing	Off
Dummy heartbeats	1

Physio - PACE

Resp. control	Breath-hold

Physio - PACE

Concatenations	1

Inline - Common

Subtract	Off
Measurements	1
StdDev	Off
Save original images	On

Inline - Cardiac

Inline Evaluation	Off
Magn. preparation	None
Contrasts	1
TE	1.15 ms
TR	40.65 ms
Save original images	On

Inline - MIP

MIP-Sag	Off
MIP-Cor	Off
MIP-Tra	Off
MIP-Time	Off
Save original images	On

Inline - Composing

Inline Composing	Off
Distortion Corr.	On
Mode	2D
Unfiltered images	Off

Sequence - Part 1

Introduction	Off
Dimension	2D
Reordering	Linear
Asymmetric echo	Weak
Contrasts	1
Optimization	Min. TE TR
Multi-slice mode	Sequential
Echo spacing	2.7 ms
Sequence type	Trufi
Bandwidth	930 Hz/Px

Sequence - Part 2

Define	Segments
Segments	15
Trufi delta freq.	0 Hz
RF pulse type	Fast
Gradient mode	Fast*
Excitation	Slice-sel.
Flip angle mode	Constant
Cine	On

Sequence - Assistant

Mode	Off
Allowed delay	0 s

\\USER\Cardiac Research Protocols\IV IRON\IV IRON4\SA STACK tf2d15_retro_iPAT3

TA: 2:35 PM: REF Voxel size: 1.9×1.9×8.0 mmPAT: 3 Rel. SNR: 1.00 : tfi

Properties

Prio recon	Off
Load images to viewer	On
Inline movie	On
Auto store images	On
Load images to stamp segments	Off
Load images to graphic segments	On
Auto open inline display	Off
Auto close inline display	Off
Start measurement without further preparation	Off
Wait for user to start	Off
Start measurements	Single measurement

Routine

Slice group	1
Slices	11
Dist. factor	25 %
Position	L54.9 A12.1 F10.8 mm
Orientation	S > C31.4 > T16.5
Phase enc. dir.	A >> P
AutoAlign	---
Phase oversampling	0 %
FoV read	360 mm
FoV phase	90.6 %
Slice thickness	8.0 mm
TR	40.35 ms
TE	1.13 ms
Averages	1
Concatenations	11
Filter	Distortion Corr.(2D), Prescan Normalize, Image Filter
Coil elements	B1-5;SP3-6

Contrast - Common

TR	40.35 ms
TE	1.13 ms
Magn. preparation	None
Flip angle	52 deg
Fat suppr.	None
Wrap-up Magn.	Restore

Contrast - Dynamic

Averages	1
Averaging mode	Short term
Reconstruction	Magnitude
Measurements	1
Multiple series	Each slice

Resolution - Common

FoV read	360 mm
FoV phase	90.6 %
Slice thickness	8.0 mm
Base resolution	192
Phase resolution	100 %
Phase partial Fourier	Off
Trajectory	Cartesian
View sharing	Off
Interpolation	Off

Resolution - iPAT

PAT mode	GRAPPA
Accel. factor PE	3
Ref. lines PE	24
Reference scan mode	GRE/separate

Resolution - Filter Image

Image Filter	On
! Intensity	Medium
Edge Enhancement	2
Smoothing	2
Unfiltered images	Off
Distortion Corr.	On
Mode	2D
Unfiltered images	Off
Prescan Normalize	On
Unfiltered images	Off
Normalize	Off
B1 filter	Off

Resolution - Filter Rawdata

Raw filter	Off
Elliptical filter	Off
POCS	Off

Geometry - Common

Slice group	1
Slices	11
Dist. factor	25 %
Position	L54.9 A12.1 F10.8 mm
Orientation	S > C31.4 > T16.5
Phase enc. dir.	A >> P
FoV read	360 mm
FoV phase	90.6 %
Slice thickness	8.0 mm
TR	40.35 ms
Multi-slice mode	Sequential
Series	Base To Apex
Concatenations	11

Geometry - AutoAlign

Slice group	1
Position	L54.9 A12.1 F10.8 mm
Orientation	S > C31.4 > T16.5
Phase enc. dir.	A >> P
AutoAlign	---
Initial Position	L54.9 A12.1 F10.8
Phase	17.1 mm
Read	8.1 mm
Shift	54.0 mm
Initial Rotation	11.00 deg
Initial Orientation	S > C
S > C	31.4
> T	16.5

Geometry - Saturation

Fat suppr.	None
Wrap-up Magn.	Restore
Special sat.	None

Geometry - Navigator

System - Miscellaneous

Positioning mode	REF
Table position	H
Table position	0 mm
MSMA	S - C - T
Sagittal	R >> L
Coronal	A >> P
Transversal	F >> H
Coil Combine Mode	Adaptive Combine
Save uncombined	Off
Matrix Optimization	Off
Coil Focus	Flat
AutoAlign	---
Coil Select Mode	Default

System - Adjustments

B0 Shim mode	Tune up
Adjust with body coil	On
Confirm freq. adjustment	Off
Assume Dominant Fat	Off
Assume Silicone	Off
Adjustment Tolerance	Auto

System - Adjust Volume

Position	Isocenter
Orientation	Transversal
Rotation	0.00 deg
A >> P	263 mm
R >> L	350 mm
F >> H	350 mm
Reset	Off

System - Tx/Rx

Frequency 1H	63.678323 MHz
Correction factor	1
Gain	High
Img. Scale Cor.	1.000
Reset	Off
? Ref. amplitude 1H	0.000 V

Physio - Signal1

1st Signal/Mode	ECG/Retro
Average cycle	No Signal ms
Average cycle	No Signal ms
Calculated phases	25
TR	40.35 ms
Concatenations	11
Segments	15
Arrhythmia detection	None

Physio - Cardiac

Tagging	None
Magn. preparation	None
Fat suppr.	None
Dark blood	Off
FoV read	360 mm
FoV phase	90.6 %
Phase resolution	100 %
Cine	On
Trajectory	Cartesian
View sharing	Off
Dummy heartbeats	1

Physio - PACE

Resp. control	Breath-hold

Physio - PACE

Concatenations	11

Inline - Common

Subtract	Off
Measurements	1
StdDev	Off
Save original images	On

Inline - Cardiac

Inline Evaluation	Off
Magn. preparation	None
Contrasts	1
TE	1.13 ms
TR	40.35 ms
Save original images	On

Inline - MIP

MIP-Sag	Off
MIP-Cor	Off
MIP-Tra	Off
MIP-Time	Off
Save original images	On

Inline - Composing

Inline Composing	Off
Distortion Corr.	On
Mode	2D
Unfiltered images	Off

Sequence - Part 1

Introduction	Off
Dimension	2D
Reordering	Linear
Asymmetric echo	Weak
Contrasts	1
Optimization	Min. TE TR
Multi-slice mode	Sequential
Echo spacing	2.7 ms
Sequence type	Trufi
Bandwidth	930 Hz/Px

Sequence - Part 2

Define	Segments
Segments	15
Trufi delta freq.	0 Hz
RF pulse type	Fast
Gradient mode	Fast*
Excitation	Slice-sel.
Flip angle mode	Constant
Cine	On

Sequence - Assistant

Mode	Off
Allowed delay	0 s

\\USER\Cardiac Research Protocols\IV IRON\IV IRON4\VLS_ShMOLLI_192i_d11_nFilt
TA: 5.3 s PM: REF Voxel size: 0.9×0.9×8.0 mmPAT: 2 Rel. SNR: 1.00 : tfi

Properties

Prio recon	Off
Load images to viewer	On
Inline movie	Off
Auto store images	On
Load images to stamp segments	Off
Load images to graphic segments	On
Auto open inline display	Off
Auto close inline display	Off
Start measurement without further preparation	Off
Wait for user to start	Off
Start measurements	Single measurement

Routine

Slice group	1
Slices	1
Dist. factor	25 %
Position	Isocenter
Orientation	Transversal
Phase enc. dir.	A >> P
AutoAlign	---
Phase oversampling	0 %
FoV read	360 mm
FoV phase	75.0 %
Slice thickness	8.0 mm
TR	378.98 ms
TE	1.07 ms
Averages	1
Concatenations	1
Filter	Raw filter, Distortion Corr.(2D)
Coil elements	BO2;SP2,3

Contrast - Common

TR	378.98 ms
TE	1.07 ms
Magn. preparation	Non-sel. IR
TI	260 ms
Flip angle	35 deg
Fat suppr.	None
Wrap-up Magn.	None

Contrast - Dynamic

Averages	1
Averaging mode	Short term
Reconstruction	Magn./Phase
Measurements	1
Multiple series	Off

Resolution - Common

FoV read	360 mm
FoV phase	75.0 %
Slice thickness	8.0 mm
Base resolution	192
Phase resolution	100 %
Phase partial Fourier	6/8
Trajectory	Cartesian
Interpolation	On

Resolution - iPAT

PAT mode	GRAPPA
Accel. factor PE	2
Ref. lines PE	24
Reference scan mode	Integrated

Resolution - Filter Image

Image Filter	Off
Distortion Corr.	On
Mode	2D
Unfiltered images	Off
Prescan Normalize	Off
Normalize	Off
B1 filter	Off

Resolution - Filter Rawdata

Raw filter	On
Elliptical filter	Off
POCS	Off

Geometry - Common

Slice group	1
Slices	1
Dist. factor	25 %
Position	Isocenter
Orientation	Transversal
Phase enc. dir.	A >> P
FoV read	360 mm
FoV phase	75.0 %
Slice thickness	8.0 mm
TR	378.98 ms
Multi-slice mode	Sequential
Series	Interleaved
Concatenations	1

Geometry - AutoAlign

Slice group	1
Position	Isocenter
Orientation	Transversal
Phase enc. dir.	A >> P
AutoAlign	---
Initial Position	Isocenter
Phase	0.0 mm
Read	0.0 mm
Shift	0.0 mm
Initial Rotation	0.00 deg
Initial Orientation	Transversal

Geometry - Saturation

Fat suppr.	None
Wrap-up Magn.	None
Special sat.	None

Geometry - Navigator

System - Miscellaneous

Positioning mode	REF
Table position	H
Table position	0 mm
MSMA	S - C - T
Sagittal	R >> L

System - Miscellaneous

Coronal	A >> P
Transversal	F >> H
Coil Combine Mode	Adaptive Combine
Save uncombined	Off
Matrix Optimization	Off
Coil Focus	Flat
AutoAlign	---
Coil Select Mode	Default

System - Adjustments

B0 Shim mode	Cardiac
Adjust with body coil	On
Confirm freq. adjustment	Off
Assume Dominant Fat	Off
Assume Silicone	Off
Adjustment Tolerance	Auto

System - Adjust Volume

! Position	Isocenter
! Orientation	Transversal
! Rotation	0.00 deg
! A >> P	150 mm
! R >> L	150 mm
! F >> H	150 mm
Reset	Off

System - Tx/Rx

Frequency 1H	63.678323 MHz
Correction factor	1
Gain	High
Img. Scale Cor.	1.000
Reset	Off
? Ref. amplitude 1H	0.000 V

Physio - Signal1

1st Signal/Mode	ECG/Trigger
Average cycle	No Signal ms
Average cycle	No Signal ms
Captured cycle	-not set-
Acquisition window	591 ms
Trigger pulse	1
Trigger delay	212 ms
TR	378.98 ms
Concatenations	1
Segments	84
Phases	1
Adaptive Triggering	Off

Physio - Cardiac

Tagging	None
Magn. preparation	Non-sel. IR
TI	260 ms
Fat suppr.	None
Dark blood	Off
FoV read	360 mm
FoV phase	75.0 %
Phase resolution	100 %
Cine	Off
Trajectory	Cartesian
Dummy heartbeats	0
Motion Correction	None

Physio - PACE

Resp. control	Off
Concatenations	1

Sequence - Part 1

Introduction	Off
Dimension	2D
Reordering	Linear
Asymmetric echo	Weak
Contrasts	1
Optimization	Min. TE TR
Multi-slice mode	Sequential
Sequence type	Trufi
Bandwidth	898 Hz/Px

Sequence - Part 2

Define	Shots
Shots per slice	1
Segments	84
Trufi delta freq.	0 Hz
RF pulse type	Fast
Gradient mode	Fast
Excitation	Slice-sel.
Flip angle mode	Constant
Cine	Off

Sequence - Assistant

Mode	Off
Allowed delay	0 s

\\USER\Cardiac Research Protocols\IV IRON\IV IRON4\KLS_ShMOLLI_192i_d11_nFilt_FOV460

TA: 5.3 s PM: REF Voxel size: 1.2×1.2×8.0 mmPAT: 2 Rel. SNR: 1.00 : tfi

Properties

Prio recon	Off
Load images to viewer	On
Inline movie	Off
Auto store images	On
Load images to stamp segments	Off
Load images to graphic segments	On
Auto open inline display	Off
Auto close inline display	Off
Start measurement without further preparation	Off
Wait for user to start	Off
Start measurements	Single measurement

Routine

Slice group	1
Slices	1
Dist. factor	25 %
Position	Isocenter
Orientation	Coronal
Phase enc. dir.	R >> L
AutoAlign	---
Phase oversampling	0 %
FoV read	460 mm
FoV phase	75.0 %
Slice thickness	8.0 mm
TR	375.62 ms
TE	1.07 ms
Averages	1
Concatenations	1
Filter	Raw filter, Distortion Corr.(2D)
Coil elements	BO2;SP2,3

Contrast - Common

TR	375.62 ms
TE	1.07 ms
Magn. preparation	Non-sel. IR
TI	260 ms
Flip angle	35 deg
Fat suppr.	None
Wrap-up Magn.	None

Contrast - Dynamic

Averages	1
Averaging mode	Short term
Reconstruction	Magn./Phase
Measurements	1
Multiple series	Off

Resolution - Common

FoV read	460 mm
FoV phase	75.0 %
Slice thickness	8.0 mm
Base resolution	192
Phase resolution	100 %
Phase partial Fourier	6/8
Trajectory	Cartesian
Interpolation	On

Resolution - iPAT

PAT mode	GRAPPA
Accel. factor PE	2
Ref. lines PE	24
Reference scan mode	Integrated

Resolution - Filter Image

Image Filter	Off
Distortion Corr.	On
Mode	2D
Unfiltered images	Off
Prescan Normalize	Off
Normalize	Off
B1 filter	Off

Resolution - Filter Rawdata

Raw filter	On
Elliptical filter	Off
POCS	Off

Geometry - Common

Slice group	1
Slices	1
Dist. factor	25 %
Position	Isocenter
Orientation	Coronal
Phase enc. dir.	R >> L
FoV read	460 mm
FoV phase	75.0 %
Slice thickness	8.0 mm
TR	375.62 ms
Multi-slice mode	Sequential
Series	Interleaved
Concatenations	1

Geometry - AutoAlign

Slice group	1
Position	Isocenter
Orientation	Coronal
Phase enc. dir.	R >> L
AutoAlign	---
Initial Position	Isocenter
Phase	0.0 mm
Read	0.0 mm
Shift	0.0 mm
Initial Rotation	0.00 deg
Initial Orientation	Coronal

Geometry - Saturation

Fat suppr.	None
Wrap-up Magn.	None
Special sat.	None

Geometry - Navigator

System - Miscellaneous

Positioning mode	REF
Table position	H
Table position	0 mm
MSMA	S - C - T
Sagittal	R >> L

System - Miscellaneous

Coronal	A >> P
Transversal	F >> H
Coil Combine Mode	Adaptive Combine
Save uncombined	Off
Matrix Optimization	Off
Coil Focus	Flat
AutoAlign	---
Coil Select Mode	Default

System - Adjustments

B0 Shim mode	Cardiac
Adjust with body coil	On
Confirm freq. adjustment	Off
Assume Dominant Fat	Off
Assume Silicone	Off
Adjustment Tolerance	Auto

System - Adjust Volume

! Position	Isocenter
! Orientation	Transversal
! Rotation	0.00 deg
! A >> P	150 mm
! R >> L	150 mm
! F >> H	150 mm
Reset	Off

System - Tx/Rx

Frequency 1H	63.678323 MHz
Correction factor	1
Gain	High
Img. Scale Cor.	1.000
Reset	Off
? Ref. amplitude 1H	0.000 V

Physio - Signal1

1st Signal/Mode	ECG/Trigger
Average cycle	No Signal ms
Average cycle	No Signal ms
Captured cycle	-not set-
Acquisition window	591 ms
Trigger pulse	1
Trigger delay	215 ms
TR	375.62 ms
Concatenations	1
Segments	84
Phases	1
Adaptive Triggering	Off

Physio - Cardiac

Tagging	None
Magn. preparation	Non-sel. IR
TI	260 ms
Fat suppr.	None
Dark blood	Off
FoV read	460 mm
FoV phase	75.0 %
Phase resolution	100 %
Cine	Off
Trajectory	Cartesian
Dummy heartbeats	0
Motion Correction	None

Physio - PACE

Resp. control	Off

Physio - PACE

Concatenations	1

Sequence - Part 1

Introduction	Off
Dimension	2D
Reordering	Linear
Asymmetric echo	Weak
Contrasts	1
Optimization	Min. TE TR
Multi-slice mode	Sequential
Sequence type	Trufi
Bandwidth	898 Hz/Px

Sequence - Part 2

Define	Shots
Shots per slice	1
Segments	84
Trufi delta freq.	0 Hz
RF pulse type	Fast
Gradient mode	Fast
Excitation	Slice-sel.
Flip angle mode	Constant
Cine	Off

Sequence - Assistant

Mode	Off
Allowed delay	0 s

\\USER\Cardiac Research Protocols\IV IRON\IV IRON4\VLST2Map_TrueFISP

TA: 5.3 s PM: REF Voxel size: 1.9×1.9×8.0 mmPAT: 2 Rel. SNR: 1.00 : tfi

Properties

Prio recon	Off
Load images to viewer	On
Inline movie	Off
Auto store images	On
Load images to stamp segments	Off
Load images to graphic segments	On
Auto open inline display	Off
Auto close inline display	Off
Start measurement without further preparation	Off
Wait for user to start	On
Start measurements	Single measurement

Routine

Slice group	1
Slices	1
Dist. factor	20 %
Position	Isocenter
Orientation	Transversal
Phase enc. dir.	A >> P
AutoAlign	---
Phase oversampling	0 %
FoV read	360 mm
FoV phase	75.0 %
Slice thickness	8.0 mm
TR	185.80 ms
TE	1.07 ms
Averages	1
Concatenations	1
Filter	Distortion Corr.(2D)
Coil elements	BO1-3;SP1-3

Contrast - Common

TR	185.80 ms
TE	1.07 ms
Magn. preparation	T2 prep. adiab.
T2 prep. duration 1	0 ms
T2 prep. duration 2	25 ms
T2 prep. duration 3	55 ms
Flip angle	70 deg
Fat suppr.	None
Wrap-up Magn.	None

Contrast - Dynamic

Averages	1
Averaging mode	Short term
Reconstruction	Magnitude
Measurements	1
Multiple series	Off

Resolution - Common

FoV read	360 mm
FoV phase	75.0 %
Slice thickness	8.0 mm
Base resolution	192
Phase resolution	75 %
Phase partial Fourier	6/8
Trajectory	Cartesian
Interpolation	Off

Resolution - iPAT

PAT mode	GRAPPA
Accel. factor PE	2
Ref. lines PE	36
Reference scan mode	GRE/separate

Resolution - Filter Image

Image Filter	Off
Distortion Corr.	On
Mode	2D
Unfiltered images	Off
Prescan Normalize	Off
Normalize	Off
B1 filter	Off

Resolution - Filter Rawdata

Raw filter	Off
Elliptical filter	Off
POCS	Off

Geometry - Common

Slice group	1
Slices	1
Dist. factor	20 %
Position	Isocenter
Orientation	Transversal
Phase enc. dir.	A >> P
FoV read	360 mm
FoV phase	75.0 %
Slice thickness	8.0 mm
TR	185.80 ms
Multi-slice mode	Sequential
Series	Base To Apex
Concatenations	1

Geometry - AutoAlign

Slice group	1
Position	Isocenter
Orientation	Transversal
Phase enc. dir.	A >> P
AutoAlign	---
Initial Position	Isocenter
Phase	0.0 mm
Read	0.0 mm
Shift	0.0 mm
Initial Rotation	0.00 deg
Initial Orientation	Transversal

Geometry - Saturation

Fat suppr.	None
Wrap-up Magn.	None
Special sat.	None

Geometry - Navigator

System - Miscellaneous

Positioning mode	REF
Table position	H
Table position	0 mm
MSMA	S - C - T
Sagittal	R >> L

System - Miscellaneous

Coronal	A >> P
Transversal	F >> H
Coil Combine Mode	Adaptive Combine
Save uncombined	Off
Matrix Optimization	Off
Coil Focus	Flat
AutoAlign	---
Coil Select Mode	Default

System - Adjustments

B0 Shim mode	Tune up
Adjust with body coil	Off
Confirm freq. adjustment	Off
Assume Dominant Fat	Off
Assume Silicone	Off
Adjustment Tolerance	Auto

System - Adjust Volume

Position	Isocenter
Orientation	Transversal
Rotation	0.00 deg
A >> P	263 mm
R >> L	350 mm
F >> H	350 mm
Reset	Off

System - Tx/Rx

Frequency 1H	63.678323 MHz
Correction factor	1
Gain	High
Img. Scale Cor.	1.000
Reset	Off
? Ref. amplitude 1H	0.000 V

Physio - Signal1

1st Signal/Mode	ECG/Trigger
Average cycle	No Signal ms
Average cycle	No Signal ms
Captured cycle	-not set-
Acquisition window	591 ms
Trigger pulse	1
Trigger delay	405 ms
TR	185.80 ms
Concatenations	1
Segments	54
Phases	1
Adaptive Triggering	Off

Physio - Cardiac

Tagging	None
Magn. preparation	T2 prep. adiab.
T2 prep. duration 1	0 ms
T2 prep. duration 2	25 ms
T2 prep. duration 3	55 ms
Fat suppr.	None
Dark blood	Off
FoV read	360 mm
FoV phase	75.0 %
Phase resolution	75 %
Cine	Off
Trajectory	Cartesian
Dummy heartbeats	0
Motion Correction	Standard

Physio - PACE

Resp. control	Breath-hold
Concatenations	1

Sequence - Part 1

Introduction	Off
Dimension	2D
Reordering	Linear
Asymmetric echo	Weak
Contrasts	1
Optimization	Min. TE TR
Multi-slice mode	Sequential
Sequence type	Trufi
Bandwidth	1184 Hz/Px

Sequence - Part 2

Define	Shots
Shots per slice	1
Segments	54
Trufi delta freq.	0 Hz
RF pulse type	Fast
Gradient mode	Fast
Excitation	Slice-sel.
Flip angle mode	Constant
Cine	Off

Sequence - Assistant

Mode	Off
Allowed delay	0 s

\\USER\Cardiac Research Protocols\IV IRON\IV IRON4\VLSShMOLLI_C2P_m
TA: 5.3 s PM: FIX Voxel size: 0.9×0.9×8.0 mmPAT: 2 Rel. SNR: 1.00 : tfi

Properties

Prio recon	Off
Load images to viewer	On
Inline movie	Off
Auto store images	On
Load images to stamp segments	Off
Load images to graphic segments	On
Auto open inline display	Off
Auto close inline display	Off
Start measurement without further preparation	On
Wait for user to start	Off
Start measurements	Single measurement

Routine

Slice group	1
Slices	1
Dist. factor	25 %
Position	Isocenter
Orientation	Transversal
Phase enc. dir.	A >> P
AutoAlign	---
Phase oversampling	0 %
FoV read	360 mm
FoV phase	75.0 %
Slice thickness	8.0 mm
TR	378.98 ms
TE	1.07 ms
Averages	1
Concatenations	1
Filter	Raw filter, Distortion Corr.(2D)
Coil elements	BO1-3;SP1-3

Contrast - Common

TR	378.98 ms
TE	1.07 ms
Magn. preparation	Non-sel. IR
TI	260 ms
Flip angle	35 deg
Fat suppr.	None
Wrap-up Magn.	None

Contrast - Dynamic

Averages	1
Averaging mode	Short term
Reconstruction	Magn./Phase
Measurements	1
Multiple series	Off

Resolution - Common

FoV read	360 mm
FoV phase	75.0 %
Slice thickness	8.0 mm
Base resolution	192
Phase resolution	100 %
Phase partial Fourier	6/8
Trajectory	Cartesian
Interpolation	On

Resolution - iPAT

PAT mode	GRAPPA
Accel. factor PE	2
Ref. lines PE	24
Reference scan mode	Integrated

Resolution - Filter Image

Image Filter	Off
Distortion Corr.	On
Mode	2D
Unfiltered images	Off
Prescan Normalize	Off
Normalize	Off
B1 filter	Off

Resolution - Filter Rawdata

Raw filter	On
Elliptical filter	Off
POCS	Off

Geometry - Common

Slice group	1
Slices	1
Dist. factor	25 %
Position	Isocenter
Orientation	Transversal
Phase enc. dir.	A >> P
FoV read	360 mm
FoV phase	75.0 %
Slice thickness	8.0 mm
TR	378.98 ms
Multi-slice mode	Sequential
Series	Interleaved
Concatenations	1

Geometry - AutoAlign

Slice group	1
Position	Isocenter
Orientation	Transversal
Phase enc. dir.	A >> P
AutoAlign	---
Initial Position	Isocenter
Phase	0.0 mm
Read	0.0 mm
Shift	0.0 mm
Initial Rotation	0.00 deg
Initial Orientation	Transversal

Geometry - Saturation

Fat suppr.	None
Wrap-up Magn.	None
Special sat.	None

Geometry - Navigator

System - Miscellaneous

Positioning mode	FIX
Table position	H
Table position	0 mm
MSMA	S - C - T
Sagittal	R >> L

System - Miscellaneous

Coronal	A >> P
Transversal	F >> H
Coil Combine Mode	Adaptive Combine
Save uncombined	Off
Matrix Optimization	Off
Coil Focus	Flat
AutoAlign	---
Coil Select Mode	Default

System - Adjustments

B0 Shim mode	Tune up
Adjust with body coil	On
Confirm freq. adjustment	Off
Assume Dominant Fat	Off
Assume Silicone	Off
Adjustment Tolerance	Auto

System - Adjust Volume

! Position	Isocenter
! Orientation	Transversal
! Rotation	0.00 deg
! A >> P	263 mm
! R >> L	350 mm
! F >> H	350 mm
Reset	Off

System - Tx/Rx

Frequency 1H	63.678323 MHz
Correction factor	1
Gain	High
Img. Scale Cor.	1.000
Reset	Off
? Ref. amplitude 1H	0.000 V

Physio - Signal1

1st Signal/Mode	ECG/Trigger
Average cycle	No Signal ms
Average cycle	No Signal ms
Captured cycle	-not set-
Acquisition window	591 ms
Trigger pulse	1
Trigger delay	212 ms
TR	378.98 ms
Concatenations	1
Segments	84
Phases	1
Adaptive Triggering	Off

Physio - Cardiac

Tagging	None
Magn. preparation	Non-sel. IR
TI	260 ms
Fat suppr.	None
Dark blood	Off
FoV read	360 mm
FoV phase	75.0 %
Phase resolution	100 %
Cine	Off
Trajectory	Cartesian
Dummy heartbeats	0
Motion Correction	Standard

Physio - PACE

Resp. control	Off

Physio - PACE

Concatenations	1

Sequence - Part 1

Introduction	Off
Dimension	2D
Reordering	Linear
Asymmetric echo	Weak
Contrasts	1
Optimization	Min. TE TR
Multi-slice mode	Sequential
Sequence type	Trufi
Bandwidth	898 Hz/Px

Sequence - Part 2

Define	Shots
Shots per slice	1
Segments	84
Trufi delta freq.	0 Hz
RF pulse type	Fast
Gradient mode	Fast
Excitation	Slice-sel.
Flip angle mode	Constant
Cine	Off

Sequence - Assistant

Mode	Off
Allowed delay	0 s

\\USER\Cardiac Research Protocols\IV IRON\IV IRON4\VLST2StarMap_8echo_heart
TA: 9.4 s PM: FIX Voxel size: 1.4×1.4×8.0 mmPAT: 2 Rel. SNR: 1.00 : fl_r

Properties

Prio recon	Off
Load images to viewer	On
Inline movie	Off
Auto store images	On
Load images to stamp segments	Off
Load images to graphic segments	On
Auto open inline display	Off
Auto close inline display	Off
Start measurement without further preparation	On
Wait for user to start	Off
Start measurements	Single measurement

Routine

Slice group	1
Slices	1
Dist. factor	20 %
Position	Isocenter
Orientation	Transversal
Phase enc. dir.	A >> P
AutoAlign	---
Phase oversampling	0 %
FoV read	360 mm
FoV phase	75.0 %
Slice thickness	8.0 mm
TR	590.00 ms
TE 1	2.11 ms
TE 2	4.25 ms
TE 3	6.39 ms
TE 4	8.53 ms
TE 5	10.67 ms
TE 6	12.81 ms
TE 7	14.95 ms
TE 8	17.09 ms
Averages	1
Concatenations	1
Filter	Distortion Corr.(2D)
Coil elements	BO1-3;SP1-3

Contrast - Common

TR	590.00 ms
TE 1	2.11 ms
TE 2	4.25 ms
TE 3	6.39 ms
TE 4	8.53 ms
TE 5	10.67 ms
TE 6	12.81 ms
TE 7	14.95 ms
TE 8	17.09 ms
Flip angle	20 deg
Fat suppr.	Fat sat.
Wrap-up Magn.	None

Contrast - Dynamic

Averages	1
Averaging mode	Short term
Reconstruction	Magnitude
Measurements	1
Multiple series	Off

Resolution - Common

FoV read	360 mm
FoV phase	75.0 %
Slice thickness	8.0 mm
Base resolution	256
Phase resolution	60 %
Phase partial Fourier	Off
Trajectory	Cartesian
Interpolation	Off

Resolution - iPAT

PAT mode	GRAPPA
Accel. factor PE	2
Ref. lines PE	24
Reference scan mode	Integrated

Resolution - Filter Image

Image Filter	Off
Distortion Corr.	On
Mode	2D
Unfiltered images	Off
Prescan Normalize	Off
Normalize	Off
B1 filter	Off

Resolution - Filter Rawdata

Raw filter	Off
Elliptical filter	Off
POCS	Off

Geometry - Common

Slice group	1
Slices	1
Dist. factor	20 %
Position	Isocenter
Orientation	Transversal
Phase enc. dir.	A >> P
FoV read	360 mm
FoV phase	75.0 %
Slice thickness	8.0 mm
TR	590.00 ms
Multi-slice mode	Sequential
Series	Base To Apex
Concatenations	1

Geometry - AutoAlign

Slice group	1
Position	Isocenter
Orientation	Transversal
Phase enc. dir.	A >> P
AutoAlign	---
Initial Position	Isocenter
Phase	0.0 mm
Read	0.0 mm
Shift	0.0 mm
Initial Rotation	0.00 deg
Initial Orientation	Transversal

Geometry - Saturation

Fat suppr.	Fat sat.
Wrap-up Magn.	None

Geometry - Saturation

Special sat.	None

Geometry - Navigator

System - Miscellaneous

Positioning mode	FIX
Table position	H
Table position	0 mm
MSMA	S - C - T
Sagittal	R >> L
Coronal	A >> P
Transversal	F >> H
Coil Combine Mode	Adaptive Combine
Save uncombined	Off
Matrix Optimization	Off
Coil Focus	Flat
AutoAlign	---
Coil Select Mode	Default

System - Adjustments

B0 Shim mode	Tune up
Adjust with body coil	Off
Confirm freq. adjustment	Off
Assume Dominant Fat	Off
Assume Silicone	Off
Adjustment Tolerance	Auto

System - Adjust Volume

! Position	Isocenter
! Orientation	Transversal
! Rotation	0.00 deg
! A >> P	263 mm
! R >> L	350 mm
! F >> H	350 mm
Reset	Off

System - Tx/Rx

Frequency 1H	63.678323 MHz
Correction factor	1
Gain	High
Img. Scale Cor.	1.000
Reset	Off
? Ref. amplitude 1H	0.000 V

Physio - Signal1

1st Signal/Mode	ECG/Trigger
Average cycle	No Signal ms
Average cycle	No Signal ms
Captured cycle	-not set-
Acquisition window	590 ms
Trigger pulse	2
Trigger delay	0 ms
TR	590.00 ms
Concatenations	1
Segments	9
Phases	1
Adaptive Triggering	Off

Physio - Cardiac

Tagging	None
Fat suppr.	Fat sat.
Dark blood	On
Dark blood thickness	200 %

Physio - Cardiac

FoV read	360 mm
FoV phase	75.0 %
Phase resolution	60 %
Cine	Off
Trajectory	Cartesian
Dummy heartbeats	0

Physio - PACE

Resp. control	Breath-hold
Concatenations	1

Inline - Common

Subtract	Off
Measurements	1
StdDev	Off
Save original images	On

Inline - Cardiac

Inline Evaluation	T2* map
Contrasts	8
TE 1	2.11 ms
TE 2	4.25 ms
TE 3	6.39 ms
TE 4	8.53 ms
TE 5	10.67 ms
TE 6	12.81 ms
TE 7	14.95 ms
TE 8	17.09 ms
TR	590.00 ms
Save original images	On

Inline - MIP

MIP-Sag	Off
MIP-Cor	Off
MIP-Tra	Off
MIP-Time	Off
Save original images	On

Inline - Composing

Inline Composing	Off
Distortion Corr.	On
Mode	2D
Unfiltered images	Off

Sequence - Part 1

Introduction	Off
Dimension	2D
Reordering	Linear
Asymmetric echo	Weak
Contrasts	8
Flow comp. 1	Yes
Readout mode	Monopolar
Optimization	Min. TE
Multi-slice mode	Sequential
Echo spacing	19.1 ms
Sequence type	Gre
Bandwidth 1	814 Hz/Px
Bandwidth 2	814 Hz/Px
Bandwidth 3	814 Hz/Px
Bandwidth 4	814 Hz/Px
Bandwidth 5	814 Hz/Px
Bandwidth 6	814 Hz/Px
Bandwidth 7	814 Hz/Px
Bandwidth 8	814 Hz/Px

Sequence - Part 2

Define	Segments
Segments	9
RF pulse type	Fast
Gradient mode	Fast
Excitation	Slice-sel.
Flip angle mode	Constant
RF spoiling	On
Phase Enc. Rewinder	On
Cine	Off

Sequence - Assistant

Mode	Off
Allowed delay	0 s

\\USER\Cardiac Research Protocols\IV IRON\IV IRON4\VLSFB_MOCO_gt_T2star_DB_8e_128 FS_HiFE

TA: 9.4 s PM: FIX Voxel size: 2.8×2.8×8.0 mmPAT: 4 Rel. SNR: 1.00 : tfl

Properties

Prio recon	Off
Load images to viewer	On
Inline movie	Off
Auto store images	On
Load images to stamp segments	Off
Load images to graphic segments	On
Auto open inline display	Off
Auto close inline display	Off
Start measurement without further preparation	On
Wait for user to start	Off
Start measurements	Single measurement

Routine

Slice group	1
Slices	1
Dist. factor	20 %
Position	Isocenter
Orientation	Transversal
Phase enc. dir.	A >> P
AutoAlign	---
Phase oversampling	0 %
FoV read	360 mm
FoV phase	75.0 %
Slice thickness	8.0 mm
TR	590.00 ms
TE 1	1.07 ms
TE 2	2.58 ms
TE 3	4.09 ms
TE 4	5.6 ms
TE 5	7.11 ms
TE 6	8.62 ms
TE 7	10.13 ms
TE 8	11.64 ms
Averages	1
Concatenations	1
Filter	Distortion Corr.(2D)
Coil elements	BO1-3;SP1-3

Contrast - Common

TR	590.00 ms
TE 1	1.07 ms
TE 2	2.58 ms
TE 3	4.09 ms
TE 4	5.6 ms
TE 5	7.11 ms
TE 6	8.62 ms
TE 7	10.13 ms
TE 8	11.64 ms
Magn. preparation	None
Flip angle	18 deg
Fat suppr.	Fat sat.
Wrap-up Magn.	None

Contrast - Dynamic

Averages	1
Averaging mode	Long term
Reconstruction	Magnitude
Measurements	16

Contrast - Dynamic

Pause after meas. 1	0.0 s
Pause after meas. 2	0.0 s
Pause after meas. 3	0.0 s
Pause after meas. 4	0.0 s
Pause after meas. 5	0.0 s
Pause after meas. 6	0.0 s
Pause after meas. 7	0.0 s
Pause after meas. 8	0.0 s
Pause after meas. 9	0.0 s
Pause after meas. 10	0.0 s
Pause after meas. 11	0.0 s
Pause after meas. 12	0.0 s
Pause after meas. 13	0.0 s
Pause after meas. 14	0.0 s
Pause after meas. 15	0.0 s
Multiple series	Off

Resolution - Common

FoV read	360 mm
FoV phase	75.0 %
Slice thickness	8.0 mm
Base resolution	128
Phase resolution	100 %
Phase partial Fourier	Off
Trajectory	Cartesian
Interpolation	Off

Resolution - iPAT

PAT mode	GRAPPA
Accel. factor PE	4
Reference scan mode	T-PAT

Resolution - Filter Image

Image Filter	Off
Distortion Corr.	On
Mode	2D
Unfiltered images	Off
Prescan Normalize	Off
Normalize	Off
B1 filter	Off

Resolution - Filter Rawdata

Raw filter	Off
Elliptical filter	Off
POCS	Off

Geometry - Common

Slice group	1
Slices	1
Dist. factor	20 %
Position	Isocenter
Orientation	Transversal
Phase enc. dir.	A >> P
FoV read	360 mm
FoV phase	75.0 %
Slice thickness	8.0 mm
TR	590.00 ms
Multi-slice mode	Single shot
Series	Interleaved

Geometry - Common

Concatenations	1

Geometry - AutoAlign

Slice group	1
Position	Isocenter
Orientation	Transversal
Phase enc. dir.	A >> P
AutoAlign	---
Initial Position	Isocenter
Phase	0.0 mm
Read	0.0 mm
Shift	0.0 mm
Initial Rotation	0.00 deg
Initial Orientation	Transversal

Geometry - Saturation

Fat suppr.	Fat sat.
Wrap-up Magn.	None
Special sat.	None

Geometry - Navigator

System - Miscellaneous

Positioning mode	FIX
Table position	H
Table position	0 mm
MSMA	S - C - T
Sagittal	R >> L
Coronal	A >> P
Transversal	F >> H
Coil Combine Mode	Adaptive Combine
Save uncombined	Off
Matrix Optimization	Off
Coil Focus	Flat
AutoAlign	---
Coil Select Mode	Default

System - Adjustments

B0 Shim mode	Tune up
Adjust with body coil	On
Confirm freq. adjustment	Off
Assume Dominant Fat	Off
Assume Silicone	Off
Adjustment Tolerance	Auto

System - Adjust Volume

! Position	Isocenter
! Orientation	Transversal
! Rotation	0.00 deg
! A >> P	263 mm
! R >> L	350 mm
! F >> H	350 mm
Reset	Off

System - Tx/Rx

Frequency 1H	63.678323 MHz
Correction factor	1
Gain	High
Img. Scale Cor.	1.000
Reset	Off
? Ref. amplitude 1H	0.000 V

Physio - Signal1

1st Signal/Mode	ECG/Trigger
Average cycle	No Signal ms
Average cycle	No Signal ms
Captured cycle	-not set-
Acquisition window	590 ms
Trigger pulse	1
Trigger delay	0 ms
TR	590.00 ms
Concatenations	1
Segments	24
Phases	1
Adaptive Triggering	Off

Physio - Cardiac

Tagging	None
Magn. preparation	None
Fat suppr.	Fat sat.
Dark blood	On
Dark blood thickness	300 %
FoV read	360 mm
FoV phase	75.0 %
Phase resolution	100 %
Cine	Off
Trajectory	Cartesian
Dummy heartbeats	0

Physio - PACE

Resp. control	Off
Concatenations	1

Inline - Common

Subtract	Off
Measurements	16
StdDev	Off
Save original images	On

Inline - Cardiac

Inline Evaluation	Off
Magn. preparation	None
Contrasts	8
TE 1	1.07 ms
TE 2	2.58 ms
TE 3	4.09 ms
TE 4	5.6 ms
TE 5	7.11 ms
TE 6	8.62 ms
TE 7	10.13 ms
TE 8	11.64 ms
TR	590.00 ms
Save original images	On

Inline - MIP

MIP-Sag	Off
MIP-Cor	Off
MIP-Tra	Off
MIP-Time	Off
Save original images	On

Inline - Composing

Inline Composing	Off
Distortion Corr.	On
Mode	2D
Unfiltered images	Off

Sequence - Part 1

Introduction	Off
Dimension	2D
Reordering	Linear
Asymmetric echo	Off
Contrasts	8
Flow comp. 1	No
Readout mode	Monopolar
Optimization	Min. TE
Multi-slice mode	Single shot
Echo spacing	12.6 ms
Sequence type	Gre
Bandwidth 1	1502 Hz/Px
Bandwidth 2	1502 Hz/Px
Bandwidth 3	1502 Hz/Px
Bandwidth 4	1502 Hz/Px
Bandwidth 5	1502 Hz/Px
Bandwidth 6	1502 Hz/Px
Bandwidth 7	1502 Hz/Px
Bandwidth 8	1502 Hz/Px

Sequence - Part 2

Define	Shots
Shots per slice	1
Segments	24
RF pulse type	Fast
Gradient mode	Fast
Excitation	Slice-sel.
Flip angle mode	Constant
RF spoiling	On
Phase Enc. Rewinder	On
Cine	Off

Sequence - Special

FatWater Separation	On
Multi-echo Images	Off
In-Opp Phase Images	Off
Frequency Map	Off
T2* Map	Off
Motion Correction	Off
MoCo Averaging Mode	Complex MoCo
MoCo Images Only?	Off
No. of Interleaves	0

Sequence - Assistant

Mode	Off
Allowed delay	0 s

\\USER\Cardiac Research Protocols\IV IRON\IV IRON4\VLSFB_MOCO_gt_T2star_DB_8e_160 FS_LowNFE

TA: 9.4 s PM: FIX Voxel size: 2.3×2.3×8.0 mmPAT: 4 Rel. SNR: 1.00 : tfl

Properties

Prio recon	Off
Load images to viewer	On
Inline movie	Off
Auto store images	On
Load images to stamp segments	Off
Load images to graphic segments	On
Auto open inline display	Off
Auto close inline display	Off
Start measurement without further preparation	On
Wait for user to start	Off
Start measurements	Single measurement

Routine

Slice group	1
Slices	1
Dist. factor	20 %
Position	Isocenter
Orientation	Transversal
Phase enc. dir.	A >> P
AutoAlign	---
Phase oversampling	0 %
FoV read	360 mm
FoV phase	75.0 %
Slice thickness	8.0 mm
TR	590.00 ms
TE 1	1.22 ms
TE 2	3.05 ms
TE 3	4.88 ms
TE 4	6.71 ms
TE 5	8.54 ms
TE 6	10.37 ms
TE 7	12.2 ms
TE 8	14.03 ms
Averages	1
Concatenations	1
Filter	Distortion Corr.(2D)
Coil elements	BO1-3;SP1-3

Contrast - Common

TR	590.00 ms
TE 1	1.22 ms
TE 2	3.05 ms
TE 3	4.88 ms
TE 4	6.71 ms
TE 5	8.54 ms
TE 6	10.37 ms
TE 7	12.2 ms
TE 8	14.03 ms
Magn. preparation	None
Flip angle	18 deg
Fat suppr.	Fat sat.
Wrap-up Magn.	None

Contrast - Dynamic

Averages	1
Averaging mode	Long term
Reconstruction	Magnitude
Measurements	16

Contrast - Dynamic

Pause after meas. 1	0.0 s
Pause after meas. 2	0.0 s
Pause after meas. 3	0.0 s
Pause after meas. 4	0.0 s
Pause after meas. 5	0.0 s
Pause after meas. 6	0.0 s
Pause after meas. 7	0.0 s
Pause after meas. 8	0.0 s
Pause after meas. 9	0.0 s
Pause after meas. 10	0.0 s
Pause after meas. 11	0.0 s
Pause after meas. 12	0.0 s
Pause after meas. 13	0.0 s
Pause after meas. 14	0.0 s
Pause after meas. 15	0.0 s
Multiple series	Off

Resolution - Common

FoV read	360 mm
FoV phase	75.0 %
Slice thickness	8.0 mm
Base resolution	160
Phase resolution	87 %
Phase partial Fourier	Off
Trajectory	Cartesian
Interpolation	Off

Resolution - iPAT

PAT mode	GRAPPA
Accel. factor PE	4
Reference scan mode	T-PAT

Resolution - Filter Image

Image Filter	Off
Distortion Corr.	On
Mode	2D
Unfiltered images	Off
Prescan Normalize	Off
Normalize	Off
B1 filter	Off

Resolution - Filter Rawdata

Raw filter	Off
Elliptical filter	Off
POCS	Off

Geometry - Common

Slice group	1
Slices	1
Dist. factor	20 %
Position	Isocenter
Orientation	Transversal
Phase enc. dir.	A >> P
FoV read	360 mm
FoV phase	75.0 %
Slice thickness	8.0 mm
TR	590.00 ms
Multi-slice mode	Single shot
Series	Interleaved

Geometry - Common

Concatenations	1

Geometry - AutoAlign

Slice group	1
Position	Isocenter
Orientation	Transversal
Phase enc. dir.	A >> P
AutoAlign	---
Initial Position	Isocenter
Phase	0.0 mm
Read	0.0 mm
Shift	0.0 mm
Initial Rotation	0.00 deg
Initial Orientation	Transversal

Geometry - Saturation

Fat suppr.	Fat sat.
Wrap-up Magn.	None
Special sat.	None

Geometry - Navigator

System - Miscellaneous

Positioning mode	FIX
Table position	H
Table position	0 mm
MSMA	S - C - T
Sagittal	R >> L
Coronal	A >> P
Transversal	F >> H
Coil Combine Mode	Adaptive Combine
Save uncombined	Off
Matrix Optimization	Off
Coil Focus	Flat
AutoAlign	---
Coil Select Mode	Default

System - Adjustments

B0 Shim mode	Tune up
Adjust with body coil	On
Confirm freq. adjustment	Off
Assume Dominant Fat	Off
Assume Silicone	Off
Adjustment Tolerance	Auto

System - Adjust Volume

! Position	Isocenter
! Orientation	Transversal
! Rotation	0.00 deg
! A >> P	263 mm
! R >> L	350 mm
! F >> H	350 mm
Reset	Off

System - Tx/Rx

Frequency 1H	63.678323 MHz
Correction factor	1
Gain	High
Img. Scale Cor.	1.000
Reset	Off
? Ref. amplitude 1H	0.000 V

Physio - Signal1

1st Signal/Mode	ECG/Trigger
Average cycle	No Signal ms
Average cycle	No Signal ms
Captured cycle	-not set-
Acquisition window	590 ms
Trigger pulse	1
Trigger delay	0 ms
TR	590.00 ms
Concatenations	1
Segments	26
Phases	1
Adaptive Triggering	Off

Physio - Cardiac

Tagging	None
Magn. preparation	None
Fat suppr.	Fat sat.
Dark blood	On
Dark blood thickness	300 %
FoV read	360 mm
FoV phase	75.0 %
Phase resolution	87 %
Cine	Off
Trajectory	Cartesian
Dummy heartbeats	0

Physio - PACE

Resp. control	Off
Concatenations	1

Inline - Common

Subtract	Off
Measurements	16
StdDev	Off
Save original images	On

Inline - Cardiac

Inline Evaluation	Off
Magn. preparation	None
Contrasts	8
TE 1	1.22 ms
TE 2	3.05 ms
TE 3	4.88 ms
TE 4	6.71 ms
TE 5	8.54 ms
TE 6	10.37 ms
TE 7	12.2 ms
TE 8	14.03 ms
TR	590.00 ms
Save original images	On

Inline - MIP

MIP-Sag	Off
MIP-Cor	Off
MIP-Tra	Off
MIP-Time	Off
Save original images	On

Inline - Composing

Inline Composing	Off
Distortion Corr.	On
Mode	2D
Unfiltered images	Off

Sequence - Part 1

Introduction	Off
Dimension	2D
Reordering	Linear
Asymmetric echo	Off
Contrasts	8
Flow comp. 1	No
Readout mode	Monopolar
Optimization	Min. TE
Multi-slice mode	Single shot
Echo spacing	15.1 ms
Sequence type	Gre
Bandwidth 1	1078 Hz/Px
Bandwidth 2	1078 Hz/Px
Bandwidth 3	1078 Hz/Px
Bandwidth 4	1078 Hz/Px
Bandwidth 5	1078 Hz/Px
Bandwidth 6	1078 Hz/Px
Bandwidth 7	1078 Hz/Px
Bandwidth 8	1078 Hz/Px

Sequence - Part 2

Define	Shots
Shots per slice	1
Segments	26
RF pulse type	Fast
Gradient mode	Fast
Excitation	Slice-sel.
Flip angle mode	Constant
RF spoiling	On
Phase Enc. Rewinder	On
Cine	Off

Sequence - Special

FatWater Separation	On
Multi-echo Images	Off
In-Opp Phase Images	Off
Frequency Map	Off
T2* Map	Off
Motion Correction	Off
MoCo Averaging Mode	Complex MoCo
MoCo Images Only?	Off
No. of Interleaves	0

Sequence - Assistant

Mode	Off
Allowed delay	0 s

\\USER\Cardiac Research Protocols\IV IRON\IV IRON4\VLSnonBH_T2StarMap_12echo_liver
TA: 0:23 PM: FIX Voxel size: 2.8×2.8×8.0 mmPAT: Off Rel. SNR: 1.00 : fl

Properties

Prio recon	Off
Load images to viewer	On
Inline movie	Off
Auto store images	On
Load images to stamp segments	Off
Load images to graphic segments	On
Auto open inline display	Off
Auto close inline display	Off
Start measurement without further preparation	On
Wait for user to start	Off
Start measurements	Single measurement

Routine

Slice group	1
Slices	1
Dist. factor	20 %
Position	Isocenter
Orientation	Transversal
Phase enc. dir.	A >> P
AutoAlign	---
Phase oversampling	20 %
FoV read	360 mm
FoV phase	75.0 %
Slice thickness	8.0 mm
TR	200.00 ms
TE 1	0.97 ms
TE 2	2.29 ms
TE 3	3.61 ms
TE 4	4.93 ms
TE 5	6.25 ms
TE 6	7.57 ms
TE 7	8.89 ms
TE 8	10.21 ms
TE 9	11.53 ms
TE 10	12.85 ms
TE 11	14.17 ms
TE 12	15.49 ms
Averages	1
Concatenations	1
Filter	Distortion Corr.(2D)
Coil elements	BO1-3;SP1-3

Contrast - Common

TR	200.00 ms
TE 1	0.97 ms
TE 2	2.29 ms
TE 3	3.61 ms
TE 4	4.93 ms
TE 5	6.25 ms
TE 6	7.57 ms
TE 7	8.89 ms
TE 8	10.21 ms
TE 9	11.53 ms
TE 10	12.85 ms
TE 11	14.17 ms
TE 12	15.49 ms
Flip angle	20 deg
Fat suppr.	Fat sat.
Wrap-up Magn.	None

Contrast - Dynamic

Averages	1
Averaging mode	Short term
Reconstruction	Magnitude
Measurements	1
Multiple series	Each measurement

Resolution - Common

FoV read	360 mm
FoV phase	75.0 %
Slice thickness	8.0 mm
Base resolution	128
Phase resolution	100 %
Phase partial Fourier	Off
Trajectory	Cartesian
Interpolation	Off

Resolution - iPAT

PAT mode	None

Resolution - Filter Image

Image Filter	Off
Distortion Corr.	On
Mode	2D
Unfiltered images	Off
Prescan Normalize	Off
Normalize	Off
B1 filter	Off

Resolution - Filter Rawdata

Raw filter	Off
Elliptical filter	Off
POCS	Off

Geometry - Common

Slice group	1
Slices	1
Dist. factor	20 %
Position	Isocenter
Orientation	Transversal
Phase enc. dir.	A >> P
FoV read	360 mm
FoV phase	75.0 %
Slice thickness	8.0 mm
TR	200.00 ms
Multi-slice mode	Sequential
Series	Ascending
Concatenations	1

Geometry - AutoAlign

Slice group	1
Position	Isocenter
Orientation	Transversal
Phase enc. dir.	A >> P
AutoAlign	---
Initial Position	Isocenter
Phase	0.0 mm
Read	0.0 mm
Shift	0.0 mm
Initial Rotation	0.00 deg
Initial Orientation	Transversal

Geometry - Saturation

Fat suppr.	Fat sat.
Wrap-up Magn.	None
Special sat.	None

Geometry - Navigator

System - Miscellaneous

Positioning mode	FIX
Table position	H
Table position	0 mm
MSMA	S - C - T
Sagittal	R >> L
Coronal	A >> P
Transversal	F >> H
Coil Combine Mode	Adaptive Combine
Save uncombined	Off
Matrix Optimization	Off
Coil Focus	Flat
AutoAlign	---
Coil Select Mode	Default

System - Adjustments

B0 Shim mode	Tune up
Adjust with body coil	Off
Confirm freq. adjustment	Off
Assume Dominant Fat	Off
Assume Silicone	Off
Adjustment Tolerance	Auto

System - Adjust Volume

! Position	Isocenter
! Orientation	Transversal
! Rotation	0.00 deg
! A >> P	263 mm
! R >> L	350 mm
! F >> H	350 mm
Reset	Off

System - Tx/Rx

Frequency 1H	63.678323 MHz
Correction factor	1
Gain	High
Img. Scale Cor.	1.000
Reset	Off
? Ref. amplitude 1H	0.000 V

Physio - Signal1

1st Signal/Mode	None
TR	200.00 ms
Concatenations	1
Segments	1

Physio - Cardiac

Tagging	None
Fat suppr.	Fat sat.
Dark blood	Off
FoV read	360 mm
FoV phase	75.0 %
Phase resolution	100 %
Cine	Off
Trajectory	Cartesian
Dummy heartbeats	0

Physio - PACE

Resp. control	Off
Concatenations	1

Inline - Common

Subtract	Off
Measurements	1
StdDev	Off
Save original images	On

Inline - Cardiac

Inline Evaluation	T2* map
Contrasts	12
TE 1	0.97 ms
TE 2	2.29 ms
TE 3	3.61 ms
TE 4	4.93 ms
TE 5	6.25 ms
TE 6	7.57 ms
TE 7	8.89 ms
TE 8	10.21 ms
TE 9	11.53 ms
TE 10	12.85 ms
TE 11	14.17 ms
TE 12	15.49 ms
TR	200.00 ms
Save original images	On

Inline - MIP

MIP-Sag	Off
MIP-Cor	Off
MIP-Tra	Off
MIP-Time	Off
Save original images	On

Inline - Composing

Inline Composing	Off
Distortion Corr.	On
Mode	2D
Unfiltered images	Off

Sequence - Part 1

Introduction	Off
Dimension	2D
Reordering	Linear
Asymmetric echo	Weak
Contrasts	12
Flow comp. 1	No
Readout mode	Monopolar
Optimization	Min. TE
Multi-slice mode	Sequential
Echo spacing	16.8 ms
Sequence type	Gre
Bandwidth 1	1953 Hz/Px
Bandwidth 2	1953 Hz/Px
Bandwidth 3	1953 Hz/Px
Bandwidth 4	1953 Hz/Px
Bandwidth 5	1953 Hz/Px
Bandwidth 6	1953 Hz/Px
Bandwidth 7	1953 Hz/Px
Bandwidth 8	1953 Hz/Px
Bandwidth 9	1953 Hz/Px
Bandwidth 10	1953 Hz/Px
Bandwidth 11	1953 Hz/Px
Bandwidth 12	1953 Hz/Px

Sequence - Part 2

Define	Segments
Segments	1
RF pulse type	Fast
Gradient mode	Fast
Excitation	Slice-sel.
Flip angle mode	Constant
RF spoiling	On
Phase Enc. Rewinder	On
Cine	Off

Sequence - Assistant

Mode	Off
Allowed delay	0 s

\\USER\Cardiac Research Protocols\IV IRON\IV IRON4\KLST2Map_TrueFISP
TA: 5.3 s PM: REF Voxel size: 2.4×2.4×8.0 mmPAT: 2 Rel. SNR: 1.00 : tfi

Properties

Prio recon	Off
Load images to viewer	On
Inline movie	Off
Auto store images	On
Load images to stamp segments	Off
Load images to graphic segments	On
Auto open inline display	Off
Auto close inline display	Off
Start measurement without further preparation	Off
Wait for user to start	On
Start measurements	Single measurement

Routine

Slice group	1
Slices	1
Dist. factor	20 %
Position	Isocenter
Orientation	Transversal
Phase enc. dir.	A >> P
AutoAlign	---
Phase oversampling	0 %
FoV read	460 mm
FoV phase	75.0 %
Slice thickness	8.0 mm
TR	179.50 ms
TE	1 ms
Averages	1
Concatenations	1
Filter	Distortion Corr.(2D)
Coil elements	BO1-3;SP1-3

Contrast - Common

TR	179.50 ms
TE	1 ms
Magn. preparation	T2 prep. adiab.
T2 prep. duration 1	0 ms
T2 prep. duration 2	25 ms
T2 prep. duration 3	55 ms
Flip angle	70 deg
Fat suppr.	None
Wrap-up Magn.	None

Contrast - Dynamic

Averages	1
Averaging mode	Short term
Reconstruction	Magnitude
Measurements	1
Multiple series	Off

Resolution - Common

FoV read	460 mm
FoV phase	75.0 %
Slice thickness	8.0 mm
Base resolution	192
Phase resolution	75 %
Phase partial Fourier	6/8
Trajectory	Cartesian
Interpolation	Off

Resolution - iPAT

PAT mode	GRAPPA
Accel. factor PE	2
Ref. lines PE	36
Reference scan mode	GRE/separate

Resolution - Filter Image

Image Filter	Off
Distortion Corr.	On
Mode	2D
Unfiltered images	Off
Prescan Normalize	Off
Normalize	Off
B1 filter	Off

Resolution - Filter Rawdata

Raw filter	Off
Elliptical filter	Off
POCS	Off

Geometry - Common

Slice group	1
Slices	1
Dist. factor	20 %
Position	Isocenter
Orientation	Transversal
Phase enc. dir.	A >> P
FoV read	460 mm
FoV phase	75.0 %
Slice thickness	8.0 mm
TR	179.50 ms
Multi-slice mode	Sequential
Series	Base To Apex
Concatenations	1

Geometry - AutoAlign

Slice group	1
Position	Isocenter
Orientation	Transversal
Phase enc. dir.	A >> P
AutoAlign	---
Initial Position	Isocenter
Phase	0.0 mm
Read	0.0 mm
Shift	0.0 mm
Initial Rotation	0.00 deg
Initial Orientation	Transversal

Geometry - Saturation

Fat suppr.	None
Wrap-up Magn.	None
Special sat.	None

Geometry - Navigator

System - Miscellaneous

Positioning mode	REF
Table position	H
Table position	0 mm
MSMA	S - C - T
Sagittal	R >> L

System - Miscellaneous

Coronal	A >> P
Transversal	F >> H
Coil Combine Mode	Adaptive Combine
Save uncombined	Off
Matrix Optimization	Off
Coil Focus	Flat
AutoAlign	---
Coil Select Mode	Default

System - Adjustments

B0 Shim mode	Tune up
Adjust with body coil	Off
Confirm freq. adjustment	Off
Assume Dominant Fat	Off
Assume Silicone	Off
Adjustment Tolerance	Auto

System - Adjust Volume

Position	Isocenter
Orientation	Transversal
Rotation	0.00 deg
A >> P	263 mm
R >> L	350 mm
F >> H	350 mm
Reset	Off

System - Tx/Rx

Frequency 1H	63.678323 MHz
Correction factor	1
Gain	High
Img. Scale Cor.	1.000
Reset	Off
? Ref. amplitude 1H	0.000 V

Physio - Signal1

1st Signal/Mode	ECG/Trigger
Average cycle	No Signal ms
Average cycle	No Signal ms
Captured cycle	-not set-
Acquisition window	591 ms
Trigger pulse	1
Trigger delay	411 ms
TR	179.50 ms
Concatenations	1
Segments	54
Phases	1
Adaptive Triggering	Off

Physio - Cardiac

Tagging	None
Magn. preparation	T2 prep. adiab.
T2 prep. duration 1	0 ms
T2 prep. duration 2	25 ms
T2 prep. duration 3	55 ms
Fat suppr.	None
Dark blood	Off
FoV read	460 mm
FoV phase	75.0 %
Phase resolution	75 %
Cine	Off
Trajectory	Cartesian
Dummy heartbeats	0
Motion Correction	Standard

Physio - PACE

Resp. control	Breath-hold
Concatenations	1

Sequence - Part 1

Introduction	Off
Dimension	2D
Reordering	Linear
Asymmetric echo	Weak
Contrasts	1
Optimization	Min. TE TR
Multi-slice mode	Sequential
Sequence type	Trufi
Bandwidth	1184 Hz/Px

Sequence - Part 2

Define	Shots
Shots per slice	1
Segments	54
Trufi delta freq.	0 Hz
RF pulse type	Fast
Gradient mode	Fast
Excitation	Slice-sel.
Flip angle mode	Constant
Cine	Off

Sequence - Assistant

Mode	Off
Allowed delay	0 s

\\USER\Cardiac Research Protocols\IV IRON\IV IRON4\KLSShMOLLI_C2P_m
TA: 5.3 s PM: FIX Voxel size: 1.2×1.2×8.0 mmPAT: 2 Rel. SNR: 1.00 : tfi

Properties

Prio recon	Off
Load images to viewer	On
Inline movie	Off
Auto store images	On
Load images to stamp segments	Off
Load images to graphic segments	On
Auto open inline display	Off
Auto close inline display	Off
Start measurement without further preparation	On
Wait for user to start	Off
Start measurements	Single measurement

Routine

Slice group	1
Slices	1
Dist. factor	25 %
Position	Isocenter
Orientation	Transversal
Phase enc. dir.	A >> P
AutoAlign	---
Phase oversampling	0 %
FoV read	460 mm
FoV phase	75.0 %
Slice thickness	8.0 mm
TR	375.62 ms
TE	1.07 ms
Averages	1
Concatenations	1
Filter	Raw filter, Distortion Corr.(2D)
Coil elements	BO1-3;SP1-3

Contrast - Common

TR	375.62 ms
TE	1.07 ms
Magn. preparation	Non-sel. IR
TI	260 ms
Flip angle	35 deg
Fat suppr.	None
Wrap-up Magn.	None

Contrast - Dynamic

Averages	1
Averaging mode	Short term
Reconstruction	Magn./Phase
Measurements	1
Multiple series	Off

Resolution - Common

FoV read	460 mm
FoV phase	75.0 %
Slice thickness	8.0 mm
Base resolution	192
Phase resolution	100 %
Phase partial Fourier	6/8
Trajectory	Cartesian
Interpolation	On

Resolution - iPAT

PAT mode	GRAPPA
Accel. factor PE	2
Ref. lines PE	24
Reference scan mode	Integrated

Resolution - Filter Image

Image Filter	Off
Distortion Corr.	On
Mode	2D
Unfiltered images	Off
Prescan Normalize	Off
Normalize	Off
B1 filter	Off

Resolution - Filter Rawdata

Raw filter	On
Elliptical filter	Off
POCS	Off

Geometry - Common

Slice group	1
Slices	1
Dist. factor	25 %
Position	Isocenter
Orientation	Transversal
Phase enc. dir.	A >> P
FoV read	460 mm
FoV phase	75.0 %
Slice thickness	8.0 mm
TR	375.62 ms
Multi-slice mode	Sequential
Series	Interleaved
Concatenations	1

Geometry - AutoAlign

Slice group	1
Position	Isocenter
Orientation	Transversal
Phase enc. dir.	A >> P
AutoAlign	---
Initial Position	Isocenter
Phase	0.0 mm
Read	0.0 mm
Shift	0.0 mm
Initial Rotation	0.00 deg
Initial Orientation	Transversal

Geometry - Saturation

Fat suppr.	None
Wrap-up Magn.	None
Special sat.	None

Geometry - Navigator

System - Miscellaneous

Positioning mode	FIX
Table position	H
Table position	0 mm
MSMA	S - C - T
Sagittal	R >> L

System - Miscellaneous

Coronal	A >> P
Transversal	F >> H
Coil Combine Mode	Adaptive Combine
Save uncombined	Off
Matrix Optimization	Off
Coil Focus	Flat
AutoAlign	---
Coil Select Mode	Default

System - Adjustments

B0 Shim mode	Tune up
Adjust with body coil	On
Confirm freq. adjustment	Off
Assume Dominant Fat	Off
Assume Silicone	Off
Adjustment Tolerance	Auto

System - Adjust Volume

! Position	Isocenter
! Orientation	Transversal
! Rotation	0.00 deg
! A >> P	263 mm
! R >> L	350 mm
! F >> H	350 mm
Reset	Off

System - Tx/Rx

Frequency 1H	63.678323 MHz
Correction factor	1
Gain	High
Img. Scale Cor.	1.000
Reset	Off
? Ref. amplitude 1H	0.000 V

Physio - Signal1

1st Signal/Mode	ECG/Trigger
Average cycle	No Signal ms
Average cycle	No Signal ms
Captured cycle	-not set-
Acquisition window	591 ms
Trigger pulse	1
Trigger delay	215 ms
TR	375.62 ms
Concatenations	1
Segments	84
Phases	1
Adaptive Triggering	Off

Physio - Cardiac

Tagging	None
Magn. preparation	Non-sel. IR
TI	260 ms
Fat suppr.	None
Dark blood	Off
FoV read	460 mm
FoV phase	75.0 %
Phase resolution	100 %
Cine	Off
Trajectory	Cartesian
Dummy heartbeats	0
Motion Correction	Standard

Physio - PACE

Resp. control	Off
Concatenations	1

Sequence - Part 1

Introduction	Off
Dimension	2D
Reordering	Linear
Asymmetric echo	Weak
Contrasts	1
Optimization	Min. TE TR
Multi-slice mode	Sequential
Sequence type	Trufi
Bandwidth	898 Hz/Px

Sequence - Part 2

Define	Shots
Shots per slice	1
Segments	84
Trufi delta freq.	0 Hz
RF pulse type	Fast
Gradient mode	Fast
Excitation	Slice-sel.
Flip angle mode	Constant
Cine	Off

Sequence - Assistant

Mode	Off
Allowed delay	0 s

\\USER\Cardiac Research Protocols\IV IRON\IV IRON4\KLST2StarMap_8echo_heart
TA: 9.4 s PM: FIX Voxel size: 1.8×1.8×8.0 mmPAT: 2 Rel. SNR: 1.00 : fl_r

Properties

Prio recon	Off
Load images to viewer	On
Inline movie	Off
Auto store images	On
Load images to stamp segments	Off
Load images to graphic segments	On
Auto open inline display	Off
Auto close inline display	Off
Start measurement without further preparation	On
Wait for user to start	Off
Start measurements	Single measurement

Routine

Slice group	1
Slices	1
Dist. factor	20 %
Position	Isocenter
Orientation	Transversal
Phase enc. dir.	A >> P
AutoAlign	---
Phase oversampling	0 %
FoV read	460 mm
FoV phase	75.0 %
Slice thickness	8.0 mm
TR	590.00 ms
TE 1	1.91 ms
TE 2	3.85 ms
TE 3	5.79 ms
TE 4	7.73 ms
TE 5	9.67 ms
TE 6	11.61 ms
TE 7	13.55 ms
TE 8	15.49 ms
Averages	1
Concatenations	1
Filter	Distortion Corr.(2D)
Coil elements	BO1-3;SP1-3

Contrast - Common

TR	590.00 ms
TE 1	1.91 ms
TE 2	3.85 ms
TE 3	5.79 ms
TE 4	7.73 ms
TE 5	9.67 ms
TE 6	11.61 ms
TE 7	13.55 ms
TE 8	15.49 ms
Flip angle	20 deg
Fat suppr.	Fat sat.
Wrap-up Magn.	None

Contrast - Dynamic

Averages	1
Averaging mode	Short term
Reconstruction	Magnitude
Measurements	1
Multiple series	Off

Resolution - Common

FoV read	460 mm
FoV phase	75.0 %
Slice thickness	8.0 mm
Base resolution	256
Phase resolution	60 %
Phase partial Fourier	Off
Trajectory	Cartesian
Interpolation	Off

Resolution - iPAT

PAT mode	GRAPPA
Accel. factor PE	2
Ref. lines PE	24
Reference scan mode	Integrated

Resolution - Filter Image

Image Filter	Off
Distortion Corr.	On
Mode	2D
Unfiltered images	Off
Prescan Normalize	Off
Normalize	Off
B1 filter	Off

Resolution - Filter Rawdata

Raw filter	Off
Elliptical filter	Off
POCS	Off

Geometry - Common

Slice group	1
Slices	1
Dist. factor	20 %
Position	Isocenter
Orientation	Transversal
Phase enc. dir.	A >> P
FoV read	460 mm
FoV phase	75.0 %
Slice thickness	8.0 mm
TR	590.00 ms
Multi-slice mode	Sequential
Series	Base To Apex
Concatenations	1

Geometry - AutoAlign

Slice group	1
Position	Isocenter
Orientation	Transversal
Phase enc. dir.	A >> P
AutoAlign	---
Initial Position	Isocenter
Phase	0.0 mm
Read	0.0 mm
Shift	0.0 mm
Initial Rotation	0.00 deg
Initial Orientation	Transversal

Geometry - Saturation

Fat suppr.	Fat sat.
Wrap-up Magn.	None

Geometry - Saturation

Special sat.	None

Geometry - Navigator

System - Miscellaneous

Positioning mode	FIX
Table position	H
Table position	0 mm
MSMA	S - C - T
Sagittal	R >> L
Coronal	A >> P
Transversal	F >> H
Coil Combine Mode	Adaptive Combine
Save uncombined	Off
Matrix Optimization	Off
Coil Focus	Flat
AutoAlign	---
Coil Select Mode	Default

System - Adjustments

B0 Shim mode	Tune up
Adjust with body coil	Off
Confirm freq. adjustment	Off
Assume Dominant Fat	Off
Assume Silicone	Off
Adjustment Tolerance	Auto

System - Adjust Volume

! Position	Isocenter
! Orientation	Transversal
! Rotation	0.00 deg
! A >> P	263 mm
! R >> L	350 mm
! F >> H	350 mm
Reset	Off

System - Tx/Rx

Frequency 1H	63.678323 MHz
Correction factor	1
Gain	High
Img. Scale Cor.	1.000
Reset	Off
? Ref. amplitude 1H	0.000 V

Physio - Signal1

1st Signal/Mode	ECG/Trigger
Average cycle	No Signal ms
Average cycle	No Signal ms
Captured cycle	-not set-
Acquisition window	590 ms
Trigger pulse	2
Trigger delay	0 ms
TR	590.00 ms
Concatenations	1
Segments	9
Phases	1
Adaptive Triggering	Off

Physio - Cardiac

Tagging	None
Fat suppr.	Fat sat.
Dark blood	On
Dark blood thickness	200 %

Physio - Cardiac

FoV read	460 mm
FoV phase	75.0 %
Phase resolution	60 %
Cine	Off
Trajectory	Cartesian
Dummy heartbeats	0

Physio - PACE

Resp. control	Breath-hold
Concatenations	1

Inline - Common

Subtract	Off
Measurements	1
StdDev	Off
Save original images	On

Inline - Cardiac

Inline Evaluation	T2* map
Contrasts	8
TE 1	1.91 ms
TE 2	3.85 ms
TE 3	5.79 ms
TE 4	7.73 ms
TE 5	9.67 ms
TE 6	11.61 ms
TE 7	13.55 ms
TE 8	15.49 ms
TR	590.00 ms
Save original images	On

Inline - MIP

MIP-Sag	Off
MIP-Cor	Off
MIP-Tra	Off
MIP-Time	Off
Save original images	On

Inline - Composing

Inline Composing	Off
Distortion Corr.	On
Mode	2D
Unfiltered images	Off

Sequence - Part 1

Introduction	Off
Dimension	2D
Reordering	Linear
Asymmetric echo	Weak
Contrasts	8
Flow comp. 1	Yes
Readout mode	Monopolar
Optimization	Min. TE
Multi-slice mode	Sequential
Echo spacing	17.4 ms
Sequence type	Gre
Bandwidth 1	814 Hz/Px
Bandwidth 2	814 Hz/Px
Bandwidth 3	814 Hz/Px
Bandwidth 4	814 Hz/Px
Bandwidth 5	814 Hz/Px
Bandwidth 6	814 Hz/Px
Bandwidth 7	814 Hz/Px
Bandwidth 8	814 Hz/Px

Sequence - Part 2

Define	Segments
Segments	9
RF pulse type	Fast
Gradient mode	Fast
Excitation	Slice-sel.
Flip angle mode	Constant
RF spoiling	On
Phase Enc. Rewinder	On
Cine	Off

Sequence - Assistant

Mode	Off
Allowed delay	0 s

\\USER\Cardiac Research Protocols\IV IRON\IV IRON4\KLSFB_MOCO_gt_T2star_DB_8e_128 FS_HiFE

TA: 9.4 s PM: FIX Voxel size: 3.6×3.6×8.0 mmPAT: 4 Rel. SNR: 1.00 : tfl

Properties

Prio recon	Off
Load images to viewer	On
Inline movie	Off
Auto store images	On
Load images to stamp segments	Off
Load images to graphic segments	On
Auto open inline display	Off
Auto close inline display	Off
Start measurement without further preparation	On
Wait for user to start	Off
Start measurements	Single measurement

Routine

Slice group	1
Slices	1
Dist. factor	20 %
Position	Isocenter
Orientation	Transversal
Phase enc. dir.	A >> P
AutoAlign	---
Phase oversampling	0 %
FoV read	460 mm
FoV phase	75.0 %
Slice thickness	8.0 mm
TR	590.00 ms
TE 1	1.07 ms
TE 2	2.42 ms
TE 3	3.77 ms
TE 4	5.12 ms
TE 5	6.47 ms
TE 6	7.82 ms
TE 7	9.17 ms
TE 8	10.52 ms
Averages	1
Concatenations	1
Filter	Distortion Corr.(2D)
Coil elements	BO1-3;SP1-3

Contrast - Common

TR	590.00 ms
TE 1	1.07 ms
TE 2	2.42 ms
TE 3	3.77 ms
TE 4	5.12 ms
TE 5	6.47 ms
TE 6	7.82 ms
TE 7	9.17 ms
TE 8	10.52 ms
Magn. preparation	None
Flip angle	18 deg
Fat suppr.	Fat sat.
Wrap-up Magn.	None

Contrast - Dynamic

Averages	1
Averaging mode	Long term
Reconstruction	Magnitude
Measurements	16

Contrast - Dynamic

Pause after meas. 1	0.0 s
Pause after meas. 2	0.0 s
Pause after meas. 3	0.0 s
Pause after meas. 4	0.0 s
Pause after meas. 5	0.0 s
Pause after meas. 6	0.0 s
Pause after meas. 7	0.0 s
Pause after meas. 8	0.0 s
Pause after meas. 9	0.0 s
Pause after meas. 10	0.0 s
Pause after meas. 11	0.0 s
Pause after meas. 12	0.0 s
Pause after meas. 13	0.0 s
Pause after meas. 14	0.0 s
Pause after meas. 15	0.0 s
Multiple series	Off

Resolution - Common

FoV read	460 mm
FoV phase	75.0 %
Slice thickness	8.0 mm
Base resolution	128
Phase resolution	100 %
Phase partial Fourier	Off
Trajectory	Cartesian
Interpolation	Off

Resolution - iPAT

PAT mode	GRAPPA
Accel. factor PE	4
Reference scan mode	T-PAT

Resolution - Filter Image

Image Filter	Off
Distortion Corr.	On
Mode	2D
Unfiltered images	Off
Prescan Normalize	Off
Normalize	Off
B1 filter	Off

Resolution - Filter Rawdata

Raw filter	Off
Elliptical filter	Off
POCS	Off

Geometry - Common

Slice group	1
Slices	1
Dist. factor	20 %
Position	Isocenter
Orientation	Transversal
Phase enc. dir.	A >> P
FoV read	460 mm
FoV phase	75.0 %
Slice thickness	8.0 mm
TR	590.00 ms
Multi-slice mode	Single shot
Series	Interleaved

Geometry - Common

Concatenations	1

Geometry - AutoAlign

Slice group	1
Position	Isocenter
Orientation	Transversal
Phase enc. dir.	A >> P
AutoAlign	---
Initial Position	Isocenter
Phase	0.0 mm
Read	0.0 mm
Shift	0.0 mm
Initial Rotation	0.00 deg
Initial Orientation	Transversal

Geometry - Saturation

Fat suppr.	Fat sat.
Wrap-up Magn.	None
Special sat.	None

Geometry - Navigator

System - Miscellaneous

Positioning mode	FIX
Table position	H
Table position	0 mm
MSMA	S - C - T
Sagittal	R >> L
Coronal	A >> P
Transversal	F >> H
Coil Combine Mode	Adaptive Combine
Save uncombined	Off
Matrix Optimization	Off
Coil Focus	Flat
AutoAlign	---
Coil Select Mode	Default

System - Adjustments

B0 Shim mode	Tune up
Adjust with body coil	On
Confirm freq. adjustment	Off
Assume Dominant Fat	Off
Assume Silicone	Off
Adjustment Tolerance	Auto

System - Adjust Volume

! Position	Isocenter
! Orientation	Transversal
! Rotation	0.00 deg
! A >> P	263 mm
! R >> L	350 mm
! F >> H	350 mm
Reset	Off

System - Tx/Rx

Frequency 1H	63.678323 MHz
Correction factor	1
Gain	High
Img. Scale Cor.	1.000
Reset	Off
? Ref. amplitude 1H	0.000 V

Physio - Signal1

1st Signal/Mode	ECG/Trigger
Average cycle	No Signal ms
Average cycle	No Signal ms
Captured cycle	-not set-
Acquisition window	590 ms
Trigger pulse	1
Trigger delay	0 ms
TR	590.00 ms
Concatenations	1
Segments	24
Phases	1
Adaptive Triggering	Off

Physio - Cardiac

Tagging	None
Magn. preparation	None
Fat suppr.	Fat sat.
Dark blood	On
Dark blood thickness	300 %
FoV read	460 mm
FoV phase	75.0 %
Phase resolution	100 %
Cine	Off
Trajectory	Cartesian
Dummy heartbeats	0

Physio - PACE

Resp. control	Off
Concatenations	1

Inline - Common

Subtract	Off
Measurements	16
StdDev	Off
Save original images	On

Inline - Cardiac

Inline Evaluation	Off
Magn. preparation	None
Contrasts	8
TE 1	1.07 ms
TE 2	2.42 ms
TE 3	3.77 ms
TE 4	5.12 ms
TE 5	6.47 ms
TE 6	7.82 ms
TE 7	9.17 ms
TE 8	10.52 ms
TR	590.00 ms
Save original images	On

Inline - MIP

MIP-Sag	Off
MIP-Cor	Off
MIP-Tra	Off
MIP-Time	Off
Save original images	On

Inline - Composing

Inline Composing	Off
Distortion Corr.	On
Mode	2D
Unfiltered images	Off

Sequence - Part 1

Introduction	Off
Dimension	2D
Reordering	Linear
Asymmetric echo	Off
Contrasts	8
Flow comp. 1	No
Readout mode	Monopolar
Optimization	Min. TE
Multi-slice mode	Single shot
Echo spacing	11.4 ms
Sequence type	Gre
Bandwidth 1	1502 Hz/Px
Bandwidth 2	1502 Hz/Px
Bandwidth 3	1502 Hz/Px
Bandwidth 4	1502 Hz/Px
Bandwidth 5	1502 Hz/Px
Bandwidth 6	1502 Hz/Px
Bandwidth 7	1502 Hz/Px
Bandwidth 8	1502 Hz/Px

Sequence - Part 2

Define	Shots
Shots per slice	1
Segments	24
RF pulse type	Fast
Gradient mode	Fast
Excitation	Slice-sel.
Flip angle mode	Constant
RF spoiling	On
Phase Enc. Rewinder	On
Cine	Off

Sequence - Special

FatWater Separation	On
Multi-echo Images	Off
In-Opp Phase Images	Off
Frequency Map	Off
T2* Map	Off
Motion Correction	Off
MoCo Averaging Mode	Complex MoCo
MoCo Images Only?	Off
No. of Interleaves	0

Sequence - Assistant

Mode	Off
Allowed delay	0 s

\\USER\Cardiac Research Protocols\IV IRON\IV IRON4\KLSFB_MOCO_gt_T2star_DB_8e_160 FS_LowNFE
TA: 9.4 s PM: FIX Voxel size: 2.9×2.9×8.0 mmPAT: 4 Rel. SNR: 1.00 : tfl

Properties

Prio recon	Off
Load images to viewer	On
Inline movie	Off
Auto store images	On
Load images to stamp segments	Off
Load images to graphic segments	On
Auto open inline display	Off
Auto close inline display	Off
Start measurement without further preparation	On
Wait for user to start	Off
Start measurements	Single measurement

Routine

Slice group	1
Slices	1
Dist. factor	20 %
Position	Isocenter
Orientation	Transversal
Phase enc. dir.	A >> P
AutoAlign	---
Phase oversampling	0 %
FoV read	460 mm
FoV phase	75.0 %
Slice thickness	8.0 mm
TR	590.00 ms
TE 1	1.2 ms
TE 2	2.87 ms
TE 3	4.54 ms
TE 4	6.21 ms
TE 5	7.88 ms
TE 6	9.55 ms
TE 7	11.22 ms
TE 8	12.89 ms
Averages	1
Concatenations	1
Filter	Distortion Corr.(2D)
Coil elements	BO1-3;SP1-3

Contrast - Common

TR	590.00 ms
TE 1	1.2 ms
TE 2	2.87 ms
TE 3	4.54 ms
TE 4	6.21 ms
TE 5	7.88 ms
TE 6	9.55 ms
TE 7	11.22 ms
TE 8	12.89 ms
Magn. preparation	None
Flip angle	18 deg
Fat suppr.	Fat sat.
Wrap-up Magn.	None

Contrast - Dynamic

Averages	1
Averaging mode	Long term
Reconstruction	Magnitude
Measurements	16

Contrast - Dynamic

Pause after meas. 1	0.0 s
Pause after meas. 2	0.0 s
Pause after meas. 3	0.0 s
Pause after meas. 4	0.0 s
Pause after meas. 5	0.0 s
Pause after meas. 6	0.0 s
Pause after meas. 7	0.0 s
Pause after meas. 8	0.0 s
Pause after meas. 9	0.0 s
Pause after meas. 10	0.0 s
Pause after meas. 11	0.0 s
Pause after meas. 12	0.0 s
Pause after meas. 13	0.0 s
Pause after meas. 14	0.0 s
Pause after meas. 15	0.0 s
Multiple series	Off

Resolution - Common

FoV read	460 mm
FoV phase	75.0 %
Slice thickness	8.0 mm
Base resolution	160
Phase resolution	87 %
Phase partial Fourier	Off
Trajectory	Cartesian
Interpolation	Off

Resolution - iPAT

PAT mode	GRAPPA
Accel. factor PE	4
Reference scan mode	T-PAT

Resolution - Filter Image

Image Filter	Off
Distortion Corr.	On
Mode	2D
Unfiltered images	Off
Prescan Normalize	Off
Normalize	Off
B1 filter	Off

Resolution - Filter Rawdata

Raw filter	Off
Elliptical filter	Off
POCS	Off

Geometry - Common

Slice group	1
Slices	1
Dist. factor	20 %
Position	Isocenter
Orientation	Transversal
Phase enc. dir.	A >> P
FoV read	460 mm
FoV phase	75.0 %
Slice thickness	8.0 mm
TR	590.00 ms
Multi-slice mode	Single shot
Series	Interleaved

Geometry - Common

Concatenations	1

Geometry - AutoAlign

Slice group	1
Position	Isocenter
Orientation	Transversal
Phase enc. dir.	A >> P
AutoAlign	---
Initial Position	Isocenter
Phase	0.0 mm
Read	0.0 mm
Shift	0.0 mm
Initial Rotation	0.00 deg
Initial Orientation	Transversal

Geometry - Saturation

Fat suppr.	Fat sat.
Wrap-up Magn.	None
Special sat.	None

Geometry - Navigator

System - Miscellaneous

Positioning mode	FIX
Table position	H
Table position	0 mm
MSMA	S - C - T
Sagittal	R >> L
Coronal	A >> P
Transversal	F >> H
Coil Combine Mode	Adaptive Combine
Save uncombined	Off
Matrix Optimization	Off
Coil Focus	Flat
AutoAlign	---
Coil Select Mode	Default

System - Adjustments

B0 Shim mode	Tune up
Adjust with body coil	On
Confirm freq. adjustment	Off
Assume Dominant Fat	Off
Assume Silicone	Off
Adjustment Tolerance	Auto

System - Adjust Volume

! Position	Isocenter
! Orientation	Transversal
! Rotation	0.00 deg
! A >> P	263 mm
! R >> L	350 mm
! F >> H	350 mm
Reset	Off

System - Tx/Rx

Frequency 1H	63.678323 MHz
Correction factor	1
Gain	High
Img. Scale Cor.	1.000
Reset	Off
? Ref. amplitude 1H	0.000 V

Physio - Signal1

1st Signal/Mode	ECG/Trigger
Average cycle	No Signal ms
Average cycle	No Signal ms
Captured cycle	-not set-
Acquisition window	590 ms
Trigger pulse	1
Trigger delay	0 ms
TR	590.00 ms
Concatenations	1
Segments	26
Phases	1
Adaptive Triggering	Off

Physio - Cardiac

Tagging	None
Magn. preparation	None
Fat suppr.	Fat sat.
Dark blood	On
Dark blood thickness	300 %
FoV read	460 mm
FoV phase	75.0 %
Phase resolution	87 %
Cine	Off
Trajectory	Cartesian
Dummy heartbeats	0

Physio - PACE

Resp. control	Off
Concatenations	1

Inline - Common

Subtract	Off
Measurements	16
StdDev	Off
Save original images	On

Inline - Cardiac

Inline Evaluation	Off
Magn. preparation	None
Contrasts	8
TE 1	1.2 ms
TE 2	2.87 ms
TE 3	4.54 ms
TE 4	6.21 ms
TE 5	7.88 ms
TE 6	9.55 ms
TE 7	11.22 ms
TE 8	12.89 ms
TR	590.00 ms
Save original images	On

Inline - MIP

MIP-Sag	Off
MIP-Cor	Off
MIP-Tra	Off
MIP-Time	Off
Save original images	On

Inline - Composing

Inline Composing	Off
Distortion Corr.	On
Mode	2D
Unfiltered images	Off

Sequence - Part 1

Introduction	Off
Dimension	2D
Reordering	Linear
Asymmetric echo	Off
Contrasts	8
Flow comp. 1	No
Readout mode	Monopolar
Optimization	Min. TE
Multi-slice mode	Single shot
Echo spacing	13.9 ms
Sequence type	Gre
Bandwidth 1	1078 Hz/Px
Bandwidth 2	1078 Hz/Px
Bandwidth 3	1078 Hz/Px
Bandwidth 4	1078 Hz/Px
Bandwidth 5	1078 Hz/Px
Bandwidth 6	1078 Hz/Px
Bandwidth 7	1078 Hz/Px
Bandwidth 8	1078 Hz/Px

Sequence - Part 2

Define	Shots
Shots per slice	1
Segments	26
RF pulse type	Fast
Gradient mode	Fast
Excitation	Slice-sel.
Flip angle mode	Constant
RF spoiling	On
Phase Enc. Rewinder	On
Cine	Off

Sequence - Special

FatWater Separation	On
Multi-echo Images	Off
In-Opp Phase Images	Off
Frequency Map	Off
T2* Map	Off
Motion Correction	Off
MoCo Averaging Mode	Complex MoCo
MoCo Images Only?	Off
No. of Interleaves	0

Sequence - Assistant

Mode	Off
Allowed delay	0 s

\\USER\Cardiac Research Protocols\IV IRON\IV IRON4\KLSnonBH_T2StarMap_12echo_liver
TA: 0:23 PM: FIX Voxel size: 3.6×3.6×8.0 mmPAT: Off Rel. SNR: 1.00 : fl

Properties

Prio recon	Off
Load images to viewer	On
Inline movie	Off
Auto store images	On
Load images to stamp segments	Off
Load images to graphic segments	On
Auto open inline display	Off
Auto close inline display	Off
Start measurement without further preparation	On
Wait for user to start	Off
Start measurements	Single measurement

Routine

Slice group	1
Slices	1
Dist. factor	20 %
Position	Isocenter
Orientation	Transversal
Phase enc. dir.	A >> P
AutoAlign	---
Phase oversampling	20 %
FoV read	460 mm
FoV phase	75.0 %
Slice thickness	8.0 mm
TR	200.00 ms
TE 1	0.92 ms
TE 2	2.07 ms
TE 3	3.22 ms
TE 4	4.37 ms
TE 5	5.52 ms
TE 6	6.67 ms
TE 7	7.82 ms
TE 8	8.97 ms
TE 9	10.12 ms
TE 10	11.27 ms
TE 11	12.42 ms
TE 12	13.57 ms
Averages	1
Concatenations	1
Filter	Distortion Corr.(2D)
Coil elements	BO1-3;SP1-3

Contrast - Common

TR	200.00 ms
TE 1	0.92 ms
TE 2	2.07 ms
TE 3	3.22 ms
TE 4	4.37 ms
TE 5	5.52 ms
TE 6	6.67 ms
TE 7	7.82 ms
TE 8	8.97 ms
TE 9	10.12 ms
TE 10	11.27 ms
TE 11	12.42 ms
TE 12	13.57 ms
Flip angle	20 deg
Fat suppr.	Fat sat.
Wrap-up Magn.	None

Contrast - Dynamic

Averages	1
Averaging mode	Short term
Reconstruction	Magnitude
Measurements	1
Multiple series	Each measurement

Resolution - Common

FoV read	460 mm
FoV phase	75.0 %
Slice thickness	8.0 mm
Base resolution	128
Phase resolution	100 %
Phase partial Fourier	Off
Trajectory	Cartesian
Interpolation	Off

Resolution - iPAT

PAT mode	None

Resolution - Filter Image

Image Filter	Off
Distortion Corr.	On
Mode	2D
Unfiltered images	Off
Prescan Normalize	Off
Normalize	Off
B1 filter	Off

Resolution - Filter Rawdata

Raw filter	Off
Elliptical filter	Off
POCS	Off

Geometry - Common

Slice group	1
Slices	1
Dist. factor	20 %
Position	Isocenter
Orientation	Transversal
Phase enc. dir.	A >> P
FoV read	460 mm
FoV phase	75.0 %
Slice thickness	8.0 mm
TR	200.00 ms
Multi-slice mode	Sequential
Series	Ascending
Concatenations	1

Geometry - AutoAlign

Slice group	1
Position	Isocenter
Orientation	Transversal
Phase enc. dir.	A >> P
AutoAlign	---
Initial Position	Isocenter
Phase	0.0 mm
Read	0.0 mm
Shift	0.0 mm
Initial Rotation	0.00 deg
Initial Orientation	Transversal

Geometry - Saturation

Fat suppr.	Fat sat.
Wrap-up Magn.	None
Special sat.	None

Geometry - Navigator

System - Miscellaneous

Positioning mode	FIX
Table position	H
Table position	0 mm
MSMA	S - C - T
Sagittal	R >> L
Coronal	A >> P
Transversal	F >> H
Coil Combine Mode	Adaptive Combine
Save uncombined	Off
Matrix Optimization	Off
Coil Focus	Flat
AutoAlign	---
Coil Select Mode	Default

System - Adjustments

B0 Shim mode	Tune up
Adjust with body coil	Off
Confirm freq. adjustment	Off
Assume Dominant Fat	Off
Assume Silicone	Off
Adjustment Tolerance	Auto

System - Adjust Volume

! Position	Isocenter
! Orientation	Transversal
! Rotation	0.00 deg
! A >> P	263 mm
! R >> L	350 mm
! F >> H	350 mm
Reset	Off

System - Tx/Rx

Frequency 1H	63.678323 MHz
Correction factor	1
Gain	High
Img. Scale Cor.	1.000
Reset	Off
? Ref. amplitude 1H	0.000 V

Physio - Signal1

1st Signal/Mode	None
TR	200.00 ms
Concatenations	1
Segments	1

Physio - Cardiac

Tagging	None
Fat suppr.	Fat sat.
Dark blood	Off
FoV read	460 mm
FoV phase	75.0 %
Phase resolution	100 %
Cine	Off
Trajectory	Cartesian
Dummy heartbeats	0

Physio - PACE

Resp. control	Off
Concatenations	1

Inline - Common

Subtract	Off
Measurements	1
StdDev	Off
Save original images	On

Inline - Cardiac

Inline Evaluation	T2* map
Contrasts	12
TE 1	0.92 ms
TE 2	2.07 ms
TE 3	3.22 ms
TE 4	4.37 ms
TE 5	5.52 ms
TE 6	6.67 ms
TE 7	7.82 ms
TE 8	8.97 ms
TE 9	10.12 ms
TE 10	11.27 ms
TE 11	12.42 ms
TE 12	13.57 ms
TR	200.00 ms
Save original images	On

Inline - MIP

MIP-Sag	Off
MIP-Cor	Off
MIP-Tra	Off
MIP-Time	Off
Save original images	On

Inline - Composing

Inline Composing	Off
Distortion Corr.	On
Mode	2D
Unfiltered images	Off

Sequence - Part 1

Introduction	Off
Dimension	2D
Reordering	Linear
Asymmetric echo	Weak
Contrasts	12
Flow comp. 1	No
Readout mode	Monopolar
Optimization	Min. TE
Multi-slice mode	Sequential
Echo spacing	14.8 ms
Sequence type	Gre
Bandwidth 1	1953 Hz/Px
Bandwidth 2	1953 Hz/Px
Bandwidth 3	1953 Hz/Px
Bandwidth 4	1953 Hz/Px
Bandwidth 5	1953 Hz/Px
Bandwidth 6	1953 Hz/Px
Bandwidth 7	1953 Hz/Px
Bandwidth 8	1953 Hz/Px
Bandwidth 9	1953 Hz/Px
Bandwidth 10	1953 Hz/Px
Bandwidth 11	1953 Hz/Px
Bandwidth 12	1953 Hz/Px

Sequence - Part 2

Define	Segments
Segments	1
RF pulse type	Fast
Gradient mode	Fast
Excitation	Slice-sel.
Flip angle mode	Constant
RF spoiling	On
Phase Enc. Rewinder	On
Cine	Off

Sequence - Assistant

Mode	Off
Allowed delay	0 s
