# Supplementary material for: Intravenous iron therapy results in rapid and sustained rise in myocardial iron content through a novel pathway
Source: Eur Heart J. 2024 Jun 25;45(42):4497–508. doi: 10.1093/eurheartj/ehae359 (PMC11544312; doi:10.1093/eurheartj/ehae359)
Supplement: ehae359_Supplementary_Data [file ehae359_supplementary_data.zip › Supplemental File 5.pdf]

Supplemental Methods

Mice

All animal procedures were compliant with the UK Home Office Animals (Scientific Procedures) Act 1986 (licence# P84F13B1B) and approved by the University of Oxford Medical Sciences Division Ethical Review Committee. Bioluminescence studies used B6;FVB-Ptprca Tg(CAG-luc,-GFP)L2G85Chco Thy1a/J mice (JAX strain #025854), which harbour the firefly luciferase transgene under control of CAG promoter; composed of human cytomegalovirus immediate early promoter enhancer with chicken beta-actin/rabbit beta-globin hybrid promoter. These mice have previously been characterised as having bioluminescence in the heart, spleen, muscle, pancreas, skin, thymus and bone marrow, but not in mature erythrocytes[36]. Mice were housed under standard conditions in individually-ventilated cages with enrichment at a density of 2-4 animals per cage. A priori humane endpoints were weight loss ≥15%, or signs of ill health (hunched posture and reduced activity) that did not resolve within 24 hours. Each animal represents an experimental unit. All animals were given an identifier code, to allow blinding during the conduct and analysis of experiments. Power calculations were not used due to lack of previous data on myocardial LIP.

Manipulation of iron status in mice

Mice heterozygous for luciferase transgene were randomly assigned to a standard iron-replete diet containing 200 ppm iron (n=22), or an iron-deficient diet containing 5 ppm iron (n=25) (Teklad TD.99397), which were provided for 6 weeks.
Within each dietary group, mice were then randomly assigned to receive a 100µl injection via the tail vein of either saline (n=11 for iron-replete diet, n=12 for iron-deficient diet) or iron as ferric carboxymaltose (Ferinject® [FCM], Vifor Pharma, Glattbrugg, Switzerland) at 15mg/kg (n=11 for iron-replete group, n=13 for iron-deficient group). Mice were used for bioluminescence imaging either 1 hour or 42 days post infusion. To minimise any potential confounding effects of age and sex, all animals entered the diary protocol at 6 weeks of age, and block randomisation was used to ensure balanced numbers of males and females in each group. Randomisation into diets and treatments was carried out using the Rand() function in excel.

Labile iron imaging in murine hearts

Bioluminescence studies used the iron-caged luciferin described previously[32].
Mice were anaesthetised using 2% isoflurane in O_2, and injected via the tail vein with 25nmoles of iron-caged luciferin (ICL-1) dissolved in 100ul saline. One minute later, the chest cavity was opened, and the heart immediately surgically excised, washed in ice-cold PBS to remove any blood clots, and placed on a petri dish within the IVIS Lumina system. Bioluminescence imaging was performed in an IVIS LUMINA II system, with small binning and F stop 1 settings. Bioluminescence signal was collected for at least 2 minutes, with auto settings. Luminescence data were extracted using Living Image Software4.7.3. First, a non-luminescence photograph was used to generate ROIs, by manually drawing around the outline of each heart (excluding atria). ROIs corresponding to individual hearts were automatically propagated onto the luminescence sequence, and average radiance extracted from each ROI as p/s/cm²/sr (photons, per second, per square cm, per steradian). The mean of the average radiance values acquired over the first two minutes were calculated for each heart.
Following removal of the heart, blood was immediately collected from the chest cavity for Hb measurement and preparation of serum. Spleens and livers were also removed, washed in ice-cold PBS before snap-freezing for quantitation of total iron content.
No animals were lost to humane endpoints. However, some animals did not undergo myocardial LIP imaging due to failure to infuse ICL-1 intravenously prior to imaging. Their spleens and livers were still harvested for direct iron quantitation.

Iron quantitation in murine tissues

For total elemental iron analysis, snap-frozen hearts, livers and spleens were crushed on liquid nitrogen, and a minimum of 5mg digested in nitric acid using a CEM microwave system. Elemental component analysis was carried out using induced couple plasma mass spectrometry ICP-MS as per previous studies [37]. Values are normalised to tissue weight.

Tissues were lysed using 5ul of lysis buffer containing protease inhibitors per mg of tissue. Lysis was achieved by 3 consecutive rounds of snap-freeze/thawing, and lysates were cleared by centrifugation. Ferritin concentration in lysates was measured by ELISA, using Ferritin Elisa kit (Abcam ab157713), and were normalised to total protein concentration in each lysate. Heme concentration in lysates was measured using a Heme assay kit (Abcam ab272534) and was normalised to starting tissue weight.

Cardiac myocytes

For in-vitro bioluminescence studies, the rat cardiac myocyte cell line H9c2 (ATCC CRL1446, cardiac myoblasts from rat) was transfected with Firefly Luciferase-eGFP Lentivirus (Tetubio, product reference #14979980) and positive selection was maintained by inclusion of Geneticin in growth media. Transfected cells were maintained in complete Dulbecco′s Modified Eagle′s Medium (DMEM) growth medium, supplemented with 10% fetal bovine serum (FBS), 1% Pen Strep in standard tissue culture conditions.

Labile iron imaging in cultured cardiac myocytes

Cells were plated in complete media in opaque 96 well plates at 50000cells/well overnight. To eliminate any confounding effects of existing iron in FBS, growth media were changed to FBS-free DMEM media two hours before treatment with sterile saline or FCM, still in FBS-free media. The amount of FCM added to the FBS-free media were determined as follows; as the standard human FCM dose is 15mg iron/Kg, and the average plasma volume in humans is 60mL/Kg, the concentration of iron delivered to human plasma following a standard FCM dose is 0.25mg/mL. Thus FCM was added to the FBS-free growth media at a concentration of 0.25mg iron/mL to mimic the standard human dose.

Prior to imaging, saline and FCM-containing media were removed, cells washed with PBS, then ICL-1 added at 100uM to each well, and plates imaged immediately in an IVIS LUMINA II system, using the sequence settings described above. Luminescence data were extracted using Living Image Software, using the standard 12X8 grid ROI setting.

Haematological and iron parameters

Peripheral venous blood from participants was collected into lithium heparin tubes. Some blood was subject to haematological analysis for a full blood count using an ABX Pentra 60 system and the remainder was used to extract plasma by spinning at 2500g for 10min at 4C. Plasma samples were immediately stored at -80C^{O} for further analysis.

Haemoglobin in mice was recorded from fresh blood using the HemoCue Hb 201+ system**.** To extract serum, blood was allowed to clot at room temperature for 2 hours, before spinning at 4000g for 10 min. Serum was stored at -80C for further analysis**.**

Iron, serum ferritin and transferrin levels were determined in human plasma and mouse serum using the ABX-Pentra C400 system (Horiba). Transferrin saturation was calculated using iron and transferrin concentrations. NTBI measurement is based on the chelation-ultrafiltration-detection approach[38]. In short, serum NTBI is mobilized by the weak iron-mobilizing chelator nitrilotriacetate (NTA), after which the chelated NTBI is separated from transferrin-bound iron by ultrafiltration and measured spectrophotometrically by the addition of thioglycolic acid (TGA) and bapthophenanthroline disulfonic acid (BPT). The lower limit of detection (LLOD) of the NTBI assay is 0.607 µmol/l.

MDA levels were measured in plasma or serum using Lipid Peroxidation kit Abcam (ab233471) according to the manufacturer’s instructions.

Supplemental figures

Supplemental Figure 1

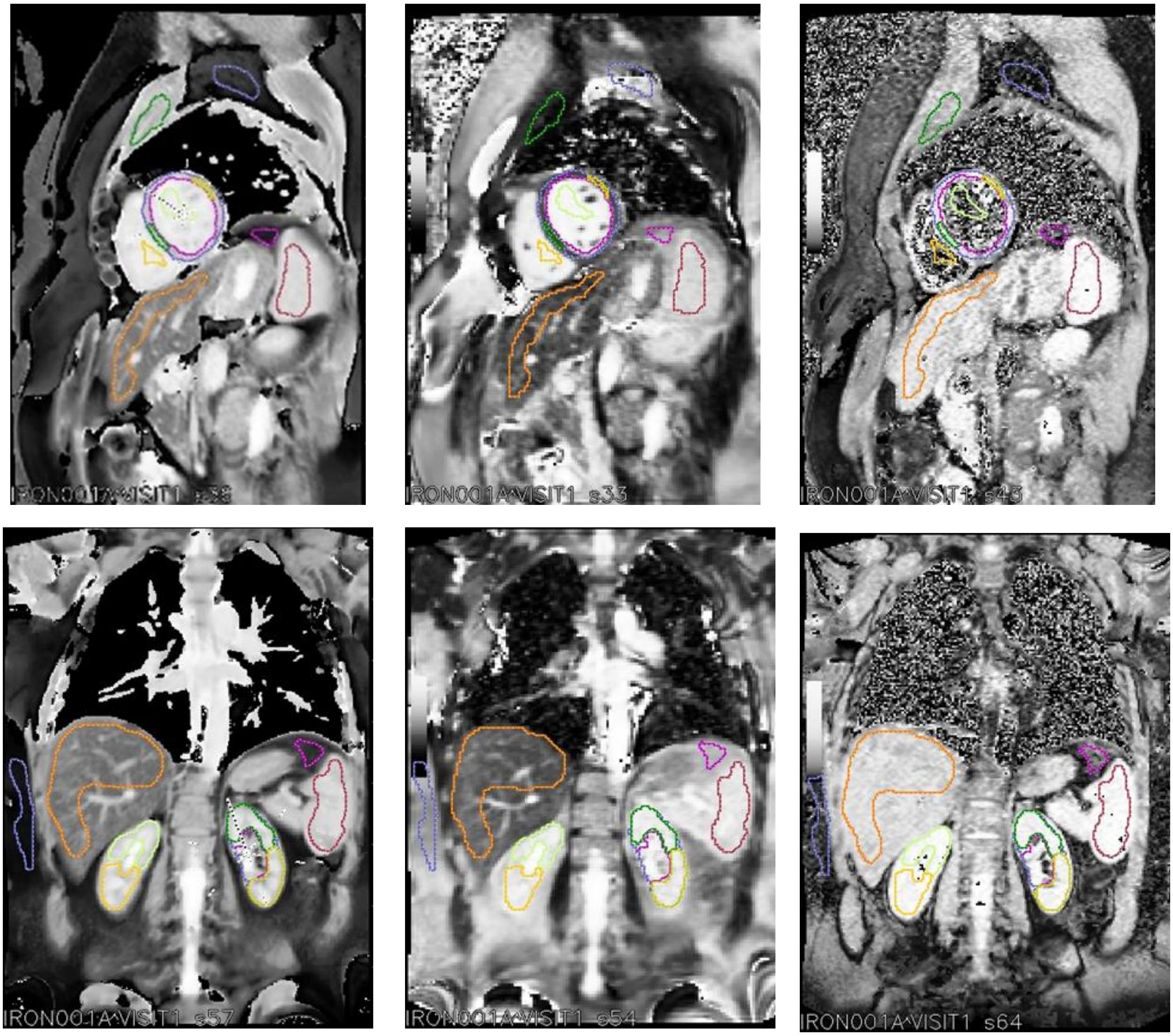

Supplemental Figure 1
Representative T1, T2 and T2* maps (left to right, respectively) for the cardiac and liver/spleen planes (top, bottom row, respectively). The coloured outlines indicate the edges of the regions of interest overlaid for the tissue of interest. Images have been histogram equalised to help identifying the distinctive tissue classes.

Supplemental Figure 2

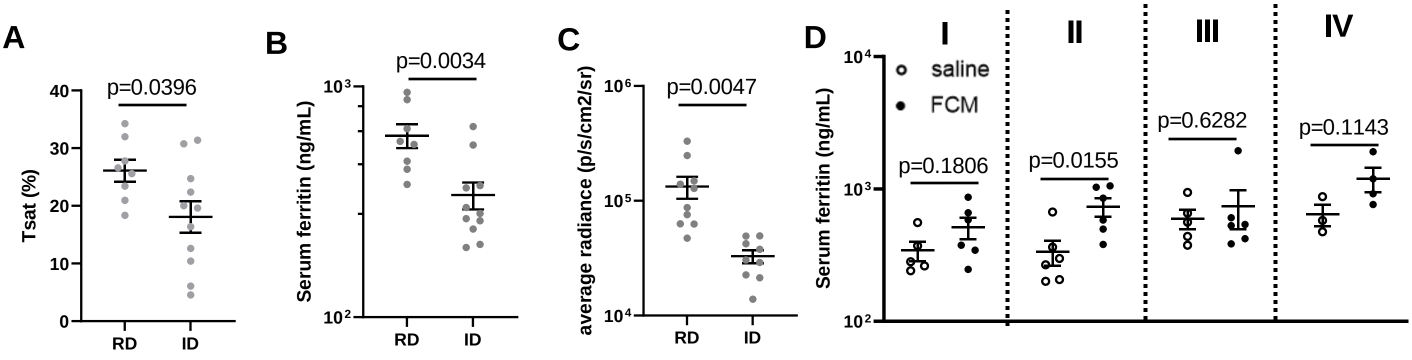

Supplemental Figure 2- Iron status of iron-replete and iron-deficient mice
Effects of provision of iron-deficient diet (ID) vs replete diet (RD) to mice on transferrin saturation (A), serum ferritin (B), and myocardial labile iron as assessed by bioluminescence (C). Serum ferritin concentrations in iron-deficient or iron replete mice 1 hour or 6 weeks after infusion of saline or FCM (15mg/kg iron) (D).

Supplemental Figure 3

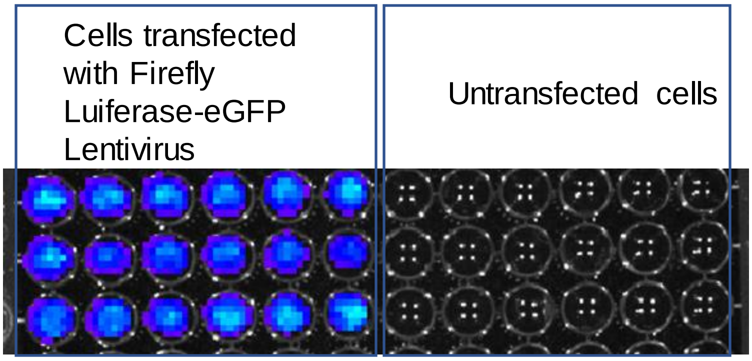

Supplemental Figure 3- Confirmation of successful transfection of rat cardiac myocytes with the firefly luciferase gene. D-luciferin substrate was added to transfected and non-transfected cells.

Supplemental figure 4

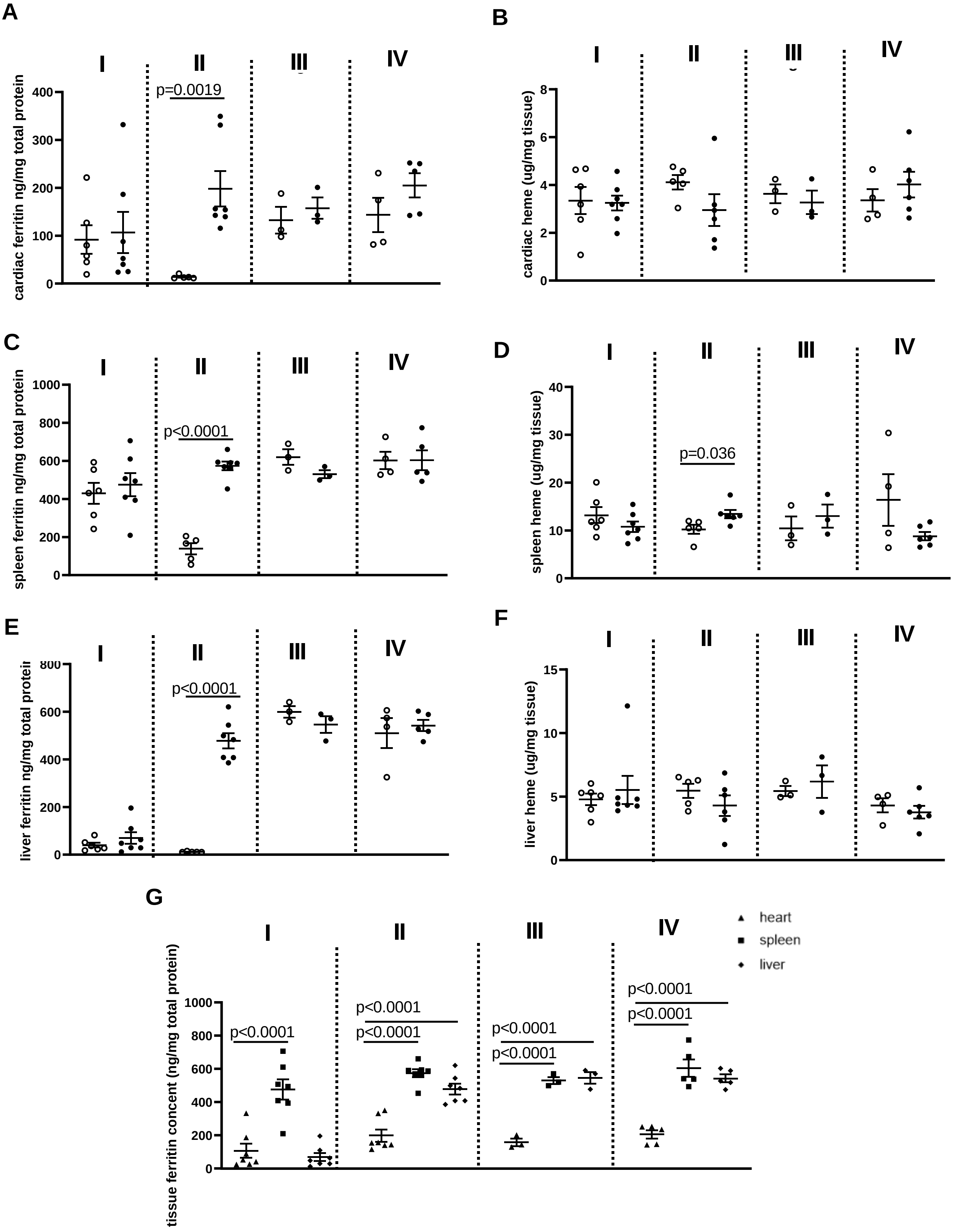

Supplemental Figure 4- Ferritin and heme content in the heart, liver and spleen. Saline (open circles) or FCM (closed circles) were administered to iron-deficient mice (conditions I and II) or iron replete mice (condition III and IV). Tissues were harvested 1 hour (conditions I and III) or 42 days later (conditions II and IV) for measurements of tissue ferritin in heart (A), spleen (C) and liver (E), and heme in heart (B), spleen (D) and liver (F). Ferritin content of the heart, spleen and liver is compared in FCM-treated mice (G).

Supplemental Figure 5

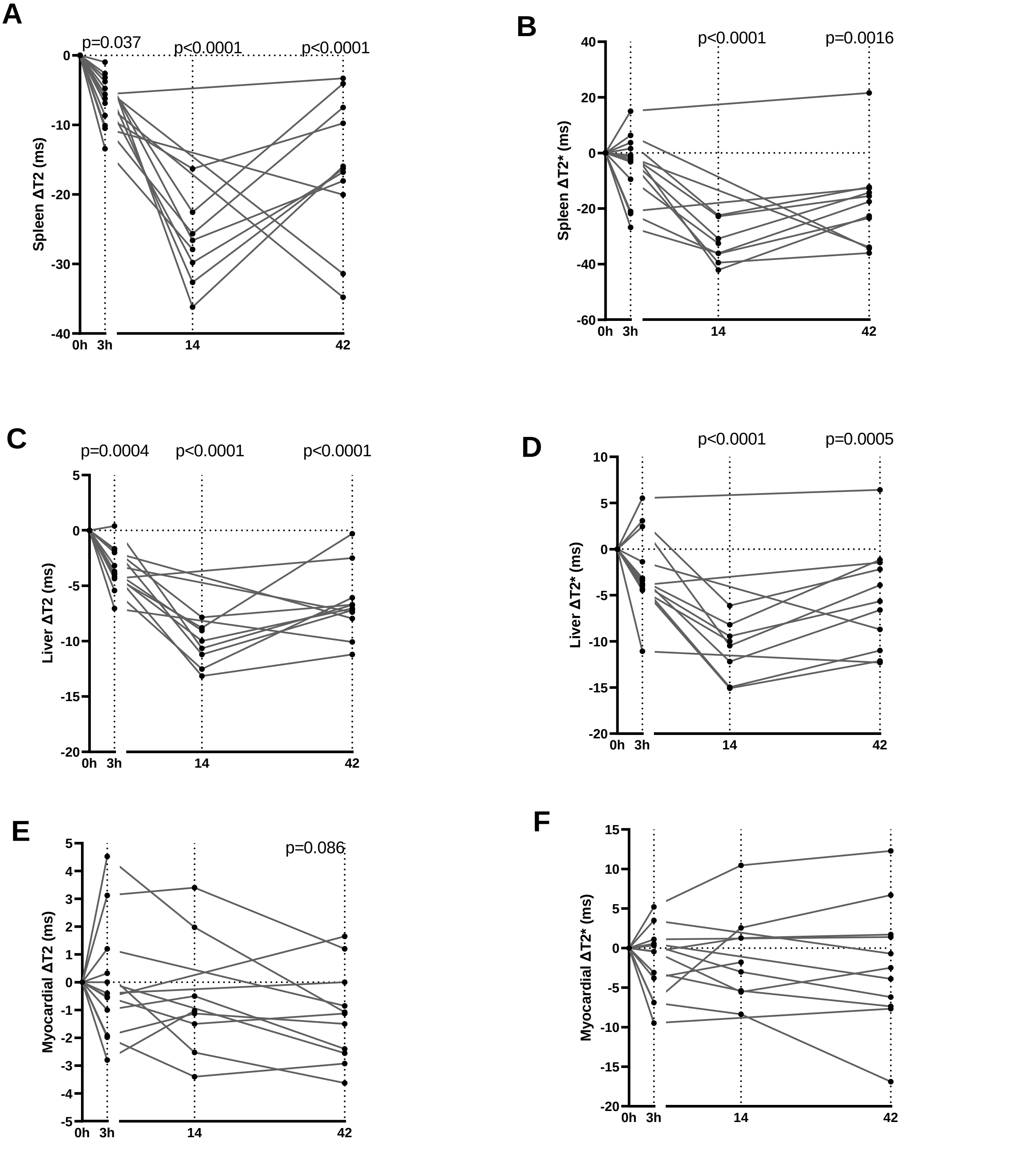

Supplemental Figure 5- Longitudinal changes in relaxometries ∆T2 and ∆T2* in patients following infusion with 15mg/kg FCM.

A, B) Longitudinal changes from baseline (∆) in splenic T2 and T2*. C, D) Longitudinal changes from baseline (∆) in liver T2 and T2*. E, F) Longitudinal changes from baseline (∆) in myocardial T2 and T2*.

Supplemental table 1

	Predicted myocardial T1 (ms) based on 28.45ms drop per dose					
baseline	after 1 st dose	after 2nd dose	after 3rd dose	after 4th dose	after 5th dose	after 6th dose
998.45	970	941.55	913.1	884.65	856.2	827.75

Supplemental table 1- Predicted cumulative effects of multiple IV iron doses on myocardial T1 (ms).
Calculations showing predicted cumulative effect of 6 standard doses of IV iron on myocardial T1 based on the observed 28.45ms drop in T1 with every dose.
